# Design, Synthesis, and Biochemical and Biological Evaluation of Novel 7-Deazapurine Cyclic Dinucleotide Analogues as STING Receptor Agonists

**DOI:** 10.1021/acs.jmedchem.2c01305

**Published:** 2022-10-06

**Authors:** Zdeněk Vavřina, Pavla Perlíková, Nemanja Milisavljević, Florian Chevrier, Miroslav Smola, Joshua Smith, Milan Dejmek, Vojtěch Havlíček, Miloš Buděšínský, Radek Liboska, Lenka Vaneková, Jiří Brynda, Evzen Boura, Pavlína Řezáčová, Michal Hocek, Gabriel Birkuš

**Affiliations:** † Institute of Organic Chemistry and Biochemistry, Czech Academy of Sciences, Flemingovo Namesti 542, Prague 166 10, Czech Republic; ‡ Department of Biochemistry, Faculty of Science, Charles University, Hlavova 2030/8, Prague 128 00, Czech Republic; § Department of Organic Chemistry, Faculty of Chemical Technology, University of Chemistry and Technology, Technicka 5, Prague 166 28, Czech Republic; ∥ Department of Organic Chemistry, Faculty of Science, Charles University, Hlavova 2030/8, Prague 128 00, Czech Republic; ⊥ First Faculty of Medicine, Charles University, Katerinska 1660/32, Prague 121 08, Czech Republic; # Department of Cell Biology, Faculty of Science, Charles University, Vinicna 1594/7, Prague 128 43, Czech Republic

## Abstract

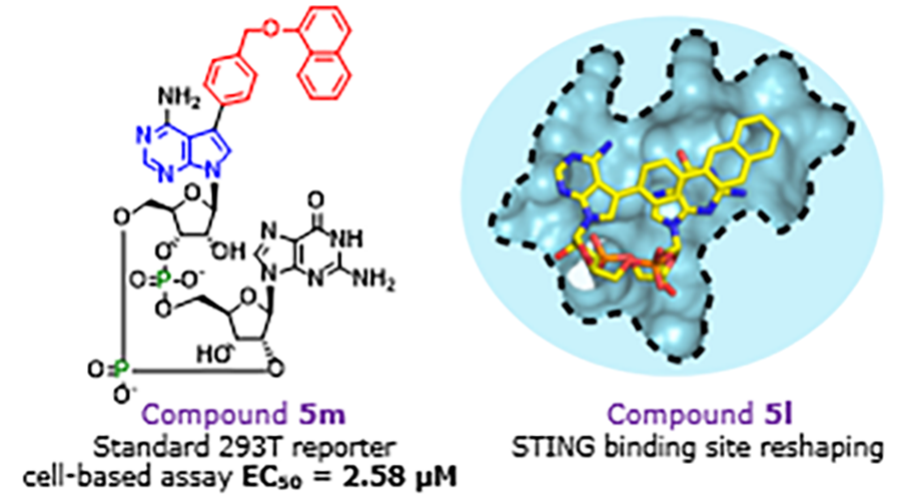

Cyclic dinucleotides (CDNs) are second messengers that activate stimulator of interferon genes (STING). The cGAS-STING pathway plays a promising role in cancer immunotherapy. Here, we describe the synthesis of CDNs containing 7-substituted 7-deazapurine moiety. We used mouse cyclic GMP–AMP synthase and bacterial dinucleotide synthases for the enzymatic synthesis of CDNs. Alternatively, 7-(het)aryl 7-deazapurine CDNs were prepared by Suzuki–Miyaura cross-couplings. New CDNs were tested in biochemical and cell-based assays for their affinity to human STING. Eight CDNs showed better activity than 2′3′-*c*GAMP, the natural ligand of STING. The effect on cytokine and chemokine induction was also evaluated. The best activities were observed for CDNs bearing large aromatic substituents that point above the CDN molecule. We solved four X-ray structures of complexes of new CDNs with human STING. We observed π–π stacking interactions between the aromatic substituents and Tyr240 that are involved in the stabilization of CDN-STING complexes.

## Introduction

**Scheme 1:**
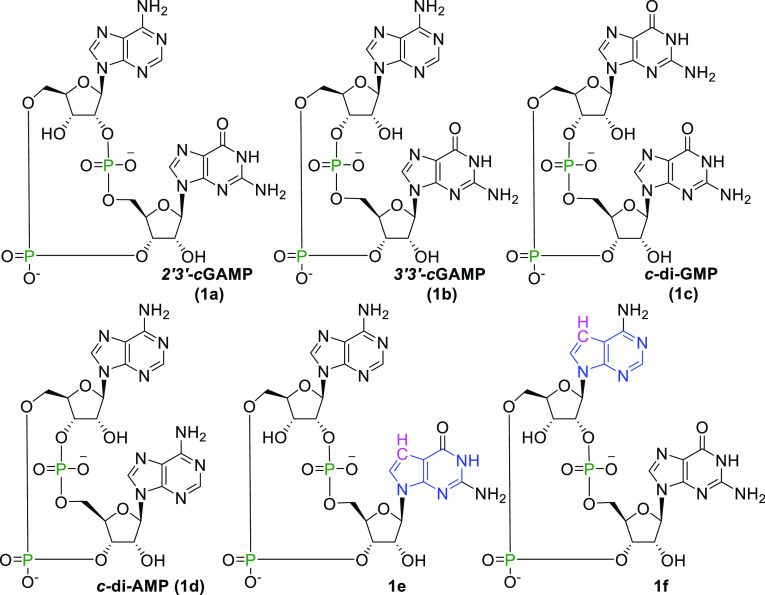
Naturally Occurring CDNs (**1a–d**) and 7-Deaza Variants (**1e**,**f**) Reported in Our Previous Study^20^

The cyclic GMP–AMP synthase (cGAS)–stimulator of interferon genes (STING) pathway is an important player in detecting damage-associated (DAMPs) and pathogen-associated molecular patterns (PAMPs).^1−4^ Upon dsDNA detection in cytosol, cGAS synthetizes a STING ligand 2′3′-*c*GAMP (**1a**, cyclic [G(2′,5′)pA(3′,5′)p] with mixed 2′–5′ and 3′–5′ phosphodiester linkages).^5,6^ Besides eukaryotic 2′3′-*c*GAMP, STING can be also activated by bacterial cyclic dinucleotides (CDNs) such as 3′3′-*c*GAMP (**1b**), *c*-di-GMP (**1c**), and *c*-di-AMP (**1d**), containing two 3′–5′ phosphodiester bonds (Scheme 1).^7−10^ Binding of CDNs to STING triggers a downstream response that results in the expression of proinflammatory cytokines (TNF-α, IL-1β) via a nuclear factor κ-light-chain enhancer of activated B-cells (NF-κB) and/or the expression of type I interferons (IFN-α, IFN-β) via an interferon regulatory factor 3 (IRF3).^11−15^ When taking into consideration this response, STING plays a crucial role in defense against pathogen infections, immune surveillance of tumor cells, and maintenance of the normal immune functions of the body.^16−19^

Human STING agonists^21,22^ can be divided into two major groups: (1) CDNs and their derivates (ADU-S100,^23^ MK-1454,^24^ TAK-676,^25^ etc.^26^) and (2) synthetic non-nucleotide STING agonists (diABZI,^27^ G10,^28,29^ MSA-2,^30^ etc.^31,32^). Due to the importance of the cGAS-STING pathway, there has been an increased interest in identifying new STING agonists with improved drug-like properties compared to the natural STING ligands. This attention is highlighted by the fact that seven different STING agonists are presently being investigated in different phases of clinical trials.^22^

Herein, we report the design, synthesis, and biochemical and biological evaluation of 7-substituted 7-deazapurine CDNs that activate STING signaling. As we reported in our previous study,^20^ 7-deazapurine CDN (Scheme 1) can be tolerated when forming the STING-CDN complex. In an effort to further probe CDN–STING interactions, we decided to explore the effect of 7-substitutions on the activity of CDNs by preparing 24 new 7-substituted 7-deazapurine CDNs. Considering the results of the initial trials, we decided to focus mainly on 7-deazaadenine derivatives due to the fact that the presence of 7-deazaguanine in compound **1e** caused a substantial drop in affinity to wt hSTING.^20^ When possible, compounds were prepared enzymatically by mouse cGAS (mcGAS) as previously shown or with bacterial dinucleotide cyclase from *Vibrio cholerae* (DncV) and diadenylate cyclase from *Bacillus thuringiensis* (DisA).^20,33−35^ Otherwise, the compounds were prepared by chemical synthesis, either by arylation of an enzymatically prepared precursor or by total synthesis. All compounds were tested by differential scanning fluorimetry (DSF) and in cell-based assays. For four of the compounds, we determined their structures in complex with STING by X-ray crystallography to better understand their binding mode.

## Results and Discussion

### Enzymatic Synthesis

**Scheme 2:**
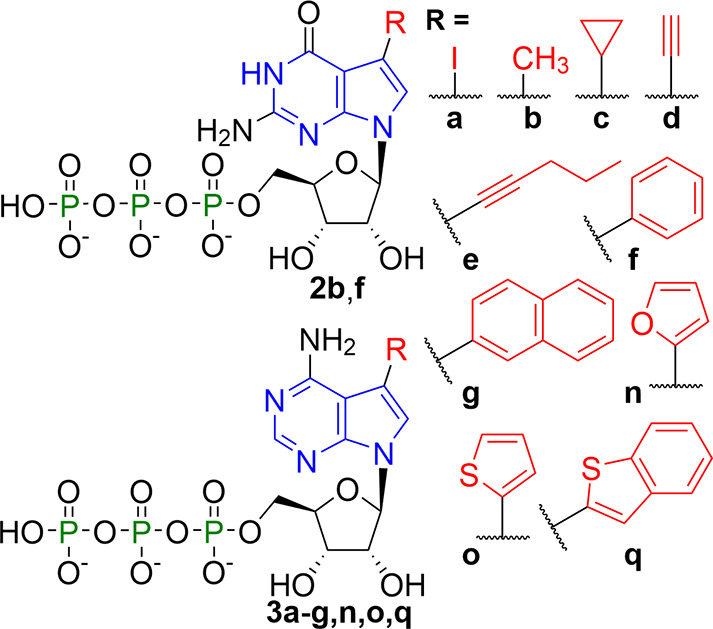
7-Deazapurine NTPs for Enzymatic Synthesis

**Scheme 3:**
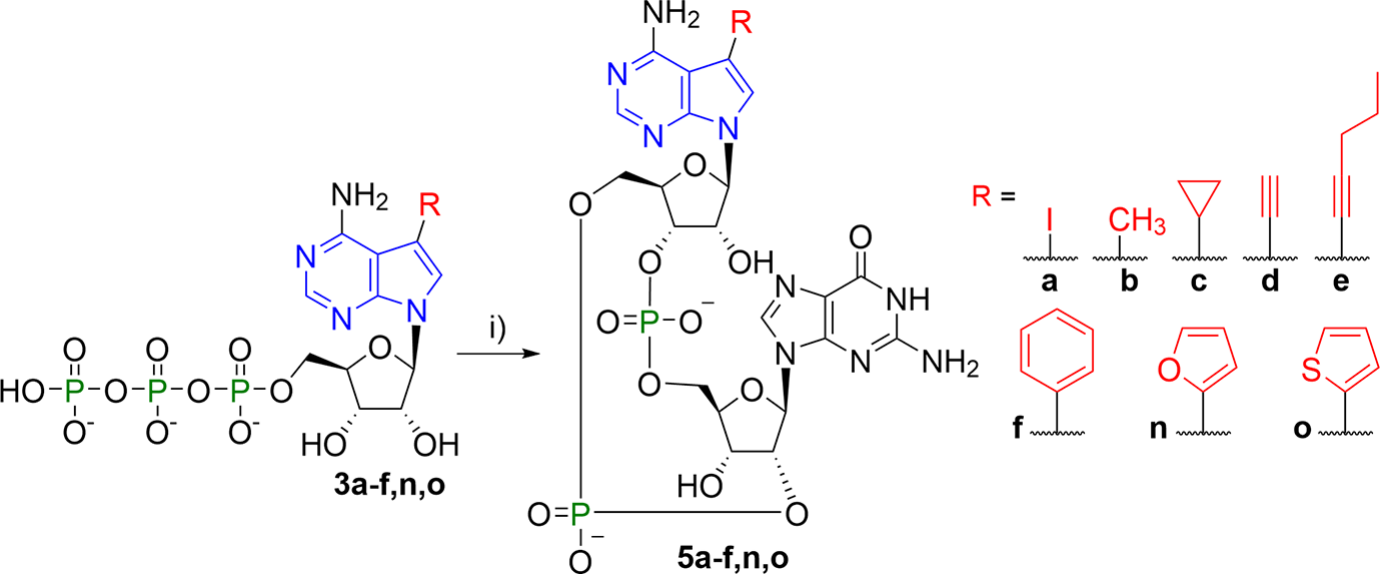
Enzymatic Synthesis of 2′3′-*c*GA^R^MPs Reagents and conditions: (i) GTP, Tris–HCl [pH 8.0], MgCl_2_, dsDNA, mcGAS, 37 °C 16 h.

**Scheme 4:**
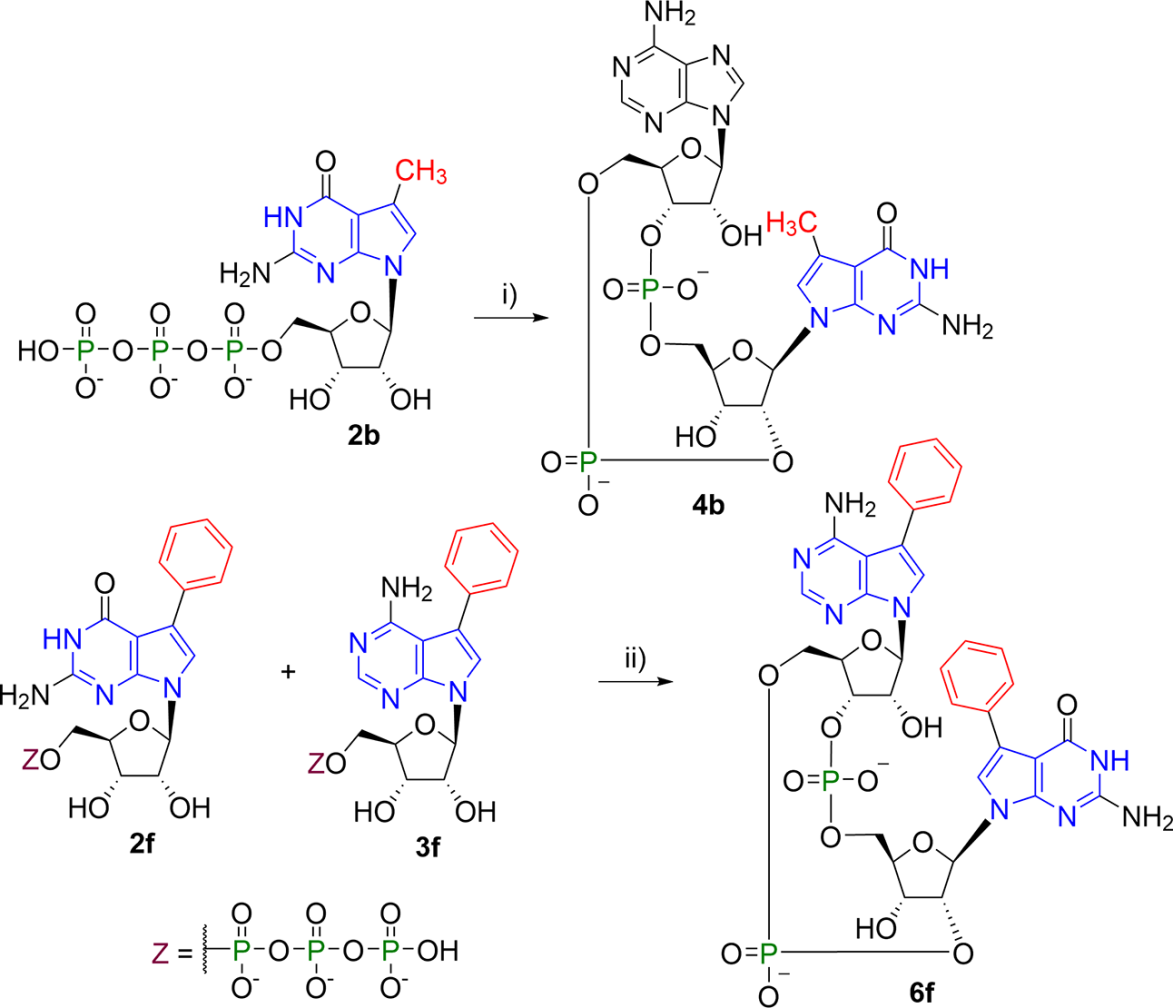
Enzymatic Synthesis of 7-Deazaguanine Containing CDNs Reagents and conditions: (i) ATP, Tris–HCl [pH 8.0], MgCl_2_, dsDNA, mcGAS, 37 °C 16 h; (ii) Tris–HCl [pH 8.0], MgCl_2_, dsDNA, mcGAS, 37 °C 16 h.

**Scheme 5:**
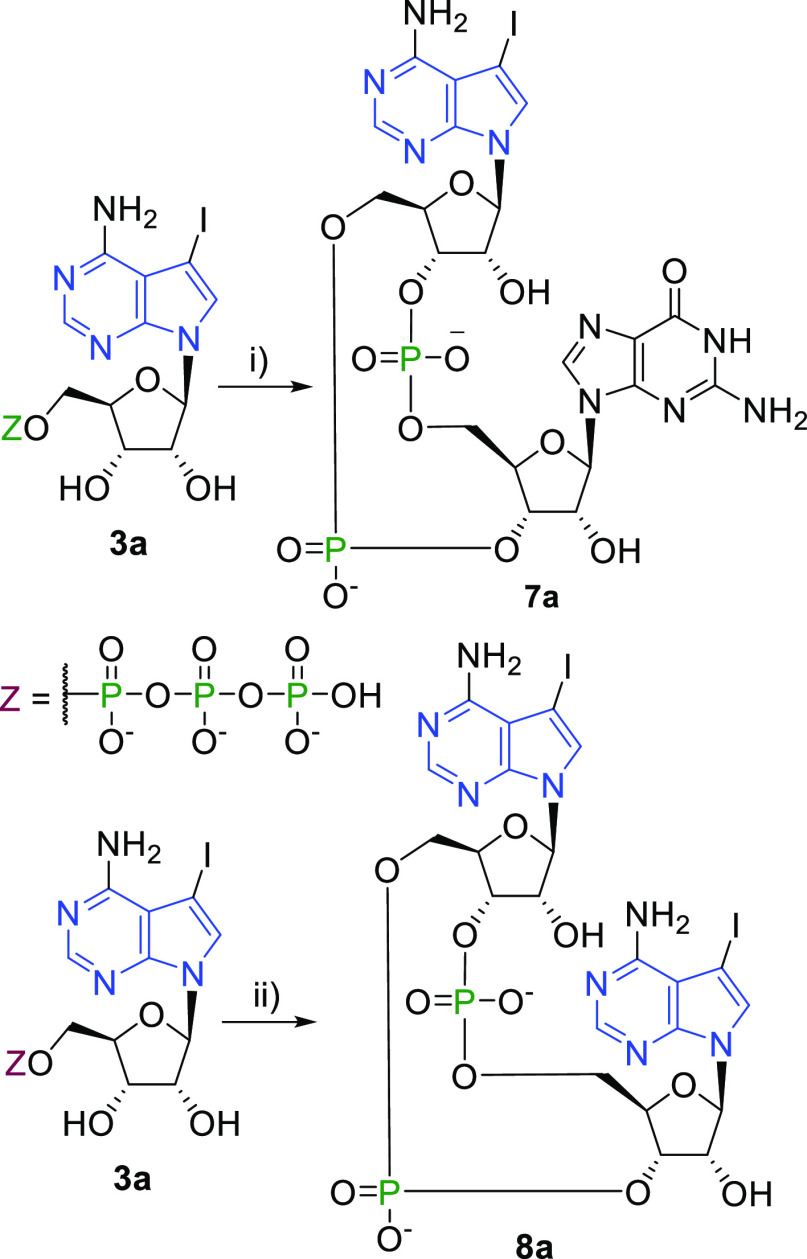
Enzymatic Synthesis with Bacterial Enzymes Reagents and conditions: (i) GTP, HEPES [pH 8.0], MgCl_2_, NaCl, DTT, DncV, 37 °C 16 h; (ii) HEPES [pH 8.0], MgCl_2_, NaCl, DTT, DisA, 50 °C 16 h.

As shown in previous works, cGAS and bacterial dinucleotide cyclases DncV and DisA can be used for the synthesis of CDNs by employing various NTP analogues.^20,33,34^ This allows for the substitution of a long and multi-step total synthesis of CDNs^36^ with a one-step enzymatic reaction. Only the enzymatic synthesis of CDNs from nucleoside triphosphates (NTPs) bearing small nucleobase modifications has been studied so far.^33^ Therefore, we decided to explore 7-substituted 7-deazaguanosine triphosphates **2** (G^R^TPs) and 7-substituted 7-deazaadenosine triphosphates **3** (A^R^TPs) as substrates for these enzymes (Scheme 2). Successful enzymatic synthesis of CDNs **4b** (2′3′-*c*G^R^AMP), **5a**-**f**,**n**,**o** (2′3′-*c*GA^R^MPs), and **6f** (2′3′-*c*G^R^A^R^MP) indicates that mcGAS tolerates small substituents in position 7 of 7-deazapurine NTPs including halogen, alkyl, cycloalkyl, alkynyl, small hetaryl, and phenyl groups (Schemes 3 and 4). Unfortunately, NTPs with aryls bigger than phenyl, that is, 2-naphthyl (**3g**) and 2-benzothienyl (**3q**),^37^ caused a disruption of the enzymatic reaction, leaving triphosphates unreacted. In some cases, such as CDN **5a**, the conversions were quantitative; thus, we were able to enzymatically prepare more than 70 mg of this CDN in one batch, which was further arylated as described below. For the synthesis of the 7-substituted 3′3′-CDNs **7a** and **8a**, we used the bacterial enzymes DncV and DisA (Scheme 5).

### Chemistry

**Scheme 6:**
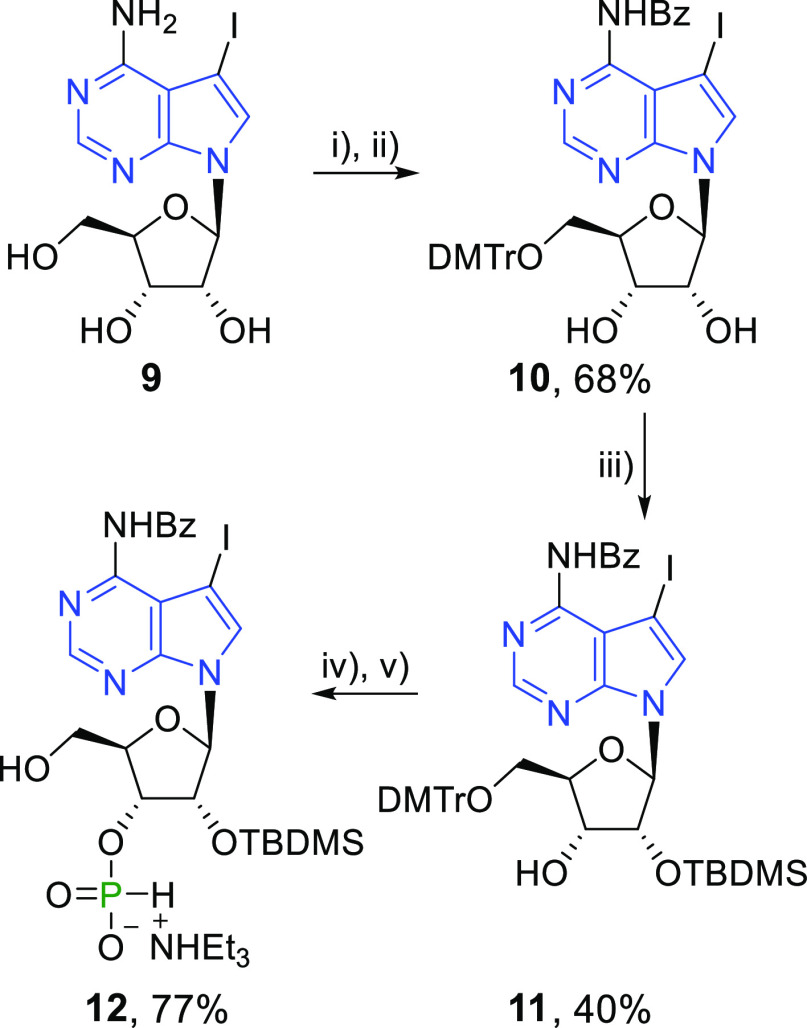
Reagents and conditions: (i) TMSCl, BzCl/py, 0 °C—rt, 16 h; (ii) DMTrCl/py, rt, 16 h; (iii) TBDMSCl, AgNO_3_, py/THF, rt 16 h; (iv) (1) PO(OPh)_2_/py, rt, 1 h, (2) H_2_O, rt, 5 min; (v) (1) DCA/DCM, rt, 15 min, (2) TES, rt, 30 min.

Chemical synthesis of 7-aryl-7-deazaadenine CDNs relied on a modular approach. The key intermediates, 7-iodo-7-deazaadenine CDNs **5a**, **7a**, and **8a**, were converted to the desired aryl-CDNs using Suzuki–Miyaura cross-coupling reactions. The iodinated CDNs can be prepared by chemical synthesis (i.e., **5a**) and/or enzymatic synthesis (i.e., **5a**, **7a**, and **8a**). The chemical synthesis of iodinated 7-deazaadenine CDN **5a** started from 7-iodo-7-deazaadenosine (**9**). First, H-phosphonate **12** had to be synthesized (Scheme 6). Iodinated nucleoside **9** was *N*-benzoylated using transient silyl protection.^38^ The *N*-benzoylated intermediate was not isolated due to its poor solubility that complicated its chromatographic purification. The crude product was directly used in a tritylation step to obtain 5′-*O*-DMTr-protected nucleoside **10** (68% over two steps). Regioselective 2′-*O*-silylation using silver nitrate catalysis^39^ provided nucleoside **11**. 3′-*O*-Silyl isomer was also formed during the reaction as a minor product, but it could not be efficiently separated from the 2′-*O*-silyl isomer **11**. However, the mixture of silylated products could be easily deprotected using TBAF in THF to regenerate the starting material **10**. 3′-*H*-Phosphonate moiety was installed by the reaction of **11** with diphenyl phosphite. The crude product was directly used for the next step in order to avoid a loss of the material during column chromatography. After the removal of the DMTr-group by DCA, *H*-phosphonate **12** was obtained as a triethylammonium salt (77% over two steps).

**Scheme 7:**
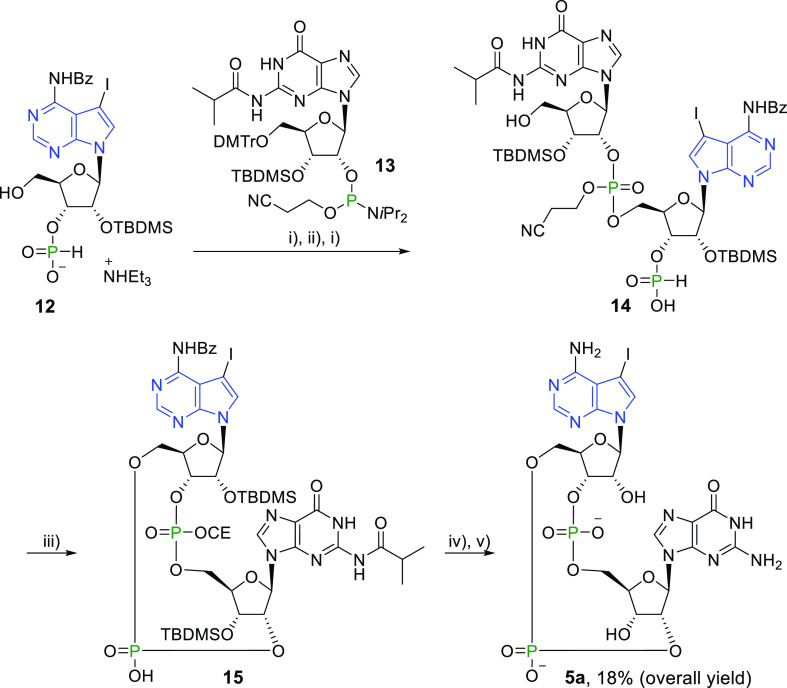
Reagents and conditions: (i) DCA/DCM, rt, 10 min; (ii) (1) **13**/MeCN, rt, 10 min, (2) *t*BuOOH, rt, 30 min; (iii) (1) DMOCP/py, rt, 110 min, (2) I_2_, H_2_O, rt, 10 min; (iv) CH_3_NH_2_/EtOH, rt, 3 h; (v) Et_3_N·3HF/py, Et_3_N, 50 °C, 3.5 h.

The reaction of *H*-phosphonate building blocks with phosphoramidites is typically performed in acetonitrile in the presence of a coupling activator,^40^ such as py-TFA. However, due to the low solubility of **12** in acetonitrile, its reaction with guanosine phosphoramidite **13** proceeded poorly. Efforts to improve the reaction yield by increasing the amount of py-TFA or by the use of other coupling activators, such as ethylthio-1*H*-tetrazole (ETT), failed. However, when **12** was treated with DCA in order to convert the triethylammonium salt to a standard *H*-phosphonate, the solubility in acetonitrile improved, and the reaction with guanosine phosphoramidite **13** proceeded even without any coupling activator. After detritylation, linear dinucleotide **14** was partially purified using reverse phase flash chromatography. The crude linear product **14** was cyclized using 2-chloro-5,5-dimethyl-1,3,2-dioxaphosphorinane 2-oxide (DMOCP).^41^ After oxidation with iodine and a partial purification on a reverse phase C18 column, the protected CDN **15** was obtained as a crude material. Deprotection of nucleobases and the phosphate using CH_3_NH_2_ and the removal of silyl groups by Et_3_N·3HF provided 7-iodinated 7-deazaadenine CDN **5a** in an overall yield of 18% (starting from *H*-phosphonate **12**, Scheme 7).

**Figure 1:**
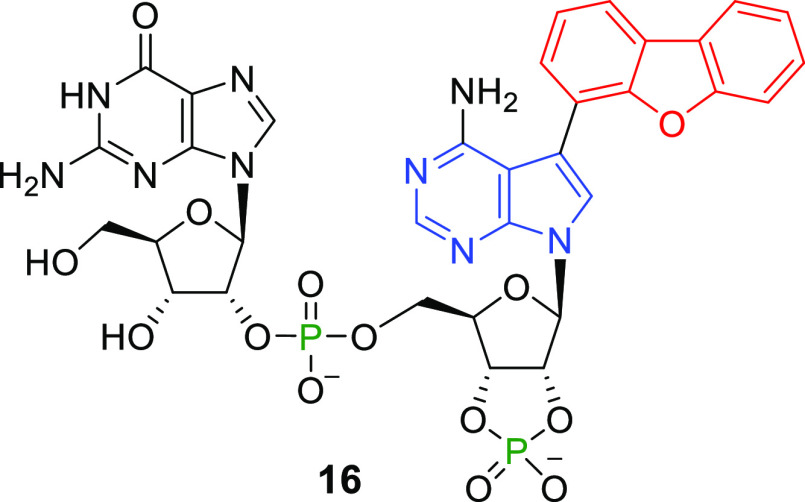
Open isomerized side product of a cross-coupling reaction.

**Scheme 8:**
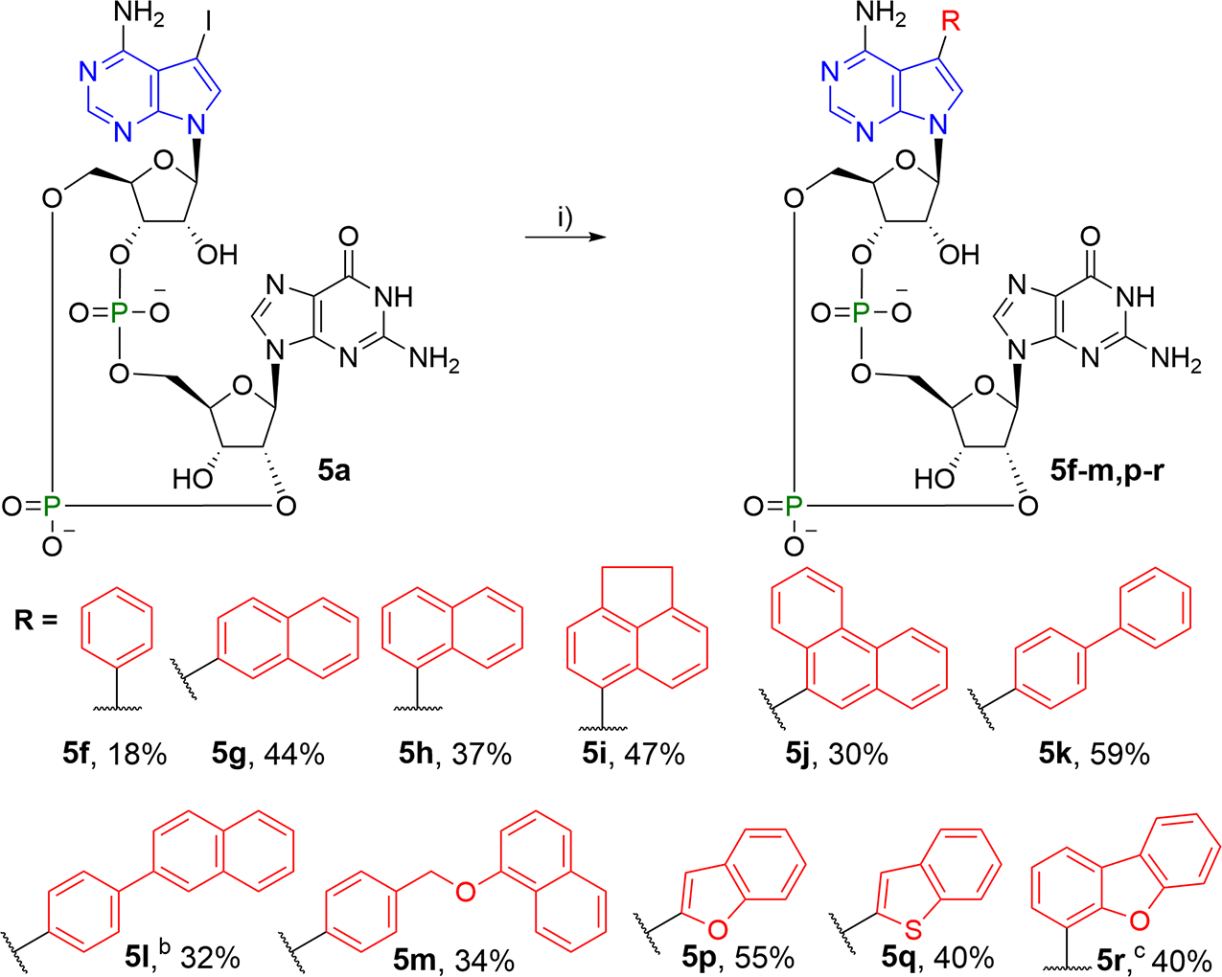
Reagents and conditions: (i) RB(OH)_2_, Cs_2_CO_3_, TPPTS, Pd(OAc)_2_/H_2_O–MeCN (2:1), 100 °C, 30 min. ^b^RBpin was used instead of RB(OH)_2_. ^c^Open isomer **16** (13%) was also isolated.

The Suzuki–Miyaura cross-coupling reaction is a generally used method for the introduction of aryl and hetaryl groups into the position 7 of 7-deazapurine nucleotides.^42^ Hydrolysis of the phosphodiester backbone was observed during the synthesis of 8-arylguanosine-containing CDNs using Suzuki–Miyaura cross-coupling.^43^ Therefore, we decided to use reaction conditions that were originally optimized for the synthesis of 7-deazapurine NTPs that also suffer from hydrolytic unstability.^44,45^ A short reaction time (30 min) was crucial in order to avoid excessive hydrolysis. The cross-coupling reactions were performed under Pd(OAc)_2_ catalysis in the presence of a water-soluble ligand, triphenylphosphan-3,3′,3″-trisulfonate (TPPTS), and Cs_2_CO_3_ in water–acetonitrile mixture (2:1). The reaction of iodinated CDN **5a** with 2 equiv of phenylboronic acid did not provide full conversion so that phenyl derivative **5f** was obtained in low yield (18%, Scheme 8). With 5 equiv of arylboronic acids (or arylboronic acid pinacol esters), the cross-couplings proceeded smoothly. Nevertheless, the yields were affected by the hydrolysis. Arylated 2′3′-*c*GA^R^MPs **5f–m** and **p–r** were prepared in 30–59% yield (Scheme 8). From the reaction with dibenzofuran-4-ylboronic acid, isomerized side product **16** (Figure 1) was obtained in 13% yield. Similar byproducts were observed in all cross-coupling reactions, but the byproducts were not isolated. Due to hindered rotation at room temperature, CDNs **5h–j** were prepared as inseparable mixtures of diastereomers/atropoisomers. Hindered rotation of bulky aryl substituents has been reported among corresponding 7-aryl-7-deazaadenosines.^46^

**Scheme 9:**
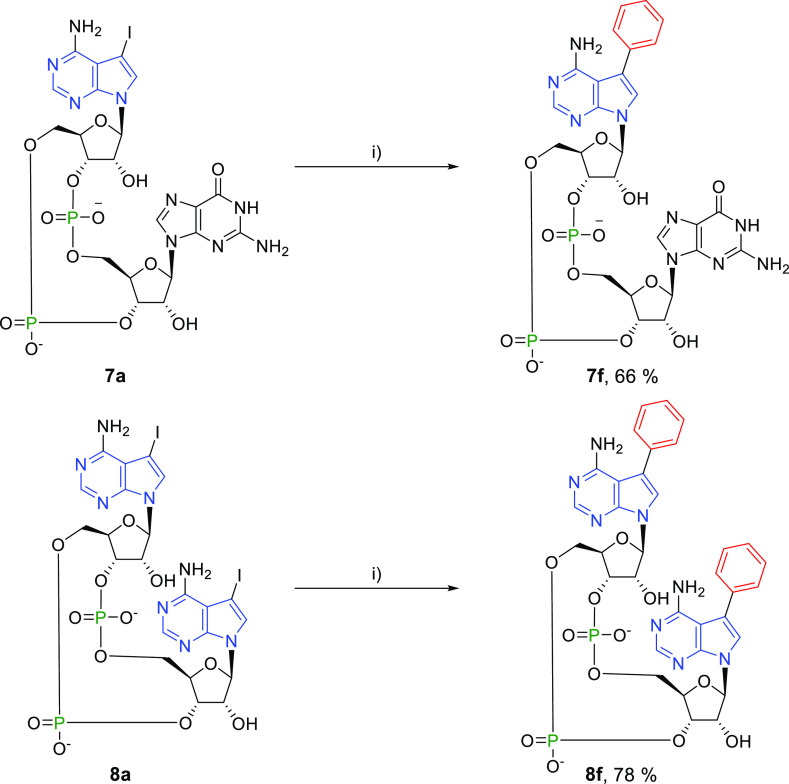
Reagents and conditions: (i) PhB(OH)_2_, Cs_2_CO_3_, TPPTS, Pd(OAc)_2_/H_2_O–MeCN (2:1), 100 °C, 30 min.

The conditions for the Suzuki–Miyaura cross-coupling reaction were also applied for the synthesis of 3′3′-CDNs. Iodinated CDNs **7a** and **8a** were converted to corresponding phenyl derivatives **7f** (66%) and **8f** (78%), respectively (Scheme 9). In these reactions, higher yields were achieved because the hydrolysis rate of 3′3′-CDNs during the cross-coupling reaction was significantly lower compared to that of 2′3′-CDNs.

### Biochemistry and Biology

In order to biochemically and biologically characterize prepared CDNs, all CDNs were tested using DSF and 293T cell-based reporter assays. The selected compounds that showed activity were further evaluated for the induction of cytokines using human peripheral blood mononuclear cells (PBMCs). DSF was performed using wild-type human STING protein (UniProt Q86WV6). This method provides a useful insight into the STING binding properties of CDNs by determining their ability to improve the thermal stability of the protein.^47,48^ Digitonin 293T cell-based assays were performed using five major allelic variants (wild type, HAQ, AQ, Q, and REF)^49^ in the presence of digitonin A that facilitates entry of CDNs into cells due to the permeabilization of cell membranes.^20^ The standard format of the 293T cell assay was carried out only with cells expressing wild-type human STING and in the absence of the permeabilizing agent.^20^

Our initial experiments with 7-deaza substituted 2′3′-*c*GAMP showed that 7-methyl is much less tolerated on 7-deazaguanine (**4b**) than on 7-deazaadenine (**5b**) (Table 1). 7-Deazaguanine-modified **4b** had Δ*T*_m_ in a DSF assay of 3.3 °C while 7-deazaadenine-modified **5b** of 11.5 °C. This suggests that the noncovalent interactions of the NH group at position 7 of the nucleobases have a bigger impact on the CDN binding to STING for guanine than for adenine. The results are in agreement with the data for the 7-deaza modification that we reported on earlier.^20^ These findings were further confirmed by improved activity of **5b** than of **4b** in the 293T cell-based reporter assay (Table 1). This led to focusing our efforts on the synthesis of 2′3′-*c*GA^R^MP-derived CDNs. We prepared compounds **5c–r**, each containing a substituent of different size at position 7 of 2′3′-*c*GA^R^MP. Surprisingly, STING turned out to tolerate not only relatively small substituents, that is, iodine (**5a**), cyclopropyl (**5c**), and ethynyl (**5d**), but also small aromatic substituents, that is, phenyl (**5f**), 2-furyl (**5n**), and 2-thienyl (**5o**) substituents, and even relatively large aromatic groups such as 2-naphthyl (**5g**), 4-biphenylyl (**5k**), 4-(2-naphthyl)phenyl (**5l**), 4-[(2-naphthyloxy)methyl]phenyl (**5m**), 2-benzofuryl (**5p**), and 2-benzothienyl (**5q**).

**Table 1:**
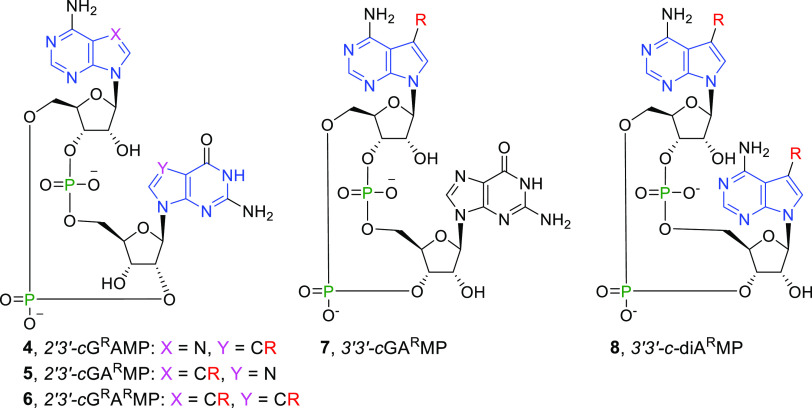
Activities of Synthesized 7-Deazapurine CDNs in DSF and Cell-Based Assays

		DSF Δ*T*_m_ (°C)^a^	digitonin assay EC_50_ (μM)^b^					standard assay EC_50_ (μM)^c^
compound	R	wt	wt	HAQ	REF	AQ	Q	wt
**4b**	methyl	3.3	3.01	1.38	13.34	1.37	2.54	>150
**5a**	I	8.3	0.066	0.127	2.48	0.097	2.30	>150
**5b**	methyl	11.5	0.009	0.082	0.189	0.080	0.275	31.30
**5c**	cyclopropyl	10.8	0.064	0.403	0.379	0.291	0.302	>150
**5d**	ethynyl	8.7	0.020	0.132	1.09	0.111	0.885	>150
**5e**	pent-1-ynyl	6.7	0.116	0.243	6.77	0.181	2.71	112.9
**5f**	phenyl	8.1	0.063	0.385	5.90	0.307	1.70	40.7
**5g**	2-naphthyl	14.4	0.048	0.695	1.40	0.550	0.700	7.63
**5h**	1-naphthyl	4.4	1.10	0.497	18.90	0.470	7.23	42.45
**5i**	5-acenaphthenyl	2.5	1.16	1.63	>45	1.53	35.85	>150
**5j**	9-phenanthrenyl	4.4	0.061	0.900	>45	1.40	>45	65.45
**5k**	4-biphenylyl	12.3	0.013	0.173	0.346	0.092	0.235	2.95
**5l**	4-(2-naphthyl)phenyl	13.0	0.032	0.132	1.00	0.160	0.950	3.06
**5m**	4-[(2-naphthyloxy)methyl]phenyl	13.1	0.043	0.113	1.75	0.095	1.29	2.58
**5n**	2-furyl	8.9	0.025	0.163	0.816	0.115	0.469	41.30
**5o**	2-thienyl	9.8	0.028	0.174	0.647	0.184	1.85	27.30
**5p**	2-benzofuryl	12.1	0.076	0.060	0.334	0.085	0.176	10.94
**5q**	2-benzothienyl	13.5	0.035	0.195	0.350	0.330	0.250	8.69
**5r**	4-dibenzofuryl	7.1	0.367	0.503	8.97	0.869	7.07	19.10
**6f**	phenyl	1.0	6.41^d^	9.42^d^	19.49^d^	10.30^d^	0.271^d^	>150^d^
**7a**	I	4.4	0.101	0.896	>45	0.780	12.85	>150
**7f**	phenyl	5.1	0.110	0.367	10.37	0.280	4.480	>150
**8a**	I	0.5	3.488	>45	>45	>45	>45	>150
**8f**	phenyl	–0.6	>45	>45	>45	>45	>45	>150
2′3′-*c*GAMP		15.3	0.020	0.021	0.074	0.041	0.048	28.37
3′3′-cGAMP		5.1	0.121	0.123	4.26	0.260	2.06	68.37
2′2′-cGAMP		2.5	0.260	0.189	59.54	0.173	7.09	>150
ADU-S100		9.3^e^	0.08^e^	0.26^e^	1.64^e^	0.23^e^	1.01^e^	3.32^e^

a Values were obtained using DSF assay with wt hSTING as described in the Methods section. Measurements were performed as two independent experiments (*n* = 2).

b Results of digitonin assay in 293T reporter cells expressing hSTING allelic variants were obtained as described in the Methods section. EC_50_ values are the mean of three independent experiments (*n* = 3) measured in triplicate with SD < 50% of EC_50_ values.

c Results of standard assay in 293T reporter cells expressing wt hSTING were obtained as described in the Methods section. EC_50_ values are the mean of two independent experiments (*n* = 2) measured in triplicate with SD < 50% of EC_50_ values.

d Data from one experiment only (*n* = 1).

e Data from Dejmek et al.^50^

When considering Δ*T*_m_, for **5c**, it decreased by only 0.7 °C, whereas **5d** showed a nearly 3 °C drop compared to **5b**. This minor decrease suggests that in position 7 of 7-deazaadenine, there is a space for the introduction of larger modifications than just a methyl group. When tested in a digitonin cell-based reporter assay, modifications on both **5c** and **5d** were tolerated. However, for **5d**, the EC_50_ value obtained from an assay in cells expressing the REF allelic form increased by nearly 6-fold, whereas at all other tested allelic forms, the increase was not higher than 3-fold. This suggests a disruption of interactions between **5d** and STING when R232 is replaced by H232. In a standard cell-based reporter assay for compounds **5c** and **5d**, we observed a deterioration of cellular activity, which was not present when tested with a membrane-permeabilizing agent.

After proving that there might be space for the introduction of larger substituents in the binding site of STING, we introduced phenyl (**5f**), small heterocycles (**5n**, **5o**), and pent-1-ynyl (**5e**) into position 7 of 7-deazaadenine. Surprisingly, we found that Δ*T*_m_ for **5f**, **5n**, and **5o** decreased by less than 3 °C, and EC_50_ values in the digitonin assay for these compounds did not increase by more than 2-fold compared to **5c**. Moreover, for compounds **5f**, **5n**, and **5o**, we observed restored activity in the standard assay, which was deteriorated for **5c** and **5d** (Table 1). In fact, EC_50_ for **5o** in the assay was even slightly lower than that for 2′3′-*c*GAMP. We also solved the crystallographic structure for **5f** in complex with STING.

Considering the structure of **5f**, we hypothesized that there exists a possibility of intramolecular stabilization through π–π stacking between phenyl and guanine at **5f** that might help to improve its activity. To prove this possibility, we prepared 2′3′-*c*G^Phe^A^Phe^MP (**6f**), 3′3′-*c*GA^Phe^MP (**7f**), and 3′3′-*c*-diA^Phe^MP (**8f**). Unfortunately, substitutions with phenyl in these compounds did not show an improvement at STING in DSF or cell-based reporter assays compared to **5f**. For **7f**, we found a decrease in Δ*T*_m_ by 3 °C, but in digitonin assay, EC_50_ values for allelic form HAQ and AQ remained nearly the same. For other tested allelic forms, EC_50_ did not increase more than 2-fold compared to **5f**. However, we observed a deterioration of activity in the standard cell-based reporter assay similar to compounds **5c** and **5d**. According to data from both DSF and cell-based reporter assays, substitutions in **6f** caused a significant drop of STING activity and led to the inactivity of **8f**. Substitutions in these compounds might potentially lead to the disruption of interactions with both R238 residues, which we have already reported to significantly affect the binding of CDNs.^47^

When we considered the fact that the results for **5n** and **5o** showed better activity than compounds with phenyl substituents **5–8f**, we focused on larger heterocycles like benzofuryl (**5p**), benzothienyl (**5q**), and dibenzofuryl (**5r**). For **5p** and **5q**, we observed an increase in Δ*T*_m_ by more than 3 °C when compared to **5n** and **5o**, respectively. Higher Δ*T*_m_ suggests increasing stabilization of complex STING–CDN.^47,48^ We can only speculate that this stabilization could be related to the introduction of a benzofused heterocycle, which might be in a better position to form π–π stacking with guanine. Consistent with DSF, we observed that **5p** showed lower EC_50_ values than **5n**, except for wild-type STING. Derivative **5q** showed lower EC_50_ for REF and Q than **5o**. Compound **5r** did not show any improvement in DSF or in digitonin assay when compared to **5n**, **5o**, **5p**, or **5q**. Nevertheless, all **5p**, **5q**, and **5r** compounds showed an improvement in a standard assay compared to **5n**, **5o**, and 2′3′-*c*GAMP. In the case of **5q**, it was 3-fold better than 2′3′-*c*GAMP.

We proved that there is a space for larger modifications without a reduction in activity in position 7 of 7-deazaadenine in 2′3′-*c*GA^R^MP. When correlated with findings about improved activity in the standard assay for compounds with heterocyclic substituents, we designed compounds modified with large 2-naphthyl (**5g**), 1-naphthyl (**5h**), 5-acenaphthenyl (**5i**), and 9-phenanthrenyl (**5j**) substituents. Interestingly, derivatives **5h**, **5i**, and **5j** showed lower Δ*T*_m_ and considerably higher EC_50_s compared to **5n**, **5o**, **5p**, **5q**, and **5r**. On the other hand, **5g** showed a Δ*T*_m_ of 14.4 °C and a nearly 4-fold lowered EC_50_ in a standard cell-based reporter assay when compared to 2′3′-*c*GAMP. Unfortunately, **5g** showed lower activation of HAQ, AQ, and REF allelic forms. These results show that interactions in the STING binding pocket are more likely to tolerate substitutions pointing above the adenine moiety rather than substitutions that need additional interactions and space around the *c*GAMP molecule.

We designed a 4-biphenylyl-substituted 2′3′-*c*GA^R^MP (**5k**) by applying our findings about the preference of the STING protein in the site above AMP in 2′3′-*c*GAMP and with the knowledge of the relative flexibility of the lid above the binding.^51^ The biphenylyl substituent brings additional lipophilicity to the compound, as in the case of **5g**, but with the substitution pointing above the *c*GAMP molecule, as in the case of **5f**. This substitution caused a decrease of only 3 °C in DSF, and EC_50_s were comparable to those of 2′3′-*c*GAMP. Moreover, **5k** showed nearly 10 times better activity than 2′3′-*c*GAMP in the standard assay.

Considering the results for **5k**, we designed a substitution with 4-(2-naphthyl)phenyl (**5l**). This modification resulted in nearly the same Δ*T*_m_ and EC_50_s despite the change from phenyl to naphthyl, which might cause more significant collisions with the lid of the STING binding pocket.

Compound **5m** contained the largest modification we introduced, 4-[(2-naphthyloxy)methyl]phenyl. Despite the introduction of such a large modification that was expected to largely clash with a lid of the STING binding pocket, we observed similar complex stability for **5m** and **5l** in DSF. Moreover, we observed comparable activities in cell-based reporter assays. When comparing the activities of derivatives **5k–m** with those of the clinical candidate ADU-S100,^23,50^ similar or better activities were observed in digitonin assay. Moreover, in the standard 293T cell-based assay, compounds **5k–m** showed lower EC_50_ values than ADU-S100. To explain potent activities of compounds **5k–m**, we solved the X-ray crystallographic structure for the complex of STING and corresponding CDNs.

### Structural Studies

**Figure 2:**
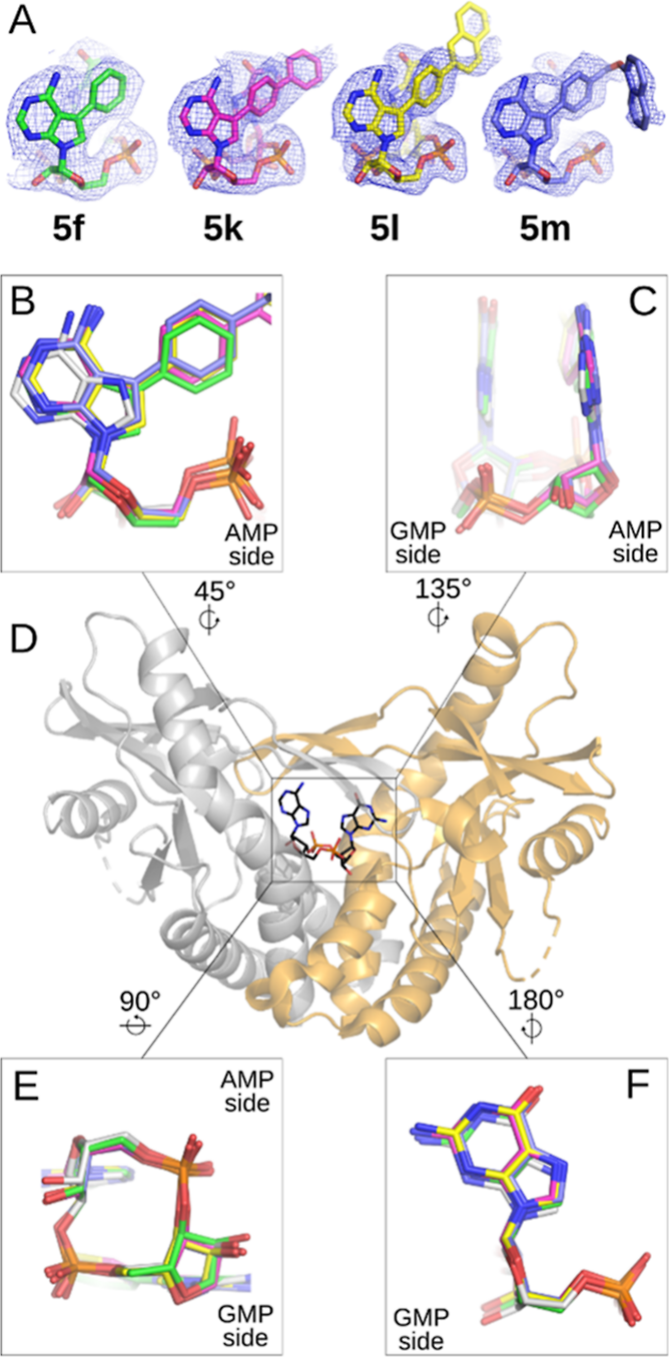
Crystal structures of human STING in complex with 2′3′-*c*GA^R^MPs. (A) Binding poses of compounds are shown with 2*Fo*–*Fc* maps contoured at 1σ. Compounds are distinguished by carbon colors (**5f** in green, PDB 8A2H; **5k** in purple, PDB 8A2J; **5l** in yellow, PDB 8A2I; and **5m** in blue, PDB 8A2K), while nitrogen, oxygen, and phosphorus atoms are colored blue, red, and orange. (B) AMP side view of **5f**, **5k**, **5l**, and **5m** binding poses superposed with 2′3′-*c*GAMP (PDB 4KSY), showing the trend in positions of 7-deazaadenosine for all of our compounds, which differs from the position of adenosine in 2′3′-*c*GAMP (in white). (C) Side view superposition of ligands showing similarity of localization of nucleobases in this orientation. (D) Human STING dimer shown as a biological unit with differently colored monomers with 2′3′-*c*GAMP located in the binding site (PDB 4KSY). (E) Bottom view and (F) GMP side view of superposed ligands.

To understand the interactions of large moieties in position 7 of 7-deazaadenine in 2′3′-*c*GA^R^MPs, we crystallized human wild-type STING truncated to residues 140–379 in complexes with **5f**, **5k**, **5l**, and **5m** using our improved STING crystallization protocols.^52^ Crystal structures were determined at resolution 2.69–1.89 Å, and the asymmetric unit consisted of two STING heterodimers (chain A/B) and one molecule of the ligand (Table S1). Protein residues were modeled into a well-defined electron density map, except for several regions belonging to flexible surface exposed loops that could not be resolved owing to their dynamic disorder (Table S2). All ligands were modeled into the binding site with full occupancy; however, some parts of the maps for substitutions of position 7 of 7-deazaadenine were not as well defined, suggesting some flexibility of this part of the molecule (Figure 2A).

**Figure 3:**
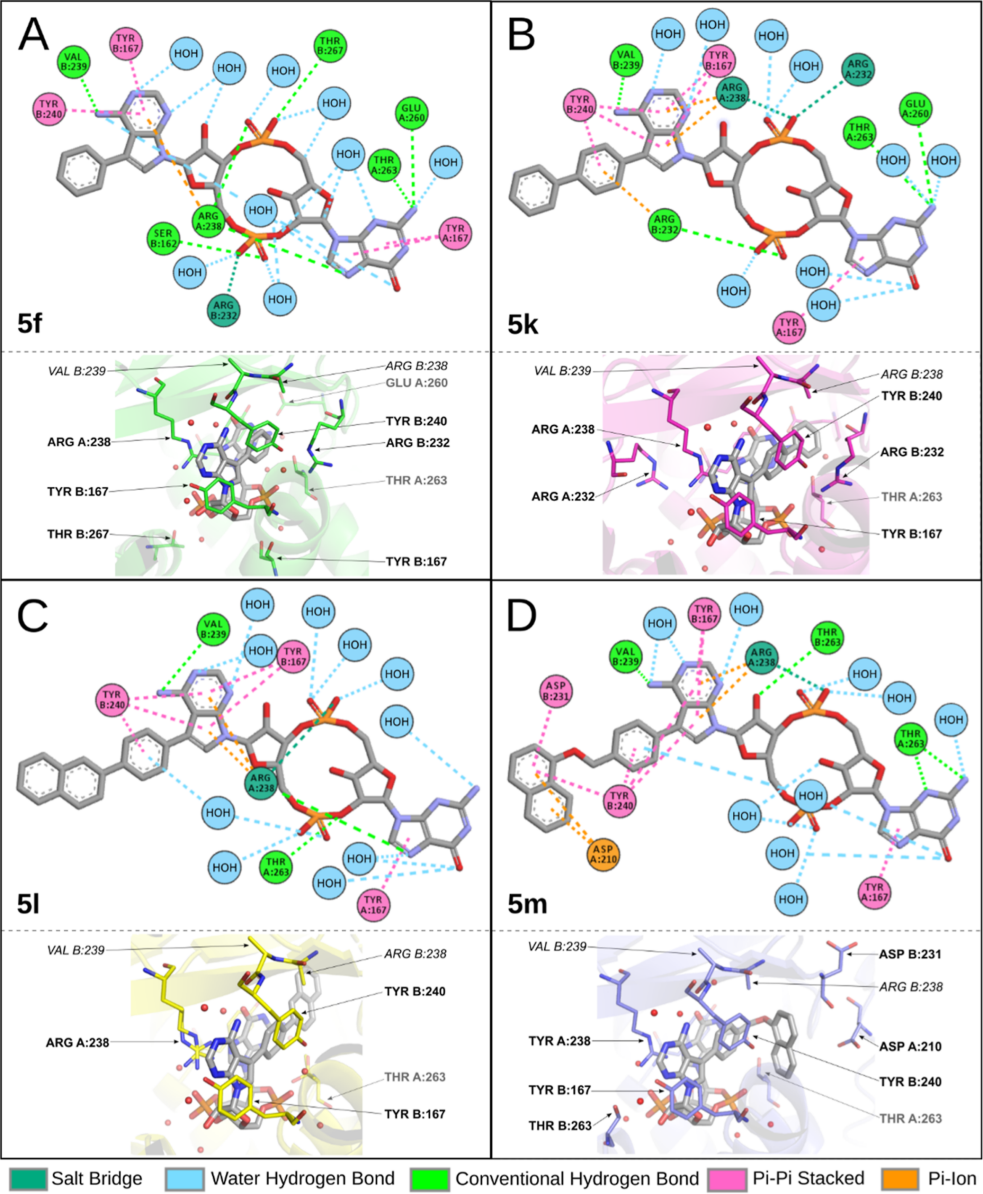
Interaction scheme of **5f** (A; PDB 8A2H), **5k** (B; PDB 8A2J), **5l** (C; PDB 8A2I), and **5m** (D; PDB 8A2K) in STING ligand-binding site, as seen in our structures.

The binding site is formed at the interface of two STING monomers and is covered by two “lids” (residues 227–241). The electron density for the lid region was mostly well defined, with some exceptions pointing to residues disordered by the binding of a voluminous ligand (residue Ile235 in **5l** complex; Arg232 in **5m** complex; and Tyr240 in **5k**, **5l**, and **5m**, Figure S1). Ligands are deeply buried in the binding site (Figure 3), with basic orientation resembling that of natural ligand 2′3′-*c*GAMP (PDB 4KSY).^5^ Most of the interactions observed in the binding site were similar to those described for a natural ligand (Figure S2): Tyr167 π–π stacking with nucleobases, Arg232 forming a salt bridge with phosphates, a side chain of Thr263 forming a hydrogen bond with NH_2_ of guanine, Arg238 interacting through a hydrogen bond with phosphates, and Arg238 cation−π stacking with nucleobases on the opposite of the binding site. Ribose moiety and the central part are linked to the protein residues through a network of water-mediated hydrogen bonds. In all our structures, we also observed Val239 carbonyl forming a hydrogen bond with NH_2_ at 7-deazaadenosine and Tyr240 π–π stacking with introduced aromatic substituents (Figure 3).

When comparing the binding pose of the natural ligand with our compounds, the GMP side for all of the compounds shows only an insignificant shift in position (Figure 2C,E,F). For the AMP side, the ribose and nucleobase are slightly shifted upward, potentially because of our large substitutions at 7-deazaadenine. The substitutions in all of our structures displace the side chain of Arg238 from chain B and thus prevent interaction with guanosine (Figure 2B,C). This shift enables direct interaction with Tyr240 from chain B that was not observed for the natural ligand binding. This residue interacts with 7-deazaadenine through parallel displaced π–π stacking. This interaction is enabled by a change of AMP positioning, and the extent of its interaction with Tyr240 differs in our structures due to the different sizes of substituents. Additionally, for the bulky substituent **5m**, we observed unique interactions with Asp234 and Asp210, respectively (Figure 3D).

The main difference between the binding of the **5f** molecule and the binding of the natural ligand 2′3′-*c*GAMP that we observed is the interaction of **5f** with Tyr240, as described earlier (Figure 3A). By modifying 7-deazaadenine with phenyl, we displaced Arg238 from chain B; we also brought additional intramolecular stabilization of the ligand conformation through cation−π stacking of the phenyl moiety with guanosine on the opposite side of the molecule. However, this interaction was not sufficient and resulted in lower stability of STING-**5f** in DSF compared to 2′3′-*c*GAMP and **5b**.

Intramolecular interactions were further strengthened in compound **5k**, where the phenyl ring was replaced by biphenylyl. Similar to **5f**, this led to a stacking interaction between Tyr240 and nucleobase. Additionally, Tyr240 was involved in a stacking interaction with the phenyl closer to the nucleobase in biphenylyl substituent (Figure 3B), which resulted in a 1.5-fold higher stability of STING-**5k** complex in DSF when compared to **5f**.

For **5l** with a 4-(2-naphthyl)phenyl substitution, we preserved the interactions that were observed for **5k** (Figure 3C); however, naphthyl substitution clashed with Ile235 of the lid, making this residue disordered (Figure S1). Despite this clash, **5l** exhibited a slightly better stabilization of complex in DSF when compared to **5k** (Table 1).

Compound **5m** showed additional π-stacking interactions with Tyr240 and an unexpected change in the binding mode of the substituent. The structural reason for this is a change in the position of the naphthyl group, that is, in **5m**, observed in the position regularly occupied by the Arg232 side chain. This position change is allowed due to the flexibility of the longer methoxy linker connecting the naphthyl group that enables the formation of a T-shaped π-stacking between the naphthyl of **5m** and Tyr240, π–amide interaction with the backbone of Asp231 and π–anion interaction with Asp210 (Figure 3D). A substitution at **5m** resulted in just a slightly lower stabilization in DSF when compared to 2′3′-*c*GAMP.

### PBMC Assay

Selected compounds were further tested for induction of IFNγ, TNFα, and IFNα secretion using PBMCs (Table 2). None of the tested compounds showed any cytotoxicity (Table S4). Most of the tested compounds induced higher levels of IFNγ, TNFα, and IFNα secretion when compared to 2′3′-*c*GAMP. EC_50_ obtained from testing at PBMCs correlated with data from a standard 293T cell-based reporter assay for most of the tested compounds.

**Table 2:** Cytokine Level Determination in PBMCs upon Treatment with Synthesized Compounds

	EC_50_ (μM)^a^		
compound	IFNγ	TNFα	IFNα
**5b**	7.8	50.60	30.60
**5c**	1.29	37.59	4.95
**5f**	6.84	52.45	7.36
**5g**	7.54	98.80	11.71
**5h**	34.51	>200	28.78
**5i**	85.20	>200	74.73
**5j**	47.54	>200	>200
**5k**	2.05	13.64	7.43
**5l**	1.77	53.86	5.09
**5m**	1.16	43.38	4.27
**5n**	2.24	6.72	2.80
**5o**	2.60	2.66	2.88
**5p**	8.33	24.56	12.84
**5q**	0.87	1.43	4.04
**5r**	5.63	99.70	26.98
**7f**	12.56	65.77	6.92
2′3′-*c*GAMP	7.10	36.03	8.46
3′3′-*c*GAMP	17.25	18.72	7.65
2′2′-*c*GAMP	11.75	44.54	7.21

a EC_50_ values are the mean of three independent experiments (*n* = 3), each of them performed on PBMCs from a different donor, measured in triplicate with SD < 50% of EC_50_ values.

## Conclusions

Here, we report the design, synthesis, and biochemical and biological evaluation of a novel class of 7-substituted 7-deazaadenine containing CDNs capable of STING activation. During the preparation of this class of compounds, we demonstrated a mixed enzymatic–chemical synthetic approach for the preparation of CDNs. This approach allowed us to efficiently overcome the total synthesis of the whole CDN, a multistep and time-consuming process.^53^ In the optimization process of biochemical binding potency of our compounds, SAR revealed an unexpected potential for modifications with large aromatic groups. This potential is applicable for 2′3′-*c*GAMP analogues with a preference for modifications that point above the CDN molecule. We observed π–π stacking interactions between the aromatic substituents and Tyr240 that are involved in stabilization of CDN–STING complexes. In the case of 3′3′-*c*GAMP and *c*-di-AMP, our modifications did not lead to increased binding potency. Substitutions with large aromatic moieties increased lipophilicity and thus potentially permeability into cells. They might also be more efficiently transported by an SLC19A1 receptor or an LRRC8A transporter, which would result in improved cellular activity.^54^

## Experimental Section

### General Synthetic and Enzymatic Methods

Unless otherwise noted, all starting materials, solvents, and reagents were purchased from commercial suppliers and used as received. 7-Iodo-7-adenosine **9** and 7-deazapurine NTPs **2b**,**f, 3a–d**,**f**,**n**, and **o** were prepared according to the literature.^37,44,45,55^ The synthesis of **3e** is described in the Supporting Information (Scheme S1). All chemical reactions were performed under an argon atmosphere. Reactions were monitored by thin layer chromatography (TLC) on TLC Silica gel 60 F_254_ (Merck) and detected by UV (254 nm). NMR spectra were measured on a Bruker AVANCE 500 MHz spectrometer (499.8 MHz for ^1^H, 125.7 MHz for ^13^C, and ^31^P at 202.4 MHz) or on a Bruker 600 AVANCE III HD instrument (^1^H at 600 MHz, ^13^C at 150.9 MHz) equipped with 5 mm cryo-probe at 25 °C in D_2_O (dioxane used as external standard, [δ(^1^H) = 3.75 ppm, δ(^13^C) = 67.19 ppm]) or in CD_3_OD (referenced to the residual solvent signal, [δ(^1^H) = 3.31 ppm, δ(^13^C) = 49.0 ppm]). ^31^P NMR spectra were referenced externally to the signal of H_3_PO_4_. Chemical shifts are given in ppm (δ-scale), and coupling constants (*J*) are given in Hz. The complete assignment of all NMR signals was performed using a combination of H,H-COSY, H,H-ROESY, H,C-HSQC, and H,C-HMBC experiments. Low resolution mass spectra were measured using electrospray ionization (ESI). High resolution mass spectra were measured on LTQ Orbitrap XL (Thermo Fisher Scientific) using ESI. High performance flash chromatography (HPFC) was performed with ISCO Combiflash Rf system on RediSep Rf Gold Silica Gel columns or Reverse Phase (C18) RediSep Rf Gold columns. Purification of CDNs was performed using HPLC (Waters modular HPLC system) on a column packed with 5 μm polar C18 reversed phase (Luna Omega 5 μm Polar C18 column, Phenomenex). The purity of all final compounds was determined by clean NMR spectra and by UPLC. The identification of CDNs was performed on ACQUITY UPLC HClass PLUS chromatographic system with MS SQ Detector 2 (Waters, Milford, USA) using Acquity UPLC BEH C18 column 50 mm × 2.1 mm, 1.7 μm (Waters, Milford, MA, USA), and 20 mM ammonium acetate buffer, pH 6.8, with a linear gradient of acetonitrile (0 to 50% in 4 min, additional 2 min at 50% acetonitrile; flow rate 0.5 mL/min). Column temperature was kept at 40 °C. Negative ESI method was used for ionization. ULPC purity (>95% with exception of enzymatically synthesized CDNs **4b**, **5b,** and **5e**) is shown in Table S3.

#### Enzymatic Synthesis

Enzymatically prepared 2′3′-CDNs (Schemes 3 and 4) were synthetized using mcGAS as described previously.^20^ Briefly, the appropriate NTPs were mixed to the final 2 mM concentration with 5 μM of mcGAS and 0.1 mg/mL herring testes DNA in 1 mL buffer containing 20 mM Tris–HCl [pH 8.0] and 20 mM MgCl_2_ and incubated for 16 h at 37 °C in a heating shaker. The synthesis of **7a** was performed by using 2 mM **3a**, 2 mM GTP, and 2 μM DncV in 1 mL of 50 mM HEPES buffer [pH 8.0] supplemented with 100 mM NaCl, 10 mM MgCl_2_, and 1 mM DTT. The whole mixture was incubated overnight at 37 °C in a heating shaker. The synthesis of **8a** was done overnight at 50 °C in a 1 mL reaction mixture containing 4 mM **3a**, 20 μM DisA, and 50 mM HEPES buffer [pH 8.0] supplemented with 100 mM NaCl, 10 mM MgCl_2_, and 1 mM DTT. The next morning, the reactions were spun at 25,000*g* for 15 min and supernatants were passed through Nanosep 3K Omega (Pall Corporation, USA). The purification of CDNs was continued by adding 3 mL of ddH_2_O to the flow-through fractions, and CDNs were purified on a semipreparative C18 column (Luna 5 μm C18 250 mm × 10 mm) using a 60 min gradient at a flow rate of 3 mL/min of 0–20% acetonitrile in 0.1 M TEAB buffer [pH 8.5]. TEAB was removed from the collected fractions by 3 cycles of evaporation/dissolution in 50% methanol.

##### Cyclo-adenosine 5′-*O*-Phosphate (3′ → 5′) 2-Amino-5-methyl-7-β-d-ribofuranosyl-7*H*-pyrrolo[2,3-*d*]pyrimidin-4(3*H*)-one 5′-*O*-Phosphate (2′ → 5′) Sodium Salt (**4b**)

NTP **2b** (2 μmol) and ATP (2 μmol) were enzymatically cyclized using mcGAS. The final product was purified by HPLC (5–30% MeCN in 0.1 M TEAB). The conversion to a sodium salt form on a Dowex 50WX8 (in a Na^+^ cycle) provided CDN **4b** (73 nmol, 4%). ^1^H NMR (600 MHz, D_2_O): 1.81 (bs, 3H, CH_3_); 4.14 (ddd, 1H, *J*_5′b,4′_ = 1.7, *J*_5′b,5′a_ = 11.8, *J*_5′b,P_ = 3.4, H5′b-G); 4.23 (ddd, 1H, *J*_5′a,4′_ = 2.3, *J*_5′a,5′b_ = 11.8, *J*_5′a,P_ = 2.7, H5′a-G); 4.23 (ddd, 1H, *J*_5′b,4′_ = 1.4, *J*_5′b,5′a_ = 12.1, *J*_5′b,P_ = 3.6, H5′b-A); 4.37 (ddd, 1H, *J*_4′,5′a_ = 2.3, *J*_4′,5′b_ = 1.7, *J*_4′,P_ = 3.8, H4′-G); 4.47 (m, 1H, H5′a-A); 4.49 (dm, 1H, *J*_4′,3′_ = 8.7, H4′-A); 4.62 (d, 1H, *J*_3′,4′_ = 4.1, H3′-G); 4.75 (dd, *J*_2′,1′_ = 1.1, *J*_2′,3′_ = 4.3, H2′-A); 4.99 (ddd, 1H, *J*_3′,2′_ = 4.3, *J*_3′,4′_ = 8.7, *J*_3′,P_ = 6.9, H3′-A); 5.33 (um, 1H, H2′-G); 6.01 (d, 1H, *J*_1′,2′_ = 8.3, H1′-G); 6.19 (d, 1H, *J*_1′,2′_ = 1.1, H1′-A); 6.83 (q, 1H, *J*_8,CH_3__ = 1.3, H8-G); 8.23 (s, 1H, H8-A); 8.27 (s, 1H, H2-A). ^13^C NMR (150.9 MHz, D_2_O): 12.90 (5-CH_3_); 65.61 (C5′-A); 68.75 (C5′-G); 73.55 (C3′-A); 74.20 (C3′-G); 76.54 (C2′-A); 79.46 (C2′-G); 83.02 (C4′-A); 85.95 (C4′-G); 87.70 (C1′-G); 92.47 (C1′-A); 103.55 (C5-G); 118.24 (C4a-G); 119.52 (C6-G); 121.67 (C5-A); 141.42 (C8-A); 150.64 (C4-A); 154.81 (C7a-G); 155.07 (C2-G); 155.40 (C2-A); 158.26 (C6-A); ^31^P NMR (^1^H-dec, 202.4 MHz, D_2_O): −0.20 and −1.02. ESI MS *m*/*z* (rel. %): 342 (100) [M–2H]^2–^, 686 (37) [M–H]^−^, 708 (8) [M–2H + Na]^−^. HR MS (ESI): for C_22_H_25_N_9_O_13_P_2_ [M–H]^−^, calcd 686.11308; found, 686.11212.

##### Cyclo-4-amino-5-iodo-7-β-d-ribofuranosyl-7*H*-pyrrolo[2,3-*d*]pyrimidine 5′-*O*-Phosphate (3′ → 5′) Guanosine 5′-*O*-Phosphate (2′ → 5′) Sodium Salt (**5a**)

Enzymatic synthesis: NTP **3a** (75 mg, 107 μmol) and GTP (107 μmol) were enzymatically cyclized using mcGAS. The final product was purified by HPLC (5–30% MeCN in 0.1 M TEAB). The conversion to a sodium salt form on a Dowex 50WX8 (in a Na^+^ cycle) provided CDN **5a** (56 mg, 61%) as a white lyophilizate (water).

Chemical synthesis: Phosphonate **12** (161 mg, 0.21 mmol) was dissolved in DCM (2.5 mL). Water (38 μL, 2.11 mmol) and dichloroacetic acid in DCM (6%, 2.5 mL, 1.81 mmol) were added, and the solution was stirred for 10 min. Then, pyridine (298 μL, 3.70 mmol) was added, and the mixture was evaporated under reduced pressure and co-evaporated with anhydrous acetonitrile (3×). The residue was dissolved in anhydrous acetonitrile (480 μL), and a solution of phosphoramidite **13** (262 mg, 0.268 mmol) in anhydrous acetonitrile (1.45 mL) was added. The mixture was stirred at rt for 10 min, then *t*-butylhydroperoxide (5.5 M solution in decane, 113 μL, 0.621 mmol) was added, and the stirring continued for another 30 min. Then, the solution was cooled to 0 °C, and a solution of NaHSO_3_ (33% wt, 627 μL, 2.46 mmol) was added. The mixture was stirred for 10 min at 0 °C and then for 5 min at rt. Then, solvent was removed in vacuo, and the residue was dissolved in DCM (3.22 mL). Water (38 μL, 2.11 mmol) and dichloroacetic acid in DCM (6%, 3.22 mL, 2.33 mmol) were added. The mixture was stirred for 10 min, then pyridine (670 μL, 8.32 mmol) was added, and the mixture was evaporated under reduced pressure and co-evaporated with water. Product **14** was partially purified by flash chromatography (C18 column, gradient 5–100% MeCN in 0.1 M TEAB).

Crude **14** was co-evaporated with anhydrous pyridine (3 × 2 mL) and dissolved in anhydrous pyridine (2.6 mL). DMOCP was added (85 mg, 0.46 mmol), and the mixture was stirred for 110 min at rt. Then, water (77 μL, 0.46 mmol) and iodine (44 mg, 0.46 mmol) were added, and the stirring continued for another 10 min at rt. The mixture was cooled to 0 °C, and an aqueous solution of NaHSO_3_ (40% wt, 64 μL) was added. After stirring for 5 min at 0 °C, another portion of NaHSO_3_ solution (40% wt, 40 μL) was added. The clear solution was evaporated under reduced pressure and co-evaporated with water. Crude **15** was partially purified by flash chromatography (C18 column, gradient 5–100% MeCN in 0.1 M TEAB).

Crude **15** was dissolved in ethanolic solution of CH_3_NH_2_ (33% wt., 4 mL, 32.1 mmol), and the solution was stirred for 3 h at rt. Then, the mixture was evaporated under reduced pressure, and the residue was co-evaporated with anhydrous pyridine (3 × 2 mL). A mixture of anhydrous pyridine, Et_3_N (1:1 v/v, 4 mL), and Et_3_N·3HF (640 μL, 3.93 mmol) was added, and the mixture was stirred at 50 °C for 3.5 h. Then, aqueous ammonium acetate (1 M, 6 mL) was added, and the solvents were removed in vacuo. The residue was co-evaporated with water. After HPLC purification (0–15% MeCN in 0.1 M TEAB) and conversion to a sodium salt form on a Dowex 50WX8 (in a Na^+^ cycle), CDN **5a** (32 mg, 18% overall yield) was obtained as a white lyophilizate (water). ^1^H NMR (600 MHz, D_2_O): 4.14 (ddd, 1H, *J*_5′b,4′_ = 1.8, *J*_5′b,5′a_ = 11.7, *J*_5′b,P_ = 2.1, H5′b-G); 4.22 (ddd, 1H, *J*_5′a,4′_ = 3.1, *J*_5′a,5′b_ = 11.7, *J*_5′a,P_ = 4.6, H5′a-G); 4.23 (ddd, 1H, *J*_5′b,5′a_ = 12.0, *J*_5′b,4′_ = 1.3, *J*_5′b,P_ = 2.2, H5′b-A); 4.41 (ddd, 1H, *J*_4′,5′a_ = 3.1, *J*_4′,5′b_ = 1.8, *J*_4′,P_ = 3.6, H4′-G); 4.44 (dm, 1H, *J*_4′,3′_ = 9.3, H4′-A); 4.52 (dm, 1H, *J*_5′a,5′b_ = 12.0, H5′a-A); 4.61 (d, 1H, *J*_3′,2′_ = 4.0, H3′-G); 4.67 (bd, 1H, *J*_2′,3′_ = 4.0, H2′-A); 4.92 (ddd, 1H, *J*_3′,2′_ = 4.0, *J*_3′,4′_ = 9.3, *J*_3′,P_ = 6.6, H3′-A); 5.62 (ddd, 1H, *J*_2′,1′_ = 8.6, *J*_2′,3′_ = 4.0, *J*_2′,P_ = 4.4, H2′-G); 5.94 (d, 1H, *J*_1′,2′_ = 8.6, H1′-G); 6.10 (s, 1H, H1′-A); 7.71 (s, 1H, H6-A); 7.88 (s, 1H, H8-G); 8.09 (s, 1H, H2-A). ^13^C NMR (150.9 MHz, D_2_O): 51.15 (C5-A); 65.23 (d, *J*_C,P_ = 4.5, C5′-A); 68.48 (d, *J*_C,P_ = 5.2, C5′-G); 72.88 (d, *J*_C,P_ = 5.6, C3′-A); 73.75 (C3′-G); 76.68 (C2′-A); 77.43 (d, *J*_C,P_ = 5.4, C2′-G); 82.55 (t, *J*_C,P1_ = *J*_C,P2_ = 11.3, C4′-A); 85.14 (d, *J*_C,P_ = 10.1, C4′-G); 89.10 (d, *J*_C,P_ = 12.0, C1′-G); 92.50 (C1′-A); 106.80 (C4a-A); 120.60 (C5-G); 129.42 (C6-A); 143.38 (C8-G); 150.09 (C7a-A); 153.95 (C2-A); 154.71 (C4-G); 155.84 (C2-G); 159.59 (C4-A); 161.74 (C6-G). ^31^P NMR (^1^H-dec, 202.4 MHz, D_2_O): −0.32 and −1.41. ESI MS *m*/*z* (rel. %): 398 (100) [M–2H]^2–^, 798 (97) [M–H]^−^, 820 (30) [M–2H + Na]^−^. HR MS (ESI): for C_21_H_23_O_13_N_9_IP_2_ [M–H]^−^, calcd 797.99407; found, 797.99335.

##### Cyclo-4-amino-5-methyl-7-β-d-ribofuranosyl-7*H*-pyrrolo[2,3-*d*]pyrimidine 5′-*O*-Phosphate (3′ → 5′) Guanosine 5′-*O*-Phosphate (2′ → 5′) Sodium Salt (**5b**)

NTP **3b** (2 μmol) and GTP (2 μmol) were enzymatically cyclized using mcGAS. The final product was purified by HPLC (5–30% MeCN in 0.1 M TEAB). The conversion to a sodium salt form on a Dowex 50WX8 (in a Na^+^ cycle) provided CDN **5b** (354 nmol, 18%). ^1^H NMR (600 MHz, D_2_O): 1.98 (d, 3H, *J*_CH3,6_ = 1.1, CH_3_); 4.17 (m, 1H, H5′b-G); 4.18 (ddd, 1H, *J*_5′b,5′a_ = 11.7, *J*_5′b,4′_ = 2.4, *J*_5′b,P_ = 1.8, H5′b-A); 4.26 (ddd, 1H, *J*_5′a,5′b_ = 11.7, *J*_5′a,4′_ = 3.2, *J*_3′P_ = 4.7, H5′a-A); 4.43 (m, 2H, H4′-A and H4′-G); 4.44 (m, 1H, H5′a-G); 4.63 (d, 1H, *J*_3′,4′_ = 4.1, H3′-G); 4.71 (bd, 1H, *J*_2′,3′_ = 4.3, H2′-A); 5.06 (ddd, 1H, *J*_3′,2′_ = 4.3, *J*_3′,4′_ = 9.0, *J*_3′,P_ = 6.7, H3′-A); 5.62 (ddd, 1H, *J*_2′,1′_ = 8.5, *J*_2′,3′_ = 4.1, *J*_2′,P_ = 6.6, H2′-G); 5.96 (d, 1H, *J*_1′,2′_ = 8.5, H1′-G); 6.23 (s, 1H, H1′-A); 7.36 (q, 1H, *J*_6,CH3_ = 1.1, H6-A); 7.92 (s, 1H, H8-G); 8.19 (bs, 1H, H2-A). ^13^C NMR (150.9 MHz, D_2_O; chemical shifts obtained from 2D-HSQC and 2D-HMBC spectra; *J*(C,P) and shifts of C4-A, C2-G, and C6-G were not determined): 13.29 (CH_3_); 64.98 (C5′-A); 68.33 (C5′-G); 73.32 (C3′-A); 73.85 (C3′-G); 77.08 (C2′-A); 77.49 (C2′-G); 82.64 (C4′-A); 86.11 (C4′-G); 88.85 (C1′-G); 92.20 (C1′-A); 105.17 (C4a-A); 114.97 (C5-A); 119.83 (C5-G); 123.61 (C6-A); 143.19 (C8-G); 147.84 (C2-A); 149.48 (C7a-A); 154.89 (C4-G). ^31^P NMR (^1^H-dec, 202.4 MHz, D_2_O): −0.27 and −1.18. ESI MS *m*/*z* (rel. %): 342 (100) [M–2H]^2–^, 353 (3) [M–3H + Na]^2–^, 686 (36) [M–H]^−^, 708 (8) [M–2H + Na]^−^. HR MS (ESI): for C_22_H_27_N_9_O_13_P_2_, calcd 686.11308; found, 686.11212.

##### Cyclo-4-amino-5-cyclopropyl-7-β-d-ribofuranosyl-7*H*-pyrrolo[2,3-*d*]pyrimidine 5′-*O*-Phosphate (3′ → 5′) Guanosine 5′-*O*-Phosphate (2′ → 5′) Sodium Salt (**5c**)

NTP **3c** (2 μmol) and GTP (2 μmol) were enzymatically cyclized using mcGAS. The final product was purified by HPLC (5–30% MeCN in 0.1 M TEAB). The conversion to a sodium salt form on a Dowex 50WX8 (in a Na^+^ cycle) provided CDN **5c** (387 nmol, 19%). ^1^H NMR (600 MHz, D_2_O): 0.16 m and 0.74 m (2H, H3-cyclopropyl); 0.31 m and 0.47 m (2H, H2-cyclopropyl); 1.52 (m, 1H, H1-cyclopropyl); 4.18 (dt, 1H, *J*_5′b,4′_ = 1.8, *J*_5′b,5′a_ = 11.7, *J*_5′b,P_ = 1.8, H5′b-G); 4.24 (ddd, 1H, *J*_5′b,5′a_ = 11.8, *J*_5′b,4′_ = 2.6, *J*_5′b,P_ ∼ 1.0, H5′b-A); 4.26 (ddd, 1H, *J*_5′a,4′_ = 3.2, *J*_5′a,5′b_ = 11.7, *J*_5′a,P_ = 4.3, H5′a-G); 4.41 (dm, 1H, *J*_4′,3′_ = 9.2, H4′-A); 4.44 (m, 1H, *J*_5′a,5′b_ = 11.8, H5′a-A); 4.45 (m, 1H, H4′-G); 4.66 (d, 1H, *J*_3′,4′_ = 4.0, H3′-G); 4.70 (d, 1H, *J*_2′,3′_ = 4.2, H2′-A); 5.05 (ddd, 1H, *J*_3′,2′_ = 4.2, *J*_3′,4′_ = 9.2, *J*_3′,P_ = 6.7, H3′-A); 5.65 (ddd, 1H, *J*_2′,1′_ = 8.6, *J*_2′,3′_ = 4.0, *J*_2′,P_ = 4.8, H2′-G); 5.98 (d, 1H, *J*_1′,2′_ = 8.6, H1′-G); 6.22 (s, 1H, H1′-A); 7.10 (s, 1H, H6-A); 7.96 (bs, 1H, H8-G); 8.18 (bs, 1H, H2-A). ^13^C NMR (150.9 MHz, D_2_O, chemical shifts of quaternary carbons C2-G, C4-G, C5-G, C6-G, and C2-A were not determined): 6.49 (C2-cyclopropyl); 8.97 (C1-cyclopropyl); 10.80 (C3-cyclopropyl); 65.31 (d, *J*_C,P_ = 5.0, C5′-A); 68.48 (d, *J*_C,P_ = 4.7, C5′-G); 73.09 (d, *J*_C,P_ = 5.6, C3′-A); 73.66 (C3′-G); 76.99 (C2′-A); 77.63 (d, *J*_C,P_ = 5.7, C2′-G); 82.42 (dd, *J*_C,P1_ = 11.3, *J*_C,P2_ = 10.5, C4′-A); 86.08 (d, *J*_C,P_ = 10.2, C4′-G); 89.02 (d, *J*_C,P_ = 12.7, C1′-G); 91.94 (C1′-A); 105.76 (C5-A); 121.01 (C6-A); 121.77 (C4a-A); 143.31 (C8-G); 149.79 (C7a-A); 161.15 (C4-A). ^31^P NMR (^1^H-dec, 202.4 MHz, D_2_O): −0.26 and −1.09. ESI MS *m*/*z* (rel. %): 356 (100) [M–2H]^2–^, 367 (6) [M–3H + Na]^2–^, 712 (7) [M–H]^−^, 734 (8) [M–2H + Na]^−^. HR MS (ESI): for C_24_H_28_O_13_N_9_P_2_, calcd 712.12873; found, 712.12814.

##### Cyclo-4-amino-5-ethynyl-7-β-d-ribofuranosyl-7*H*-pyrrolo[2,3-*d*]pyrimidine 5′-*O*-Phosphate (3′ → 5′) Guanosine 5′-*O*-Phosphate (2′ → 5′) Sodium Salt (**5d**)

NTP **3d** (2 μmol) and GTP (2 μmol) were enzymatically cyclized using mcGAS. The final product was purified by HPLC (5–30% MeCN in 0.1 M TEAB). The conversion to a sodium salt form on a Dowex 50WX8 (in a Na^+^ cycle) provided CDN **5d** (196 nmol, 10%). ^1^H NMR (600 MHz, D_2_O): 3.41 (s, 1H, −C≡CH); 4.17 (dt, 1H, *J*_5′b,4′_ = 2.0, *J*_5′b,5′a_ = 11.8, *J*_5′b,P_ = 2.2, H5′b-G); 4.22 (bdd, 1H, *J*_5′b,4′_ = 2.5, *J*_5′b,5′a_ = 12.0, H5′b-A); 4.24 (ddd, 1H, *J*_5′a,4′_ = 3.1, *J*_5′a,5′b_ = 11.8, *J*_5′a,P_ = 4.6, H5′a-G); 4.42 (dt, 1H, *J*_4′,5′a_ = 3.1, *J*_4′,5′b_ = 2.0, *J*_4′,P_ = 3.1, H4′-G); 4.46 (bdt, 1H, *J*_4′,3′_ = 9.4, *J*_4′,5′a_ = 2.5, *J*_4′,5′b_ = 2.5, H4′-A); 4.50 (dm, 1H, *J*_5′a,5′b_ = 12.0, H5′a-A); 4.63 (d, 1H, *J*_3′,4′_ = 4.1, H3′-G); 4.66 (bd, 1H, *J*_2′,3′_ = 4.0, H2′-A); 4.99 (ddd, 1H, *J*_3′,2′_ = 4.0, *J*_3′,4′_ = 9.4, *J*_3′,P_ = 6.6, H3′-A); 5.69 (ddd, 1H, *J*_2′,1′_ = 8.5, *J*_2′,3′_ = 4.1, *J*_2′,P_ = 5.7, H2′-G); 5.96 (d, 1H, *J*_1′,2′_ = 8.5, H1′-G); 6.26 (s, 1H, H1′-A); 7.86 (s, 1H, H6-A); 7.91 (s, 1H, H8-G); 8.23 (s, 1H, H2-A). ^13^C NMR (150.9 MHz, D_2_O; chemical shifts obtained from 2D-HSQC and 2D-HMBC spectra; *J*(C,P) and shifts of C2-G and C6-G not determined): 61.36 (-C≡**C**H); 65.02 (C5′-A); 68.40 (C5′-G); 72.82 (C3′-A); 73.82 (C3′-G); 76.91 (C2′-A); 77.38 (C2′-G); 82.47 (C4′-A); 84.18 (-**C**≡CH); 85.94 (C4′-G); 88.89 (C1′-G); 92.31 (C1′-A); 97.30 (C5-A); 105.67 (C4a-A); 120.22 (C5-G); 130.16 (C6-A); 143.10 (C8-G); 149.06 (C7a-A); 154.62 (C4-G); 151.83 (C2-A); 157.90 (C4-A). ^31^P NMR (^1^H-dec, 202.4 MHz, D_2_O): −0.18 and −1.14. ESI MS *m*/*z* (rel. %): 348 (100) [M–2H]^2–^, 696 (21) [M–H]^−^, 718 (16) [M–2H + Na]^−^. HR MS (ESI): for C_23_H_23_O_13_N_9_P_2_, calcd 696.09743; found, 696.09637.

##### Cyclo-4-amino-5-(pent-1-yn-1-yl)-7-β-d-ribofuranosyl-7*H*-pyrrolo[2,3-*d*]pyrimidine 5′-*O*-Phosphate (3′ → 5′) Guanosine 5′-*O*-Phosphate (2′ → 5′) Sodium Salt (**5e**)

NTP **3e** (2 μmol) and GTP (2 μmol) were enzymatically cyclized using mcGAS. The final product was purified by HPLC (5–30% MeCN in 0.1 M TEAB). The conversion to a sodium salt form on a Dowex 50WX8 (in a Na^+^ cycle) provided CDN **5e** (84 nmol, 4%). ^1^H NMR (600 MHz, D_2_O): 0.91 (t, 3H, H5-pentynyl); 1.47 (m, 2H, H4-pentynyl); 2.29 (m, 2H, H3-pentynyl); 4.15 (ddd, 1H, *J*_5′b,4′_ = 2.0, *J*_5′b,5′a_ = 11.9, *J*_5′b,P_ = 2.0, H5′b-G); 4.23 (ddd, 1H, *J*_5′a,4′_ = 3.0, *J*_5′a,5′b_ = 11.9, *J*_5′a,P_ = 4.7, H5′a-G); 4.25 (dm, 1H, *J*_5′b,5′a_ = 12.0, H5′b-A); 4.41 (td, 1H, *J*_4′,5′a_ = 3.0, *J*_4′,5′b_ = 2.0, *J*_4′,P_ = 3.2, H4′-G); 4.45 (dm, 1H, *J*_4′,3′_ = 9.5, H4′-A); 4.51 (dm, 1H, *J*_5′a,5′b_ = 12.0, H5′a-A); 4.62 (d, 1H, *J*_3′,4′_ = 4.3, H3′-G); 4.63 (d, 1H, *J*_2′,3′_ = 4.1, H2′-A); 4.98 (ddd, 1H, *J*_3′,2′_ = 4.1, *J*_3′,4′_ = 9.5, *J*_3′,P_ = 6.6, H3′-A); 5.75 (dt, 1H, *J*_2′,1′_ = 8.6, *J*_2′,3′_ = 4.3, *J*_2′,P_ = 4.3, H2′-G); 5.95 (d, 1H, *J*_1′,2′_ = 8.6, H1′-G); 6.22 (s, 1H, H1′-A); 7.67 (s, 1H, H6-A); 7.88 (bs, 1H, H8-G); 8.18 (bs, 1H, H2-A). ^13^C NMR (150.9 MHz, D_2_O; chemical shifts obtained from 2D-HSQC and 2D-HMBC spectra; *J*(C,P) and shifts of C2-G, C4-G, C5-G, and C6-G not determined): 15.55 (C5-pentynyl); 23.44 (C3-pentynyl); 24.04 (C4-pentynyl); 65.09 (C5′-A); 68.48 (C5′-G); 72.73 (C3′-A); 73.80 (C3′-G); 74.50 (C1-pentynyl); 76.83 (C2′-A); 77.11 (C2′-G); 82.28 (C4′-A); 85.73 (C4′-G); 89.00 (C1′-G); 92.02 (C1′-A); 96.60 (C2-pentynyl); 98.09 (C5-A); 105.93 (C4a-A); 127.64 (C6-A); 143.12 (C8-G); 149.52 (C7a-A); 154.41 (C2-A); 159.83 (C4-A); ^31^P NMR (^1^H-dec, 202.4 MHz, D_2_O): −0.15 and −0.83. ESI MS *m*/*z* (rel. %): 368 (100) [M–2H]^2–^, 738 (15) [M–H]^−^, 760 (9) [M–2H + Na]^−^. HR MS (ESI): for C_26_H_30_O_13_N_9_P_2_, calcd 738.14438; found, 738.14313.

##### Cyclo-4-amino-5-phenyl-7-β-d-ribofuranosyl-7*H*-pyrrolo[2,3-*d*]pyrimidine 5′-*O*-Phosphate (3′ → 5′) Guanosine 5′-*O*-Phosphate (2′ → 5′) Sodium Salt (**5f**)

Enzymatic synthesis: NTP **3f** (2 μmol) and GTP (2 μmol) were enzymatically cyclized using mcGAS. The final product was purified by HPLC (5–30% MeCN in 0.1 M TEAB). The conversion to a sodium salt form on a Dowex 50WX8 (in a Na^+^ cycle) provided CDN **5f** (100 nmol, 5%).

Chemical synthesis: CDN **5a** (15 mg, 17.8 μmol), phenylboronic acid (4.3 mg, 35.6 μmol), and cesium carbonate (17.4 mg, 53.4 μmol) were mixed with MeCN–H_2_O (1:2 v/v, 600 μL) in an argon purged vial. In a separate vial, Pd(OAc)_2_ (1.0 mg, 4.45 μmol) and TPPTS (12.6 mg, 22.2 μmol) were dissolved in MeCN–H_2_O (1:2 v/v, 1.0 mL), and the solution was sonicated under an argon atmosphere for 30 s. Then, 200 μmol (i.e., 1/5) of this solution was transferred into the mixture containing the CDN **5a**, and the reaction was stirred at 100 °C for 30 min. Then, the reaction mixture was cooled to rt, diluted with water to approx. 3 mL, and filtered through a 5 μm nylon syringe filter. The filtrate was directly applied on HPLC for purification. After two HPLC separations (0–15% MeCN in 0.1 M TEAB) and conversion to a sodium salt form on a Dowex 50WX8 (in a Na^+^ cycle), CDN **5f** (2.5 mg, 18%) was obtained as a white lyophilizate (water). ^1^H NMR (600 MHz, D_2_O): 4.15 (ddd, 1H, *J*_5′b,4′_ = 1.8, *J*_5′b,5′a_ = 11.8, *J*_5′b,P_ = 2.5, H5′b-G); 4.25 (ddd, 1H, *J*_5′a,4′_ = 2.8, *J*_5′a,5′b_ = 11.8, *J*_5′a,P_ = 5.0, H5′a-G); 4.29 (m, 1H, H5′b-A); 4.43 (ddd, 1H, *J*_4′,5′a_ = 2.8, *J*_4′,5′b_ = 1.8, *J*_4′,P_ = 3.6, H4′-G); 4.49 (dm, 1H, *J*_4′,3′_ = 9.1, H4′-A); 4.51 (m, 1H, H5′a-A); 4.66 (d, 1H, *J*_3′,2′_ = 4.1, H3′-G); 4.79 (bd, 1H, *J*_2′,3′_ = 4.1, H2′-A); 5.02 (ddd, 1H, *J*_3′,2′_ = 4.1, *J*_3′,4′_ = 9.1, *J*_3′,P_ = 6.9, H3′-A); 5.73 (dt, 1H, *J*_2′,1′_ = 8.6, *J*_2′,3′_ = 4.1, *J*_2′,P_ = 4.1, H2′-G); 6.01 (d, 1H, *J*_1′,2′_ = 8.6, H1′-G); 6.29 (s, 1H, H1′-A); 7.09 (m, 2H, *o*-Ph-A); 7.24 (m, 2H, *m*-Ph-A); 7.25 (m, 1H, *p*-Ph); 7.63 (s, 1H, H6-A); 7.89 (s, 1H, H8-G); 8.22 (s, 1H, H2-A). ^13^C NMR (150.9 MHz, D_2_O): 65.53 (d, *J*_C,P_ = 4.6, C5′-A); 68.66 (d, *J*_C,P_ = 5.4, C5′-G); 73.07 (d, *J*_C,P_ = 5.5, C3′-A); 73.93 (C3′-G); 76.96 (C2′-A); 77.87 (d, *J*_C,P_ = 4.6, C2′-G); 82.61 (t, *J*_C,P_ = 11.3, C4′-A); 86.04 (d, *J*_C,P_ = 9.7, C4′-G); 88.70 (d, *J*_C,P_ = 12.5, C1′-G); 92.27 (C1′-A); 103.79 (C4a-A); 119.40 (C5-A); 119.88 (C5-G); 122.74 (C6-A); 129.47 (*p*-Ph-A); 130.27 (o-Ph-A); 131.61 (*m*-Ph-A); 135.65 (*i*-Ph-A); 142.42 (C8-G); 151.35 (C7a-A); 153.88 (C2-A); 154.58 (C4-G); 155.99 (C2-G); 160.08 (C4-A); 161.17 (C6-G). ^31^P NMR (^1^H-dec, 202.4 MHz, D_2_O): −0.19 and −0.90. ESI MS *m*/*z* (rel. %): 373 (100) [M–2H]^2–^, 385 (5) [M–3H + Na]^2–^, 748 (2) [M–H]^−^, 770 (6) [M–2H + Na]^−^. HR MS (ESI): for C_27_H_28_O_13_N_9_P_2_ [M–H]^−^, calcd 748.12873; found, 748.12862.

##### Cyclo-4-amino-5-(naphthalen-2-yl)-7-β-d-ribofuranosyl-7*H*-pyrrolo[2,3-*d*]pyrimidine 5′-*O*-phosphate (3′ → 5′) Guanosine 5′-*O*-phosphate (2′ → 5′) Sodium Salt (**5g**)

CDN **5a** (13 mg, 15.4 μmol), naphthalene-2-boronic acid (13.3 mg, 77.1 μmol), and cesium carbonate (15 mg, 46.2 μmol) were mixed with MeCN–H_2_O (1:2 v/v, 520 μL) in an argon purged vial. In a separate vial, Pd(OAc)_2_ (1.0 mg, 4.45 μmol) and TPPTS (12.6 mg, 22.2 μmol) were dissolved in MeCN–H_2_O (1:2v/v, 1.0 mL), and the solution was sonicated under argon atmosphere for 30 s. Then, 173 μmol of this solution was transferred into the mixture containing the CDN **5a**, and the reaction mixture was stirred at 100 °C for 30 min. Then, the reaction mixture was cooled to rt, diluted with water to approx. 3 mL, and filtered through a 5 μm nylon syringe filter. The filtrate was directly applied on HPLC for purification (5–25% MeCN in 0.1 M TEAB). After HPLC repurification (9–24% MeCN in 0.1 M TEAB) and conversion to a sodium salt form on a Dowex 50WX8 (in a Na^+^ cycle), CDN **5g** (5.7 mg, 44%) was obtained as a white lyophilizate (water). ^1^H NMR (600 MHz, D_2_O): 4.13 (ddd, 1H, *J*_5′b,4′_ = 1.6, *J*_5′b,5′a_ = 11.8, *J*_5′b,P_ = 2.6, H5′b-G); 4.26 (ddd, 1H, *J*_5′a,4′_ = 2.7, *J*_5′a,5′b_ = 11.8, *J*_5′a,P_ = 4.5, H5′a-G); 4.36 (ddd, 1H, *J*_5′b,4′_ = 1.3, *J*_5′b,5′a_ = 12.1, *J*_5′b,P_ = 2.9, H5′b-A); 4.45 (ddd, 1H, *J*_4′,5′a_ = 2.7, *J*_4′,5′b_ = 1.6, *J*_4′,P_ = 3.8, H4′-G); 4.53 (dm, 1H, *J*_4′,3′_ = 9.6, H4′-A); 4.57 (ddd, 1H, *J*_5′a,4′_ = 2.3, *J*_5′a,5′b_ = 12.1, *J*_5′a,P_ = 1.1, H5′a-A); 4.73 (d, 1H, *J*_3′,4′_ = 4.0, H3′-G); 4.81 (d, *J*_3′,2′_ = 3.9, H2′-A); 5.03 (ddd, 1H, *J*_3′,2′_ = 3.9, *J*_3′,4′_ = 9.6, *J*_3′,P_ = 6.6, H3′-A); 5.67 (um, 1H, H2′-G); 6.07 (d, 1H, *J*_1′,2′_ = 8.6, H1′-G); 6.34 (s, 1H, H1′-A); 7.32 (dd, 1H, *J*_3,2_ = 1.8, *J*_3,4_ = 8.3, H3-naphth); 7.53 (m, 1H, H7-naphth); 7.545 (m, 1H, H6-naphth); 7.64 (dm, 1H, *J*_1,3_ = 1.8, H1-naphth); 7.74 (s, 1H, H8-G); 7.75 (s, 1H, H6-A); 7.77 (dm, 1H, *J*_4,3_ = 8.3, H4-naphth); 7.82 (m, 1H, H5-naphth); 7.90 (m, 1H, H8-naphth); 8.25 (s, 1H, H2-A). ^13^C NMR (150.9 MHz, D_2_O): 65.66 (d, *J*_C,P_ = 4.7, C5′-A); 68.76 (d, *J*_C,P_ = 5.2, C5′-G); 73.16 (d, *J*_C,P_ = 5.4, C3′-A); 73.91 (C3′-G); 76.98 (C2′-A); 79.00 (C2′-G); 82.58 (t, *J*_C,P_ = 11.6, C4′-A); 86.13 (d, *J*_C,P_ = 9.6, C4′-G); 87.80 (C1′-G); 92.32 (C1′-A); 103.93 (C5-A); 119.40 (C4a-A); 119.61 (C5-G); 123.09 (C6-A); 128.19 (C1-naphth); 128.51 (C3-naphth); 128.70 (C7-naphth); 129.24 (C6-naphth); 130.26 (C8-naphth); 130.89 (C5-naphth); 131.18 (C4-naphth); 133.19 (C2-naphth); 134.43 (C4a-naphth); 135.96 (C8a-naphth); 141.25 (C8-G); 151.60 (C7a-A); 153.89 (C2-A); 154.34 (C4-G); 155.90 (C2-G); 160.32 (C4-A); 160.72 (C6-G). ^31^P NMR (^1^H-dec, 202.4 MHz, D_2_O): −0.12 and −0.96. ESI MS *m*/*z* (rel. %): 398 (100) [M–2H]^2–^, 798 (47) [M–H]^−^, 820 (35) [M–2H + Na]^−^ HR MS (ESI): for C_31_H_29_O_13_N_9_P_2_ [M–2H]^2–^, calcd 398.56855; found, 398.56823.

##### Cyclo-4-amino-5-(naphthalen-1-yl)-7-β-d-ribofuranosyl-7*H*-pyrrolo[2,3-*d*]pyrimidine 5′-*O*-phosphate (3′ → 5′) Guanosine 5′-*O*-phosphate (2′ → 5′) Sodium Salt (**5h**)

CDN **5h** was prepared as described for **5g** from iodinated CDN **5a** (15 mg, 17.8 μmol) and naphthalene-1-boronic acid (17.6 mg, 88.9 μmol). The final product was purified by HPLC (5–30% MeCN in 0.1 M TEAB). After HPLC repurification (5–50% MeOH in 0.1 M TEAB) and conversion to a sodium salt form on a Dowex 50WX8 (in a Na^+^ cycle), CDN **5h** (5.6 mg, 37%) was obtained as a white lyophilizate (water). The final product was a mixture of two diastereomers (63:37) due to hindered rotation (at 298 K). Chemical shifts of the resolved signals of minor isomer are given in italics. ^1^H NMR (600 MHz, D_2_O): 4.125 (dt, 1H, *J*_5′b,4′_ = 2.1, *J*_5′b,5′a_ = 11.8, *J*_5′b,P_ = 2.2, H5′b-G); *4.13* (ddd, 1H, *J*_5′b,4′_ = 2.3, *J*_5′b,5′a_ = 11.9, *J*_5′b,P_ = 3.5, H5′b-G); 4.23 (dd, 1H, *J*_5′a,4′_ = 2.6, *J*_5′a,5′b_ = 11.8, H5′a-G); *4.24* (dd, 1H, *J*_5′a,4′_ = 2.6, *J*_5′a,5′b_ = 11.9, H5′a-G); *4.25* (m, 1H, H5′b-A); 4.28 (ddd, 1H, *J*_5′b,4′_ = 1.5, *J*_5′b,5′a_ = 12.1, *J*_5′b,P_ = 2.8, H5′b-A); *4.385* (m, 1H, H4′-G); 4.39 (m, 1H, H4′-G); 4.44 (dm, 1H, *J*_5′a,5′b_ = 12.1, H5′a-A); *4.47* (dm, 1H, *J*_4′,3′_ = 8.4, H4′-A); 4.50 (dm, 1H, *J*_4′,3′_ = 9.0, H4′-A); 4.615 (d, 1H, *J*_3′,4′_ = 4.1, H3′-G); *4.64* (d, 1H, *J*_3′,4′_ = 4.1, H3′-G); 5.025 (d, 1H, *J*_2′,3′_ = 4.3, H2′-A); *5.04* (dd, 1H, *J*_2′,3′_ = 4.4, *J*_2′,1′_ = 1.1, H2′-A); 5.09 (ddd, 1H, *J*_3′,2′_ = 4.3, *J*_3′,4′_ = 9.0, *J*_3′,P_ = 6.2, H3′-A); *5.34* (ddd, 1H, *J*_3′,2′_ = 4.4, *J*_3′,4′_ = 8.4, *J*_3′,P_ = 6.6, H3′-A); 5.79 (ddd, 1H, *J*_2′,1′_ = 8.6, *J*_2′,3′_ = 4.1, *J*_2′,P_ = 3.7, H2′-G); *5.87* (dt, 1H, *J*_2′,1′_ = 8.6, *J*_2′,3′_ = 4.1, *J*_2′,P_ = 3.9, H2′-G); 5.97 (d, 1H, *J*_1′,2′_ = 8.6, H1′-G); *5.98* (d, 1H, *J*_1′,2′_ = 8.6, H1′-G); 6.38 (s, 1H, H1′-A); 6.55 (d, 1H, *J*_1′,2′_ = 1.1, H1′-A); 6.895 (dd, 1H, *J*_2,3_ = 7.0, *J*_2,4_ = 1.3, H8-naphth); *7.18* (dd, 1H, *J*_3,4_ = 8.3, *J*_3,2_ = 7.0, H3-naphth); *7.21* (s, 1H, H6-A); 7.36 (dd, 1H, *J*_3,4_ = 8.4, *J*_3,2_ = 7.0, H3-naphth); 7.38 (ddd, 1H, *J*_6,7_ = 8.2, *J*_6,5_ = 6.8, *J*_6,8_ = 1.5, H6-naphth); *7.38* (dd, 1H, *J*_2,3_ = 7.0, *J*_2,4_ = 1.5, H8-naphth); 7.41 (ddd, 1H, *J*_7,8_ = 6.8, *J*_7,6_ = 8.2, *J*_7,5_ = 1.4, H7-naphth); *7.485* (ddd, 1H, *J*_6,7_ = 8.5, *J*_6,5_ = 6.8, *J*_6,8_ = 1.3, H6-naphth); 7.52 (s, 1H, H6-A); *7.59* (ddd, 1H, *J*_7,8_ = 6.8, *J*_7,6_ = 8.5, *J*_7,5_ = 1.3, H7-naphth); *7.64* (dd, 1H, *J*_5,6_ = 6.8, *J*_5,7_ = 1.3, H5-naphth); 7.70 (dd, 1H, *J*_5,6_ = 6.8, *J*_5,7_ = 1.4, H5-naphth); *7.86* (dd, 1H, *J*_4,3_ = 8.3, *J*_4,2_ = 1.5, H4-naphth); 7.90 (dd, 1H, *J*_8,7_ = 6.8, *J*_8,6_ = 1.5, H8-naphth); 7.905 (dd, 1H, *J*_4,3_ = 8.4, *J*_4,2_ = 1.3, H4-naphth); 7.91 (s, 1H, H8-G); *7.995* (dd, 1H, *J*_8,7_ = 6.8, *J*_8,6_ = 1.3, H8-naphth); *8.20* (s, 1H, H2-A); 8.235 (s, 1H, H2-A). ^13^C NMR (150.9 MHz, D_2_O): 65.48 (d, *J*_C,P_ = 4.5, C5′-A); *68.92* (d, *J*_C,P_ = 5.5, C5′-G); *65.98* (d, *J*_C,P_ = 5.0, C5′-A); 68.79 (d, *J*_C,P_ = 5.1, C5′-G); 73.59 (d, *J*_C,P_ = 5.4, C3′-A); *73.71* (d, *J*_C,P_ = 5.3, C3′-A); 74.19 (C3′-G); *74.32* (C3′-G); 76.70 (C2′-A); *76.84* (C2′-A); 77.34 (d, *J*_C,P_ = 5.2, C2′-G); *77.50* (d, *J*_C,P_ = 5.5, C2′-G); 82.55 (t, *J*_C,P_ = 11.3, C4′-A); *82.83* (t, *J*_C,P_ = 11.2, C4′-A); *85.72* (d, *J*_C,P_ = 9.1, C4′-G); 85.75 (d, *J*_C,P_ = 9.4, C4′-G); *88.63* (d, *J*_C,P_ = 12.5, C1′-G); 89.02 (d, *J*_C,P_ = 13.0, C1′-G); *90.32* (C1′-A); 91.67 (C1′-A); 105.81 (C4a-A); *106.86* (C4a-A); 117.04 (C5-A); *118.96* (C5-A); 119.75 (C5-G); *119.79* (C5-G); 123.68 (C6-A); *123.73* (C6-A); *127.65* (C5-naphth); 127.82 (C5-naphth); *128.11* (C3-naphth); 128.24 (C3-naphth); 128.92 (C7-naphth); *129.03* (C7-naphth); *129.64* (C6-naphth); 129.69 (C6-naphth); 130.82 (C2-naphth); 130.97 (C4-naphth); 131.06 (C8-naphth); *131.26* (C4-naphth); *131.45* (C2-naphth); *131.48* (C8-naphth); *132.48* (C4a-naphth); 132.73 (C4a-naphth); 134.11 (C8a-naphth); *134.28* (C8a-naphth); 136.12 (C1-naphth); 136.20 (C1-naphth); *142.91* (C8-G); 142.91 (C8-G); 151.38 (C7a-A); *152.67* (C7a-A); 154.21 (C2-A); 154.40 (C4-G); *154.40* (C4-G); *154.59* (C2-A); *156.04* (C2-G); 156.89 (C2-G); 159.90 (C4-A); *160.06* (C4-A); *160.28* (C6-G); 160.94 (C6-G). ^31^P NMR (^1^H-dec, 202.4 MHz, D_2_O): −*0.08*; −0.25; −*0.32*; −0.61. ESI MS *m*/*z* (rel. %): 398 (100) [M–2H]^2–^, 798 (25) [M–H]^−^. HR MS (ESI): for C_31_H_30_O_13_N_9_P_2_ [M–H]^−^, calcd 798.114438; found, 798.14472.

##### Cyclo-4-amino-5-(1,2-dihydroacenaphthylen-5-yl)-7-β-d-ribofuranosyl-7*H*-pyrrolo[2,3-*d*]pyrimidine 5′-*O*-phosphate (3′ → 5′) Guanosine 5′-*O*-phosphate (2′ → 5′) Sodium Salt (**5i**)

CDN **5i** was prepared as described for **5g** from iodinated CDN **5a** (15 mg, 17.8 μmol) and acenaphthene-5-boronic acid (17.6 mg, 88.9 μmol). The final product was purified by HPLC (5–30% MeCN in 0.1 M TEAB). The conversion to a sodium salt form on a Dowex 50WX8 (in a Na^+^ cycle) provided CDN **5i** (7.3 mg, 47%) as a white lyophilizate (water). The final product was a mixture of two diastereomers (78:22) due to hindered rotation (at 298 K). The hindered rotation resulted in the line broadening of most NMR signals. Only a few resolved proton signals of minor isomer could be detected, and their chemical shifts are given in italics. ^1^H NMR (600 MHz, D_2_O): 3.38 (um, 2H, 2 × H1-acenaphtylene); 3.46 (um, 2H, 2 × H2-acenaphtylene); 4.11 (dm, 1H, *J*_5′b,5′a_ = 11.9, H5′b-G); 4.23 (ddd, 1H, *J*_5′a,4′_ = 2.4, *J*_5′a,5′b_ = 11.9, *J*_5′a,P_ = 6.0, H5′a-G); 4.30 (dm, 1H, *J*_5′b,5′a_ ≈ 12.0, H5′b-A); 4.38 (dt, 1H, *J*_4′,5′a_ = 2.4, *J*_4′,5′b_ = 2.2, *J*_4′,P_ = 3.4, H4′-G); 4.43 (dm, 1H, *J*_5′a,5′b_ ≈ 12.0, H5′a-A); 4.48 (dm, 1H, *J*_4′,3′_ ≈ 9.0, H4′-A); 4.62 (bd, 1H, *J*_3′,4′_ = 3.9, H3′-G); 4.99 (br, 1H, H2′-A); 5.13, *5.30* (um, 1H, H3′-A); 5.85 (um, 1H, H2′-G); 5.96, *5.90* (bd, 1H, *J*_1′,2′_ ≈ 8.0, H1′-G); 6.44, *6.52* (s, 1H, H1′-A); 7.03, *6.96* (bd, 1H, *J*_3,4_ = 6.9, H3-acenaphtylene); 7.15 (bd, 1H, *J*_6,7_ = 6.8, H6-acenaphtylene); 7.24 (bd, 1H, *J*_4,3_ = 6.9, H4-acenaphtylene); 7.26 (bd, 1H, *J*_8,7_ = 8.0, H8-acenaphtylene); 7.29 (bt, 1H, *J*_7,8_ = 8.0, *J*_7,6_ = 6.8, H7-acenaphtylene); 7.44 (bs, 1H, H6-A); 7.85 (s, 1H, H8-G); 8.21 (s, 1H, H2-A). ^13^C NMR (150.9 MHz, D_2_O): 32.48 (C1-acenaphtylene); 32.83 (C2-acenaphtylene); 65.48 (C5′-A); 68.89 (d, *J*_C,P_ = 5.3, C5′-G); 73.69 (C3′-A); 74.29 (C3′-G); 76.93 (C2′-A); 77.37 (d, *J*_C,P_ = 4.8, C2′-G); 82.27 (C4′-A); 85.62 (d, *J*_C,P_ = 9.3, C4′-G); 88.92 (d, *J*_C,P_ = 13.9, C1′-G); 91.06 (C1′-A); 105.17 (C4a-A); 117.20 (C5-A); 119.55 (C5-G); 121.73 (C4-acenaphtylene); 122.41 (C6-acenaphtylene); 122.58 (C8-acenaphtylene); 123.48 (C6-A); 127.73 (C5a-acenaphtylene); 131.66 (C7-acenaphtylene); 132.01 (C3-acenaphtylene); 132.25 (C5-acenaphtylene); 141.70 (C8b-acenaphtylene); 142.56 (C8-G); 148.94 (C8a-acenaphtylene); 149.81 (C2a-acenaphtylene); 151.60 (C7a-A); 154.28 (C2-A); 154.24 (C4-G); 155.89 (C2-G); 160.11 (C4-A); 160.68 (C6-G); ^31^P NMR (^1^H-dec, 202.4 MHz, D_2_O): −0.22 and −0.45. ESI MS *m*/*z* (rel. %): 411 (100) [M–2H]^2–^, 824 (22) [M–H]^−^, 846 (26) [M–2H + Na]^−^. HR MS (ESI): for C_33_H_32_O_13_N_9_P_2_ [M–H]^−^, calcd 824.16003; found, 824.15900.

##### Cyclo-4-amino-5-(phenanthren-9-yl)-7-β-d-ribofuranosyl-7*H*-pyrrolo[2,3-*d*]pyrimidine 5′-*O*-phosphate (3′ → 5′) Guanosine 5′-*O*-phosphate (2′ → 5′) Sodium Salt (**5j**)

CDN **5j** was prepared as described for **5g** from iodinated CDN **5a** (15 mg, 17.8 μmol) and phenanthrene-9-boronic acid (19.4 mg, 88.9 μmol). The final product was purified by HPLC (5–30% MeCN in 0.1 M TEAB). The conversion to a sodium salt form on a Dowex 50WX8 (in a Na^+^ cycle) provided CDN **5j** (4.7 mg, 30%) as a white lyophilizate (water). The final product was a mixture of two diastereomers (63:37) due to hindered rotation (at 298 K). Chemical shifts of the resolved signals of minor isomer are given in italics. ^1^H NMR (600 MHz, D_2_O): 4.07 (dt, 1H, *J*_5′b,4′_ = 2.1, *J*_5′b,5′a_ = 11.8, *J*_5′b,P_ = 2.1, H5′b-G); *4.125* (dt, 1H, *J*_5′b,4′_ = 2.1, *J*_5′b,5′a_ = 11.8, *J*_5′b,P_ = 2.1, H5′b-G); 4.22 (ddd, 1H, *J*_5′a,4′_ = 2.4, *J*_5′a,5′b_ = 11.8, *J*_5′a,P_ = 5.1, H5′a-G); *4.25* (ddd, 1H, *J*_5′a,4′_ = 2.6, *J*_5′a,5′b_ = 11.8, *J*_5′a,P_ = 5.5, H5′a-G); *4.30* (ddd, 1H, *J*_5′b,4′_ = 1.6, *J*_5′b,5′a_ = 11.8, *J*_5′b,P_ = 2.8, H5′b-A); 4.33 (m, 2H, H5′a-A and H5′b-A); 4.35 (dt, 1H, *J*_4′,5′a_ = 2.4, *J*_4′,5′b_ = 2.1, *J*_4′,P_ = 3.6, H4′-G); *4.415* (dt, 1H, *J*_4′,5′a_ = 2.6, *J*_4′,5′b_ = 2.1, *J*_4′,P_ = 3.4, H4′-G); *4.47* (dm, 1H, *J*_5′a,5′b_ = 11.8, H5′a-A); 4.49 (dm, 1H, *J*_4′,3′_ = 8.6, H4′-A); *4.53* (dm, 1H, *J*_4′,3′_ = 9.0, H4′-A); *4.66* (d, 1H, *J*_3′,2′_ = 4.0, H3′-G); 4.67 (d, 1H, *J*_3′,2′_ = 3.9, H3′-G); *5.09* (ddd, 1H, *J*_3′,2′_ = 4.3, *J*_3′,4′_ = 9.0, *J*_3′,P_ = 6.3, H3′-A); 5.10 (d, 1H, *J*_2′,3′_ = 4.5, H2′-A); *5.12* (d, 1H, *J*_2′,3′_ = 4.3, H2′-A); 5.47 (ddd, 1H, *J*_3′,2′_ = 4.5, *J*_3′,4′_ = 8.6, *J*_3′,P_ = 6.8, H3′-A); *5.80* (ddd, 1H, *J*_2′,1′_ = 8.6, *J*_2′,3′_ = 4.0, *J*_2′,P_ = 3.5, H2′-G); 5.86 (d, 1H, *J*_1′,2′_ = 8.6, H1′-G); 5.92 (um, 1H, H2′-G); *6.06* (d, 1H, *J*_1′,2′_ = 8.6, H1′-G); *6.35* (s, 1H, H1′-A); 6.62 (s, 1H, H1′-A); *7.305* (s, 1H, H10-phen); 7.32 (s, 1H, H6-A); 7.34 (br, 1H, H8-G); *7.515* (ddd, 1H, *J*_2,1_ = 8.3, *J*_2,3_ = 6.9, *J*_2,4_ = 1.2, H2-phen); 7.61 (m,1H, H2-phen); *7.63* (m, 1H, H6-phen); 7.635 (m, 1H, H7-phen); *7.65* (s, 1H, H6-A); 7.725 (m, 2H, H6 and H8-phen); 7.75 (m, 1H, H1-phen); *7.765* (m, 1H, H3-phen); *7.77* (m, 1H, H7-phen); 7.785 (s, 1H, H10-phen); 7.79 (m, 1H, H3-phen); *7.805* (m, 1H, H1-phen); *7.90* (br, 1H, H8-G); 8.21 (s, 1H, H2-A); *8.25* (s, 1H, H2-A); 8.76 (dm, 1H, *J*_4,3_ = 8.5, H4-phen); *8.79* (dm, 1H, *J*_5,6_ = 8.5, H5-phen); *8.795* (dm, 1H, *J*_4,3_ = 8.5, H4-phen); 8.84 (dm, 1H, *J*_5,6_ = 8.5, H5-phen). ^13^C NMR (150.9 MHz, D_2_O): *65.52* (C5′-A); 66.17 (C5′-A); *68.79* (C5′-G); 69.00 (C5′-G); *73.73* (C3′-A); *74.17* (C3′-G); 74.29 (C3′-G); 74.66 (C3′-A); *76.43* (C2′-A); 76.89 (C2′-A); *77.91* (C2′-G); 77.91 (C2′-G); *82.66* (C4′-A); 82.73 (C4′-A); 85.34 (C4′-G); *85.88* (C4′-G); 88.17 (C1′-G); *88.67* (C1′-G); 90.19 (C1′-A); *91.83* (C1′-A); 105.86 (C4a-A); *106.05* (C4a-A); *116.93* (C5-A); 118.78 (C5-G); 119.24 (C5-A); *119.48* (C5-G); *123.85* (C6-A); 124.16 (C6-A); *125.23* (C4-phen); 125.34 (C4-phen); *125.60* (C5-phen); 126.24 (C5-phen); 128.53 (C8-phen); *128.79* (C8-phen); *129.70* (C7-phen); 129.76 (C7-phen); 129.84 (C3-phen); 129.84 (C6-phen); *130.00* (C2-phen); 130.03 (C2-phen); 130.88 (C8a-phen); *131.18* (C10-phen); 131.48 (C10-phen); *131.49* (C8a-phen); *131.74* (C1-phen); *132.33* (C4a-phen); 132.35 (C1-phen); 132.45 (C4a-phen); *132.84* (C4b-phen); *133.02* (C9-phen); 133.09 (C4b-phen); 133.18 (C9-phen); 141.58 (C8-G); *142.58* (C8-G); *151.23* (C7a-A); 152.86 (C7a-A); 153.68 (C4-G); *154.05* (C2-A); 154.37 (C4-G); 154.57 (C2-A); 155.96 (C2-G); *155.98* (C2-G); 159.90 (C4-A); 159.68 (C6-G); *159.74* (C6-G); *160.72* (C4-A). ^31^P NMR (^1^H-dec, 202.4 MHz, D_2_O): −0.07; *–0.23*; −0.44; *–0.68*. ESI MS *m*/*z* (rel. %): 423 (100) [M–2H]^2–^, 856 (9) [M–2H + Na]^−^. HR MS (ESI): for C_35_H_32_O_13_N_9_P_2_ [M–H]^−^, calcd 848.16003; found, 848.15924.

##### Cyclo-4-amino-5-(biphenyl-4-yl)-7-β-d-ribofuranosyl-7*H*-pyrrolo[2,3-*d*]pyrimidine 5′-*O*-phosphate (3′ → 5′) Guanosine 5′-*O*-phosphate (2′ → 5′) Sodium Salt (**5k**)

CDN **5k** was prepared as described for **5g** from iodinated CDN **5a** (15 mg, 17.8 μmol) and 4-biphenylboronic acid (17.6 mg, 88.9 μmol). The final product was purified by HPLC (5–25% MeCN in 0.1 M TEAB). The conversion to a sodium salt form on a Dowex 50WX8 (in a Na^+^ cycle) provided CDN **5k** (9.1 mg, 59%) as a white lyophilizate (water). ^1^H NMR (600 MHz, D_2_O): 4.13 (ddd, 1H, *J*_5′b,4′_ = 1.7, *J*_5′b,5′a_ = 11.7, *J*_5′b,P_ = 2.5, H5′b-G); 4.24 (ddd, 1H, *J*_5′a,4′_ = 2.7, *J*_5′a,5′b_ = 11.7, *J*_5′a,P_ = 4.7, H5′a-G); 4.32 (ddd, 1H, *J*_5′b,4′_ = 2.9, *J*_5′b,5′a_ = 11.8, *J*_5′b,P_ = 1.2, H5′b-A); 4.42 (ddd, 1H, *J*_4′,5′a_ = 2.7, *J*_4′,5′b_ = 1.7, *J*_4′,P_ = 3.7, H4′-G); 4.50 (dm, 1H, *J*_4′,3′_ = 9.3, H4′-A); 4.56 (bdd, 1H, *J*_5′a,4′_ = 2.1, *J*_5′a,5′b_ = 11.8, *J*_5′a,P_ = <1, H5′a-A); 4.68 (d, 1H, *J*_3′,4′_ = 4.1, H3′-G); 4.74 (d, 1H, *J*_2′,3′_ = 4.0, H2′-A); 4.97 (ddd, 1H, *J*_3′,2′_ = 4.0, *J*_3′,4′_ = 9.3, *J*_3′,P_ = 6.5, H3′-A); 5.64 (um, 1H, H2′-G); 6.02 (d, 1H, *J*_1′,2′_ = 8.5, H1′-G); 6.25 (s, 1H, H1′-A); 7.11 (m, 2H, H3-phenylene + H5-phenylene); 7.41 (m, 1H, H4′-Ph); 7.48 (m, 2H, H2-phenylene + H6-phenylene); 7.51 (m, 2H, H3′-Ph + H5′-Ph); 7.66 (s, 1H, H6-A); 7.71 (m, 2H, H2′-Ph + H6′-Ph); 7.82 (s, 1H, H8-G); 8.18 (s, 1H, H2-A). ^13^C NMR (150.9 MHz, D_2_O): 65.54 (d, *J*_C,P_ = 4.8, C5′-A); 68.72 (d, *J*_C,P_ = 5.2, C5′-G); 73.09 (d, *J*_C,P_ = 6.5, C3′-A); 73.80 (C3′-G); 76.97 (C2′-A); 78.50 (C2′-G); 82.55 (t, *J*_C,P1_ = *J*_C,P2_ = 11.3, C4′-A); 86.10 (d, *J*_C,P_ = 9.2, C4′-G); 88.07 (C1′-G); 92.24 (C1′-A); 103.65 (C4a-A); 119.08 (C5-A); 119.67 (C5-G); 122.79 (C6-A); 129.37 (C2′-Ph + C6′-Ph); 129.67 (C2-phenylene + C6-phenylene); 130.31 (C4′-Ph); 130.49 (C3-phenylene + C5-phenylene); 131.82 (C3′-Ph + C5′-Ph); 134.81 (C4-phenylene); 140.79 (C1-phenylene); 141.33 (C8-G); 142.49 (C1′-Ph); 151.25 (C7a-A); 153.39 (C2-A); 154.49 (C4-G); 155.98 (C2-G); 159.95 (C4-A); 160.98 (C6-G). ^31^P NMR (^1^H-dec, 202.4 MHz, D_2_O): −0.14 and −0.93. ESI MS *m*/*z* (rel. %): 411 (100) [M–2H]^2–^, 824 (43) [M–H]^−^, 846 (17) [M–2H + Na]^−^. HR MS (ESI): for C_33_H_32_O_13_N_9_P_2_ [M–H]^−^, calcd 824.16003; found, 824.16012.

##### Cyclo-4-amino-5-(4-(naphthalen-2-yl)phenyl)-7-β-d-ribofuranosyl-7*H*-pyrrolo[2,3-*d*]pyrimidine 5′-*O*-phosphate (3′ → 5′) Guanosine 5′-*O*-phosphate (2′ → 5′) Sodium Salt (**5l**)

CDN **5l** was prepared as described for **5g** from iodinated CDN **5a** (15 mg, 17.8 μmol) and 4-(naphthalene-2-yl)phenylboronic acid pinacol ester (29.4 mg, 88.9 μmol). The final product was purified by HPLC (5–30% MeCN in 0.1 M TEAB). The conversion to a sodium salt form on a Dowex 50WX8 (in a Na^+^ cycle) provided CDN **5l** (5.3 mg, 32%) as a white lyophilizate (water). ^1^H NMR (600 MHz, D_2_O): 4.14 (ddd, 1H, *J*_5′b,4′_ = 1.8, *J*_5′b,5′a_ = 11.7, *J*_5′b,P_ = 2.4, H5′b-G); 4.22 (ddd, 1H, *J*_5′a,4′_ = 2.5, *J*_5′a,5′b_ = 11.7, *J*_5′a,P_ = 4.8, H5′a-G); 4.35 (ddd, 1H, *J*_5′b,5′a_ = 11.9, *J*_5′b,4′_ = 1.1, *J*_5′b,P_ = 2.7, H5′b-A); 4.44 (ddd, 1H, *J*_4′,5′a_ = 2.5, *J*_4′,5′b_ = 1.8, *J*_4′,P_ = 3.7, H4′-G); 4.52 (dm, 1H, *J*_4′,3′_ = 9.3, H4′-A); 4.58 (bdd, 1H, *J*_5′a,5′b_ = 11.9, *J*_5′a,4′_ = 2.0, *J*_5′b,P_ = <1, H5′a-A); 4.70 (d, 1H, *J*_3′,2′_ = 4.0, H3′-G); 4.76 (overlap, H2′-A); 4.98 (ddd, 1H, *J*_3′,2′_ = 4.0, *J*_3′,4′_ = 9.3, *J*_3′,P_ = 6.6, H3′-A); 5.61 (um, 1H, H2′-G); 6.03 (d, 1H, *J*_1′,2′_ = 8.5, H1′-G); 6.30 (s, 1H, H1′-A); 7.16 (m, 2H, H2 + H6-phenylene); 7.53 (m, 1H, H7-naphth); 7.54 (m, 1H, H6-naphth); 7.57 (m, 2H, H3 + H5-phenylene); 7.65 (s, 1H, H6-A); 7.81 (s, 1H, H8-G); 7.84 (dd, 1H, *J*_3,1_ = 1.9, *J*_3,4_ = 8.6, H3-naphth); 7.90 (m, 1H, H8-naphth); 7.91 (m, 1H, H5-naphth); 7.94 (d, 1H, *J*_4,3_ = 8.6, H4-naphth); 8.12 (bd, 1H, *J*_1,3_ = 1.9, H1-naphth); 8.12 (s, 1H, H2-A). ^13^C NMR (150.9 MHz, D_2_O): 65.71 (d, *J*_C,P_ = 3.2, C5′-A); 68.78 (d, *J*_C,P_ = 4.8, C5′-G); 73.19 (d, *J*_C,P_ = 6.5, C3′-A); 73.96 (C3′-G); 76.98 (C2′-A); 78.87 (C2′-G); 82.58 (t, *J*_C,P1_ = *J*_C,P2_ = 11.4, C4′-A); 86.12 (d, *J*_C,P_ = 9.5, C4′-G); 87.90 (d, *J*_C,P_ = 13.9, C1′-G); 92.18 (C1′-A); 103.64 (C4a-A); 119.16 (C5-A); 119.61 (C5-G); 122.71 (C6-A); 127.76 (C1-naphth); 127.81 (C3-naphth); 128.93 (C7-naphth); 129.24 (C6-naphth); 129.89 (C3 and C5-phenylene); 130.24 (C8-naphth); 130.57 (C2 and C6-phenylene); 130.82 (C5-naphth); 131.15 (C4-naphth); 134.88 (C1-phenylene); 134.99 (C4a-naphth); 136.05 (C8a-naphth); 139.85 (C2-naphth); 140.55 (C4-phenylene); 141.08 (C8-G); 151.23 (C7a-A); 153.51 (C2-A); 154.49 (C4-G); 156.00 (C2-G); 159.89 (C4-A); 160.98 (C6-G). ^31^P NMR (^1^H-dec, 202.4 MHz, D_2_O): −0.14 and −0.93. ESI MS *m*/*z* (rel. %): 436 (100) [M–2H]^2–^, 874 (17) [M–H]^−^, 896 (14) [M–2H + Na]^−^. HR MS (ESI): for C_37_H_34_O_13_N_9_P_2_ [M–H]^−^, calcd 874.17568; found, 874.17462.

##### Cyclo-4-amino-5-[4-{(naphthalen-1-yloxy)methyl}phenyl]-7-β-d-ribofuranosyl-7*H*-pyrrolo[2,3-*d*]pyrimidine 5′-*O*-phosphate (3′ → 5′) Guanosine 5′-*O*-phosphate (2′ → 5′) Sodium Salt (**5m**)

CDN **5m** was prepared as described for **5g** from iodinated CDN **5a** (15 mg, 17.8 μmol) and [(1-naphthyloxy)methyl]phenylboronic acid (24.7 mg, 88.9 μmol). The final product was purified by HPLC (5–32.5% MeCN in 0.1 M TEAB). The conversion to a sodium salt form on a Dowex 50WX8 (in a Na^+^ cycle) provided CDN **5m** (5.7 mg, 34%) as a white lyophilizate (water). ^1^H NMR (600 MHz, D_2_O): 4.13 (dt, 1H, *J*_5′b,4′_ = 1.8, *J*_5′b,5′a_ = 11.8, *J*_5′b,P_ = 1.8, H5′b-G); 4.24 (ddd, 1H, *J*_5′a,4′_ = 2.7, *J*_5′a,5′b_ = 11.8, *J*_5′a,P_ = 4.8, H5′a-G); 4.30 (dm, 1H, *J*_5′b,5′a_ = 11.7, H5′b-A); 4.42 (m, 1H, H4′-G); 4.49 (dm, 1H, *J*_4′,3′_ = 9.5, H4′-A); 4.53 (dm, 1H, *J*_5′a,5′b_ = 11.7, H5′a-A); 4.66 (d, 1H, *J*_3′,4′_ = 4.0, H3′-G); 4.72 (bd, 1H, *J*_2′,3′_ = 4.0, H2′-A); 4.97 (ddd, 1H, *J*_3′,2′_ = 4.0, *J*_3′,4′_ = 9.5, *J*_3′,P_ = 6.5, H3′-A); 5.26 (d, 1H, *J*_gem_ = 11.5, O-CHaHb); 5.30 (d, 1H, *J*_gem_ = 11.5, O-CHaHb); 5.68 (dt, 1H, *J*_2′,1′_ = 8.5, *J*_2′,3′_ = 4.0, *J*_2′,P_ = 4.0, H2′-G); 5.97 (d, 1H, *J*_1′,2′_ = 8.5, H1′-G); 6.11 (s, 1H, H1′-A); 7.09 (bd, 1H, *J*_2,3_ = 7.6, H2-naphth-A); 7.12 (m, 2H, H2 and H6-phenylene); 7.34 (m, 2H, H3 and H5-phenylene); 7.47 (m, 2H, H3 and H7-naphth-A); 7.54 (s, 1H, H6-A); 7.54 (m, 2H, H4 and H6-naphth-A); 7.76 (s, 1H, H8-G); 7.87 (bd, 1H, *J*_5,6_ = 8.3, H5-naphth-A); 8.03 (s, 1H, H2-A); 8.17 (bd, 1H, *J*_8,7_ = 8.5, H8-naphth-A). ^13^C NMR (150.9 MHz, D_2_O): 65.51 (d, *J*_C,P_ = 4.0, C5′-A); 68.67 (d, *J*_C,P_ = 5.1, C5′-G); 73.02 (CH_2_O); 73.15 (d, *J*_C,P_ = 5.6, C3′-A); 73.91 (C3′-G); 76.88 (C2′-A); 78.06 (d, *J*_C,P_ = 5.8, C2′-G); 82.56 (t, *J*_C,P1_ = *J*_C,P2_ = 11.2, C4′-A); 85.95 (d, *J*_C,P_ = 9.4, C4′-G); 88.51 (d, *J*_C,P_ = 12.2, C1′-G); 92.09 (C1′-A); 103.57 (C4a-A); 109.29 (C2-naphth); 118.94 (C5-A); 119.81 (C5-G); 122.80 (C6-A); 123.49 (C4-naphth); 124.15 (C8-naphth); 127.72 (C4a-naphth); 128.35 (C7-naphth); 129.04 (C3-naphth); 129.41 (C6-naphth); 130.23 (C5-naphth); 130.46 (C2 and C6-phenylene); 130.98 (C3 and C5-phenylene); 136.61 (C1-phenylene); 136.86 (C8a-naphth-A); 137.84 (C4-phenylene); 142.06 (C8-G); 150.92 (C7a-A); 153.37 (C2-A); 154.50 (C4-G); 155.92 (C2-G); 156.43 (C1-naphth-A); 159.61 (C4-A); 161.05 (C6-G). ^31^P NMR (^1^H-dec, 202.4 MHz, D_2_O): −0.21 and −0.96. ESI MS *m*/*z* (rel. %): 451 (100) [M–2H]^2–^, 462 (5) [M–3H + Na]^2–^, 904 (6) [M–H]^−^. HR MS (ESI): for C_38_H_36_O_14_N_9_P_2_ [M–H]^−^, calcd 904.18624; found, 904.18469.

##### Cyclo-4-amino-5-(furan-2-yl)-7-β-d-ribofuranosyl-7*H*-pyrrolo[2,3-*d*]pyrimidine 5′-*O*-phosphate (3′ → 5′) Guanosine 5′-*O*-phosphate (2′ → 5′) Sodium Salt (**5n**)

NTP **3n** (2 μmol) and GTP (2 μmol) were enzymatically cyclized using mcGAS. The final product was purified by HPLC (5–30% MeCN in 0.1 M TEAB). The conversion to a sodium salt form on a Dowex 50WX8 (in a Na^+^ cycle) provided CDN **5n** (299 nmol, 15%). ^1^H NMR (600 MHz, D_2_O): 4.13 (ddd, 1H, *J*_5′b,4′_ = 1.9, *J*_5′b,5′a_ = 11.8, *J*_5′b,P_ = 2.0, H5′b-G); 4.23 (ddd, 1H, *J*_5′a,4′_ = 3.1, *J*_5′a,5′b_ = 11.8, *J*_5′a,P_ = 4.5, H5′a-G); 4.34 (ddd, 1H, *J*_5′b,5′a_ = 12.0, *J*_5′b,4′_ = 2.7, *J*_5′b,P_ = 1.1, H5′b-A); 4.42 (ddd, 1H, *J*_4′,5′a_ = 3.1, *J*_4′,5′b_ = 1.9, *J*_4′,P_ = 3.6, H4′-G); 4.50 (dm, 1H, *J*_4′,3′_ = 9.7, H4′-A); 4.61 (bdd, 1H, *J*_5′a,5′b_ = 12.0, *J*_5′a,4′_ = 2.2, *J*_5′a,P_ < 1.0, H5′a-A); 4.66 (d, 1H, *J*_3′,4′_ = 4.0, H3′-G); 4.66 (d, 1H, *J*_2′,3′_ = 4.0, H2′-A); 5.01 (ddd, 1H, *J*_3′,2′_ = 4.0, *J*_3′,4′_ = 9.7, *J*_3′,P_ = 6.6, H3′-A); 5.74 (ddd, 1H, *J*_2′,1′_ = 8.7, *J*_2′,3′_ = 4.0, *J*_2′,P_ = 3.8, H2′-G); 5.93 (d, 1H, *J*_1′,2′_ = 8.7, H1′-G); 6.27 (s, 1H, H1′-A); 6.26 (dd, 1H, *J*_4,3_ = 3.5, *J*_4,5_ = 1.8, H4-furyl); 6.28 (dd, 1H, *J*_3,4_ = 3.5, *J*_3,5_ = 0.8, H3-furyl); 7.42 (dd, 1H, *J*_5,4_ = 1.8, *J*_5,3_ = 0.8, H5-furyl); 7.75 (s, 1H, H8-G); 8.00 (s, 1H, H6-A); 8.19 (s, 1H, H2-A). ^13^C NMR (150.9 MHz, D_2_O): 65.48 (d, *J*_C,P_ = 4.0, C5′-A); 68.56 (d, *J*_C,P_ = 5.2, C5′-G); 72.66 (d, *J*_C,P_ = 5.3, C3′-A); 73.70 (C3′-G); 76.92 (C2′-A); 77.59 (br, C2′-G); 82.34 (t, *J*_C,P1_ = *J*_C,P2_ = 11.5, C4′-A); 85.82 (d, *J*_C,P_ = 10.2, C4′-G); 88.84 (d, *J*_C,P_ = 15.0, C1′-G); 92.50 (C1′-A); 102.40 (C5-A); 106.58 (C3-furyl); 109.14 (C4a-A); 114.75 (C4-furyl); 119.79 (C5-G); 120.95 (C6-A); 142.52 (C8-G); 143.61 (C5-furyl); 150.48 (C2-furyl); 150.75 (C7a-A); 154.03 (C2-A); 154.47 (C4-G); 155.82 (C2-G); 159.59 (C4-A); 161.11 (C6-G). ^31^P NMR (^1^H-dec, 202.4 MHz, D_2_O): −0.16 and −1.18. ESI MS *m*/*z* (rel. %): 368 (100) [M–2H]^2–^, 379 (6) [M–3H–Na]^2–^, 738 (14) [M–H]^−^, 760 (14) [M–2H + Na]^−^. HR MS (ESI): for C_25_H_26_O_14_N_9_P_2_ [M–H]^−^, calcd 738.10799; found, 738.10760.

##### Cyclo-4-amino-7-β-d-ribofuranosyl-5-(thiophen-2-yl)-7*H*-pyrrolo[2,3-*d*]pyrimidine 5′-*O*-phosphate (3′ → 5′) Guanosine 5′-*O*-phosphate (2′ → 5′) Sodium Salt (**5o**)

NTP **3o** (2 μmol) and GTP (2 μmol) were enzymatically cyclized using mcGAS. The final product was purified by HPLC (5–30% MeCN in 0.1 M TEAB). The conversion to a sodium salt form on a Dowex 50WX8 (in a Na^+^ cycle) provided CDN **5o** (266 nmol, 13%). ^1^H NMR (600 MHz, D_2_O): 4.15 (ddd, 1H, *J*_5′b,4′_ = 1.8, *J*_5′b,5′a_ = 11.8, *J*_5′b,P_ = 2.2, H5′b-G); 4.29 (ddd, 1H, *J*_5′a,4′_ = 2.8, *J*_5′a,5′b_ = 11.8, *J*_5′a,P_ = 4.9, H5′a-G); 4.31 (ddd, 1H, *J*_5′b,5′a_ = 12.0, *J*_5′b,4′_ = 3.1, *J*_5′b,P_ = 1.2, H5′b-A); 4.44 (ddd, 1H, *J*_4′,5′a_ = 2.8, *J*_4′,5′b_ = 1.8, *J*_4′,P_ = 3.6, H4′-G); 4.49 (dm, 1H, *J*_4′,3′_ = 9.2, H4′-A); 4.52 (dm, 1H, *J*_5′a,5′b_ = 12.0, H5′a-A); 4.66 (d, 1H, *J*_3′,4′_ = 4.0, H3′-G); 4.79 (overlap, H2′-A); 5.00 (ddd, 1H, *J*_3′,2′_ = 4.2, *J*_3′,4′_ = 9.2, *J*_3′,P_ = 6.5, H3′-A); 5.70 (dt, 1H, *J*_2′,1′_ = 8.7, *J*_2′,3′_ = 4.0, *J*_2′,P_ = 4.0, H2′-G); 5.99 (d, 1H, *J*_1′,2′_ = 8.7, H1′-G); 6.28 (s, 1H, H1′-A); 6.82 (dd, 1H, *J*_5,4_ = 3.5, *J*_5,3_ = 1.2, H5-thienyl); 6.98 (dd, 1H, *J*_4,3_ = 5.2, *J*_4,5_ = 3.5, H4-thienyl); 7.24 (dd, 1H, *J*_3,4_ = 5.2, *J*_3,5_ = 1.2, H3-thienyl); 7.65 (s, 1H, H6-A); 7.87 (s, 1H, H8-G); 8.24 (s, 1H, H2-A). ^13^C NMR (150.9 MHz, D_2_O): 65.53 (d, *J*_C,P_ = 4.2, C5′-A); 68.66 (d, *J*_C,P_ = 5.2, C5′-G); 73.03 (d, *J*_C,P_ = 5.6, C3′-A); 73.86 (C3′-G); 76.84 (C2′-A); 77.74 (C2′-G); 82.62 (t, *J*_C,P_ = 11.1, C4′-A); 85.96 (d, *J*_C,P_ = 9.8, C4′-G); 88.78 (d, *J*_C,P_ = 9.8, C1′-G); 92.20 (C1′-A); 103.70 (C5-A); 112.08 (C4a-A); 120.00 (C5-G); 123.16 (C6-A); 128.07 (C3-thienyl); 128.26 (C5-thienyl); 130.70 (C4-thienyl); 137.46 (C2-thienyl); 142.56 (C8-G); 151.16 (C7a-A); 154.12 (C2-A); 154.69 (C4-G); 155.97 (C2-G); 160.00 (C4-A); 161.29 (C6-G). ^31^P NMR (^1^H-dec, 202.4 MHz, D_2_O): −0.24 and −0.93. ESI MS *m*/*z* (rel. %): 376 (100) [M–2H]^2–^, 387 (4) [M–H + Na]^2–^, 754 (4) [M–H]^−^, 776 (10) [M–2H + Na]^−^. HR MS (ESI): for C_25_H_26_O_13_N_9_P_2_S [M–H]^−^, calcd 754.08515; found, 754.08502.

##### Cyclo-4-amino-5-(benzofuran-2-yl)-7-β-d-ribofuranosyl-7*H*-pyrrolo[2,3-*d*]pyrimidine 5′-*O*-phosphate (3′ → 5′) Guanosine 5′-*O*-phosphate (2′ → 5′) Sodium Salt (**5p**)

CDN **5p** was prepared as described for **5g** from iodinated CDN **5a** (15 mg, 17.8 μmol) and benzofuran-2-ylboronic acid (14.4 mg, 88.9 μmol). After two HPLC purifications (5–25% MeCN in 0.1 M TEAB and 5–50% MeOH in 0.1 M TEAB) and conversion to a sodium salt form on a Dowex 50WX8 (in a Na^+^ cycle), CDN **5p** (8.1 mg, 55%) was obtained as a white lyophilizate (water). ^1^H NMR (600 MHz, D_2_O): 4.07 (ddd, 1H, *J*_5′b,4′_ = 1.8, *J*_5′b,5′a_ = 11.8, *J*_5′b,P_ = 2.4, H5′b-G); 4.20 (ddd, 1H, *J*_5′a,4′_ = 2.9, *J*_5′a,5′b_ = 11.8, *J*_5′a,P_ = 4.2, H5′a-G); 4.35 (ddd, 1H, *J*_5′b,4′_ = 1.0, *J*_5′b,5′a_ = 11.8, *J*_5′b,P_ = 2.6, H5′b-A); 4.38 (ddd, 1H, *J*_4′,5′a_ = 2.9, *J*_4′,5′b_ = 1.8, *J*_4′,P_ = 3.6, H4′-G); 4.51 (dm, 1H, *J*_4′,3′_ = 9.5, H4′-A); 4.59 (d, 1H, *J*_2′,3′_ = 3.8, H2′-A); 4.65 (bdd, 1H, *J*_5′a,4′_ = 2.0, *J*_5′a,5′b_ = 11.8, *J*_5′a,P_ < 1, H5′a-A); 4.67 (d, 1H, *J*_3′,4′_ = 3.8, H3′-G); 4.93 (ddd, 1H, *J*_3′,2′_ = 3.8, *J*_3′,4′_ = 9.5, *J*_3′,P_ = 6.5, H3′-A); 5.62 (um, H2′-G); 5.90 (d, 1H, *J*_1′,2′_ = 8.5, H1′-G); 6.18 (s, 1H, H1′-A); 6.65 (d, 1H, *J*_3,4_ = 3.8, H3-benzofuryl); 7.22 (td, 1H, *J*_5,6_ = 6.8, *J*_5,4_ = 6.8, *J*_5,7_ = 1.3, H5-benzofuryl); 7.24 (ddd, 1H, *J*_6,5_ = 6.8, *J*_6,7_ = 7.1, *J*_6,4_ = 1.6, H6-benzofuryl); 7.42 (m, 2H, H4-benzofuryl + H7-benzofuryl); 7.47 (s, 1H, H8-G); 8.09 (s, 1H, H2-A); 8.10 (s, 1H, H6-A). ^13^C NMR (150.9 MHz, D_2_O): 65.53 (C5′-A); 68.59 (d, *J*_C,P_ = 3.7, C5′-G); 72.68 (d, *J*_C,P_ = 4.3, C3′-A); 73.74 (C3′-G); 78.30 (d, *J*_C,P_ = 5.4, C2′-G); 78.86 (C2′-A); 82.33 (t, *J*_C,P1_ = *J*_C,P2_ = 10.6, C4′-A); 85.83 (d, *J*_C,P_ = 6.3, C4′-G); 88.02 (d, *J*_C,P_ = 12.0, C1′-G); 92.25 (C1′-A); 102.44 (C4a-A); 102.51 (C3-benzofuryl); 108.58 (C5-A); 113.48 (C7-benzofuryl); 119.31 (C5-G); 122.77 (C6-A); 123.46 (C4-benzofuryl); 125.99 (C5-benzofuryl); 126.32 (C6-benzofuryl); 131.49 (C3a-benzofuryl); 141.30 (C8-G); 150.77 (C7a-A); 152.57 (C2-benzofuryl); 153.71 (C2-A); 154.26 (C4-G); 155.77 (C2-G); 156.02 (C7a-benzofuryl); 159.60 (C4-A); 160.54 (C6-G). ^31^P NMR (^1^H-dec, 202.4 MHz, D_2_O): −0.10 and −1.10. ESI MS *m*/*z* (rel. %): 393 (100) [M–2H]^2–^, 788 (44) [M–H]^−^, 810 (21) [M–2H + Na]^−^. HR MS (ESI): for C_29_H_28_O_14_N_9_P_2_ [M–H]^−^, calcd 788.12364; found, 788.12413.

##### Cyclo-4-amino-5-(benzothiophen-2-yl)-7-β-d-ribofuranosyl-7*H*-pyrrolo[2,3-*d*]pyrimidine 5′-*O*-phosphate (3′ → 5′) Guanosine 5′-*O*-phosphate (2′ → 5′) Sodium Salt (**5q**)

CDN **5q** was prepared as described for **5g** from iodinated CDN **5a** (12 mg, 14.2 μmol) and benzothiophen-2-ylboronic acid (12.6 mg, 71.1 μmol). After two HPLC purifications (5–25% MeCN in 0.1 M TEAB and 9–24% MeCN in 0.1 M TEAB) and conversion to a sodium salt form on a Dowex 50WX8 (in a Na^+^ cycle), CDN **5q** (4.8 mg, 40%) was obtained as a white lyophilizate (water). ^1^H NMR (600 MHz, D_2_O): 4.12 (ddd, 1H, *J*_5′b,4′_ = 1.6, *J*_5′b,5′a_ = 11.8, *J*_5′b,P_ = 2.5, H5′b-G); 4.25 (ddd, 1H, *J*_5′a,4′_ = 2.6, *J*_5′a,5′b_ = 11.8, *J*_5′a,P_ = 4.4, H5′a-G); 4.35 (ddd, 1H, *J*_5′b,4′_ ∼ 1.0, *J*_5′b,5′a_ = 12.0, *J*_5′b,P_ = 2.5, H5′b-A); 4.44 (ddd, 1H, *J*_4′,5′a_ = 2.6, *J*_4′,5′b_ = 1.6, *J*_4′,P_ = 3.8, H4′-G); 4.52 (dm, 1H, *J*_4′,3′_ = 9.3, H4′-A); 4.57 (dm, 1H, *J*_5′a,5′b_ = 12.0, H5′a-A); 4.71 (d, 1H, *J*_3′,4′_ = 4.0, H3′-G); 4.77 (overlap, H2′-A); 4.95 (ddd, 1H, *J*_3′,2′_ = 4.0, *J*_3′,4′_ = 9.3, *J*_3′,P_ = 6.7, H3′-A); 5.60 (um, 1H, H2′-G); 6.03 (d, 1H, *J*_1′,2′_ = 8.7, H1′-G); 6.30 (s, 1H, H1′-A); 7.10 (s, 1H, H3-benzothienyl); 7.36 (ddd, 1H, *J*_6,4_ = 1.3, *J*_6,5_ = 7.0, *J*_6,7_ = 8.0, H6-benzothienyl); 7.41 (ddd, 1H, *J*_5,4_ = 8.0, *J*_5,6_ = 7.0, *J*_5,7_ = 1.2, H5-benzothienyl); 7.72 (dd, 1H, *J*_4,5_ = 8.0, *J*_4,6_ = 1.3, H4-benzothienyl); 7.75 (s, 1H, H6-A); 7.78 (s, 1H, H8-G); 7.82 (dd, 1H, *J*_7,6_ = 8.0, *J*_7,5_ = 1.2, H7-benzothienyl); 8.23 (s, 1H, H2-A). ^13^C NMR (150.9 MHz, D_2_O): 65.61 (b, C5′-A); 68.75 (d, *J*_C,P_ = 4.3, C5′-G); 72.96 (d, *J*_C,P_ = 5.4, C3′-A); 73.90 (C3′-G); 76.89 (C2′-A); 78.91 (C2′-G); 82.52 (t, *J*_C,P_ = 11.3, C4′-A); 86.08 (d, *J*_C,P_ = 9.6, C4′-G); 87.84 (C1′-G); 92.23 (C1′-A); 103.70 (C5-A); 112.55 (C4a-A); 119.66 (C5-G); 123.70 (C6-A); 124.04 (C3-benzothienyl); 124.89 (C7-benzothienyl); 126.22 (C4-benzothienyl); 126.99 (C6-benzothienyl); 127.37 (C6-benzothienyl); 141.14 (C8-G); 138.36 (C2-benzothienyl); 141.78 (C7a-benzothienyl); 142.94 (C3a-benzothienyl); 151.46 (C7a-A); 154.26 (C2-A); 154.59 (C4-G); 156.00 (C2-G); 160.18 (C4-A); 160.87 (C6-G). ^31^P NMR (^1^H-dec, 202.4 MHz, D_2_O): −0.16 and −0.88. ESI MS *m*/*z* (rel. %): 436 (100) [M–2H]^2–^, 874 (16) [M–H]^−^, 896 (14) [M–2H + Na]^−^. HR MS (ESI): for C_29_H_28_O_14_N_9_P_2_ [M–H]^−^, calcd 874.17568; found, 874.17462.

##### Cyclo-4-amino-5-(dibenzo[*b*,*d*]furan-4-yl)-7-β-d-ribofuranosyl-7*H*-pyrrolo[2,3-*d*]pyrimidine 5′-*O*-phosphate (3′ → 5′) Guanosine 5′-*O*-phosphate (2′ → 5′) Sodium Salt (**5r**) and Open Isomer **16**

CDN **5r** was prepared as described for **5g** from iodinated CDN **5a** (15 mg, 17.8 μmol) and dibenzofuran-4-ylboronic acid (19 mg, 89.6 μmol). After two purifications (5–25% MeCN in 0.1 M TEAB) and conversion to a sodium salt form on a Dowex 50WX8 (in a Na^+^ cycle), CDN **5r** (6.3 mg, 40%) was obtained as a white lyophilizate (water). As a byproduct, compound **16** was isolated and converted to a sodium salt form on a Dowex 50WX8 (in a Na^+^ cycle). Open isomer **16** (2.0 mg, 13%) was obtained as a white lyophilizate (water). CDN **5r**: ^1^H NMR (600 MHz, D_2_O): 4.10 (ddd, 1H, *J*_5′b,4′_ = 2.1, *J*_5′b,5′a_ = 11.9, *J*_5′b,P_ = 2.4, H5′b-G); 4.22 (ddd, 1H, *J*_5′a,4′_ = 2.5, *J*_5′a,5′b_ = 11.9, *J*_5′a,P_ = 5.8, H5′a-G); 4.33 (m, 1H, H5′b-A); 4.38 (ddd, 1H, *J*_4′,5′a_ = 2.5, *J*_4′,5′b_ = 2.1, *J*_4′,P_ = 3.4, H4′-G); 4.48 (m, 1H, H5′a-A); 4.49 (dm, 1H, *J*_4′,3′_ = 9.0, H4′-A); 4.64 (d, 1H, *J*_3′,4′_ = 3.5, H3′-G); 4.85 (d, 1H, *J*_2′,3′_ = 4.6, H2′-A); 5.10 (ddd, 1H, *J*_3′,2′_ = 4.6, *J*_3′,4′_ = 9.0, *J*_3′,P_ = 6.2, H3′-A); 5.84 (ddd, 1H, *J*_2′,1′_ = 8.6, *J*_2′,3′_ = 3.5, *J*_2′,P_ = 3.7, H2′-G); 5.92 (d, 1H, *J*_1′,2′_ = 8.6, H1′-G); 6.40 (s, 1H, H1′-A); 7.04 (dd, 1H, *J*_3,2_ = 7.5, *J*_3,1_ = 1.3, H3-dibenzofuryl); 7.14 (dd, 1H, *J*_2,3_ = 7.5, *J*_2,1_ = 7.8, H2-dibenzofuryl); 7.40 (td, 1H, *J*_8,9_ = 7.4, *J*_8,7_ = 7.4, *J*_8,6_ = 1.2, H8-dibenzofuryl); 7.44 (ddd, 1H, *J*_7,6_ = 8.2, *J*_7,8_ = 7.4, *J*_7,9_ = 1.5, H7-dibenzofuryl); 7.49 (s, 1H, H6-A); 7.59 (ddd, 1H, *J*_6,7_ = 8.2, *J*_6,8_ = 1.2, *J*_6,9_ = 0.7, H6-dibenzofuryl-A); 7.68 (s, 1H, H8-G); 7.76 (dd, 1H, *J*_1,2_ = 7.8, *J*_1,3_ = 1.3, H1-dibenzofuryl); 8.00 (ddd, 1H, *J*_9,8_ = 7.4, *J*_9,7_ = 1.5, *J*_9,6_ = 0.7, H9-dibenzofuryl); 8.19 (s, 1H, H2-A). ^13^C NMR (150.9 MHz, D_2_O): 65.59 (d, *J*_C,P_ = 4.9, C5′-A); 68.80 (d, *J*_C,P_ = 4.9, C5′-G); 73.79 (d, *J*_C,P_ = 5.5, C3′-A); 74.22 (C3′-G); 77.14 (C2′-A); 77.50 (d, *J*_C,P_ = 4.5, C2′-G); 82.84 (t, *J*_C,P1_ = *J*_C,P2_ = 11.2, C4′-A); 85.76 (d, *J*_C,P_ = 9.5, C4′-G); 88.79 (d, *J*_C,P_ = 12.3, C1′-G); 91.31 (C1′-A); 104.21 (C4a-A); 113.84 (C5-A); 114.61 (C6-dibenzofuryl); 119.57 (C4-dibenzofuryl); 119.60 (C5-G); 122.52 (C1-dibenzofuryl); 123.72 (C9-dibenzofuryl); 124.05 (C6-A); 125.81 (C8-dibenzofuryl); 126.08 (C2-dibenzofuryl); 126.44 (C9a-dibenzofuryl); 127.01 (C9b-dibenzofuryl); 130.15 (C7-dibenzofuryl); 130.40 (C3-dibenzofuryl); 142.45 (C8-G); 151.79 (C7a-A); 154.22 (C4-G); 154.27 (C2-A); 155.08 (C4a-dibenzofuryl); 155.88 (C2-G); 158.29 (C5a-dibenzofuryl); 160.13 (C4-A); 160.59 (C6-G). ^31^P NMR (^1^H-dec, 202.4 MHz, D_2_O): −0.19 and −0.44. ESI MS *m*/*z* (rel. %): 418 (100) [M–2H]^2–^, 838 (28) [M–H]^−^, 860 (13) [M–2H + Na]^−^. HR MS (ESI): for C_33_H_30_O_14_N_9_P_2_ [M–H]^−^, calcd 838.13929; found, 838.13778. Open isomer **16**: ^1^H NMR (600 MHz, D_2_O): 3.34 (dd, 1H, *J*_5′b,4′_ = 4.5, *J*_5′b,5′a_ = 12.8, H5′b-G); 3.46 (dd, 1H, *J*_5′a,4′_ = 2.5, *J*_5′a,5′b_ = 12.8, H5′a-G); 3.50 (dd, 1H, *J*_3′,2′_ = 4.6, *J*_3′,4′_ = 7.8, H3′-G); 3.76 (ddd, 1H, *J*_4′,3′_ = 7.8, *J*_4′,5′a_ = 2.5, *J*_4′,5′b_ = 4.5, H4′-G); 4.12 (ddd, 1H, *J*_5′b,4′_ = 2.6, *J*_5′b,5′a_ = 11.7, *J*_5′b,P_ = 4.2, H5′b-A); 4.19 (ddd, 1H, *J*_5′a,4′_ = 2.8, *J*_5′a,5′b_ = 11.7, *J*_5′a,P_ = 5.1, H5′a-A); 4.42 (ddd, 1H, *J*_2′,1′_ = 2.1, *J*_2′,3′_ = 4.6, *J*_2′,P_ = 7.4, H2′-G); 4.58 (m, 1H, *J*_4′,3′_ = 3.2, *J*_4′,5′a_ = 2.8, *J*_4′,5′b_ = 2.6, *J*_4′,P_ = 2.5, H4′-A); 5.19 (td, 1H, *J*_3′,2′_ = 6.6, *J*_3′,4′_ = 3.2, *J*_3′,P_ = 6.6, H3′-A); 5.42 (ddd, 1H, *J*_2′,1′_ = 4.3, *J*_2′,3′_ = 6.6, *J*_2′,P_ = 11.6, H2′-A); 5.60 (d, 1H, *J*_1′,2′_ = 2.1, H1′-G); 6.34 (d, 1H, *J*_1′,2′_ = 4.3, H1′-A); 7.00 (dd, 1H, *J*_3,2_ = 7.4, *J*_3,1_ = 1.3, H3-dibenzofuryl); 7.25 (dd, 1H, *J*_2,3_ = 7.4, *J*_2,1_ = 7.7, H2-dibenzofuryl); 7.39 (ddd, 1H, *J*_8,9_ = 7.7, *J*_8,7_ = 7.2, *J*_8,6_ = 1.0, H8-dibenzofuryl); 7.47 (s, 1H, H8-G); 7.49 (s, 1H, H6-A); 7.50 (ddd, 1H, *J*_7,6_ = 8.2, *J*_7,8_ = 7.2, *J*_7,9_ = 1.3, H7-dibenzofuryl); 7.56 (ddd, 1H, *J*_6,7_ = 8.2, *J*_6,8_ = 1.0, *J*_6,9_ = 0.7, H6-dibenzofuryl); 7.74 (dd, 1H, *J*_1,2_ = 7.7, *J*_1,3_ = 1.3, H1-dibenzofuryl); 7.92 (ddd, 1H, *J*_9,8_ = 7.7, *J*_9,7_ = 1.3, *J*_9,6_ = 0.7, H9-dibenzofuryl); 8.01 (s, 1H, H2-A). ^13^C NMR (150.9 MHz, D_2_O): 62.76 (C5′-G); 67.72 (d, *J*_C,P_ = 4.1, C5′-A); 71.28 (C3′-G); 79.98 (d, *J*_C,P_ = 5.4, C2′-G); 80.78 (d, *J*_C,P_ = 1.5, C3′-A); 84.00 (d, *J*_C,P_ = 1.1, C2′-A); 85.52 (C4′-G); 86.02 (d, *J*_C,P_ = 13.6, C4′-A); 89.95 (d, *J*_C,P_ = 6.7, C1′-G); 90.99 (d, *J*_C,P_ = 2.8, C1′-A); 103.36 (C4a-A); 114.12 (C5-A); 114.35 (C6-dibenzofuryl); 118.64 (C5-G); 119.57 (C4-dibenzofuryl); 122.29 (C1-dibenzofuryl); 123.51 (C9-dibenzofuryl); 124.43 (C6-A); 125.85 (C8-dibenzofuryl); 126.16 (C2-dibenzofuryl); 126.20 (C9a-dibenzofuryl); 127.03 (C9b-dibenzofuryl); 129.87 (C3-dibenzofuryl); 130.34 (C7-dibenzofuryl); 139.26 (C8-G); 152.46 (C7a-A); 152.86 (C4-G); 154.06 (C2-A); 154.96 (C4a-dibenzofuryl); 155.58 (C2-G); 158.12 (C5a-dibenzofuryl); 159.44 (C4-A); 160.77 (C6-G). ^31^P NMR (^1^H-dec, 202.4 MHz, D_2_O): 0.66 and 20.92. ESI MS *m*/*z* (rel. %): 418 (100) [M–2H]^2–^, 838 (12) [M–H]^−^, 860 (17) [M–2H + Na]^−^, 882 (4) [M–3H+2Na]^−^ HR MS (ESI): for C_33_H_30_O_14_N_9_P_2_ [M–H]^−^, calcd 838.13929; found, 838.13853.

##### Cyclo-4-amino-5-phenyl-7-β-d-ribofuranosyl-7*H*-pyrrolo[2,3-*d*]pyrimidine 5′-*O*-phosphate (3′ → 5′) 2-Amino-5-phenyl-7-β-d-ribofuranosyl-7*H*-pyrrolo[2,3-*d*]pyrimidin-4(3*H*)-one 5′-*O*-phosphate (2′ → 5′) Sodium Salt (**6f**)

NTPs **2f** (2 μmol) and **3f** (2 μmol) were enzymatically cyclized using mcGAS. The final product was purified by HPLC (5–30% MeCN in 0.1 M TEAB). The conversion to a sodium salt form on a Dowex 50WX8 (in a Na^+^ cycle) provided CDN **6f** (128 nmol, 6%). ^1^H NMR (600 MHz, D_2_O): 4.14 (ddd, 1H, *J*_5′b,4′_ = 1.2, *J*_5′b,5′a_ = 11.6, *J*_5′b,P_ = 3.0, H5′b-G); 4.26 (ddd, 1H, *J*_5′a,4′_ = 2.1, *J*_5′a,5′b_ = 11.6, *J*_5′a,P_ = 2.7, H5′a-G); 4.36 (ddd, 1H, *J*_5′b,4′_ = 1.2, *J*_5′b,5′a_ = 12.2, *J*_5′b,P_ = 3.2, H5′b-A); 4.46 (m, 1H, H4′-G); 4.475 (dm, 1H, *J*_4′,3′_ = 9.4, H4′-A); 4.52 (d, *J*_2′,3′_ = 4.2, H2′-A); 4.53 (m, 1H, H5′a-A); 4.76 (d, 1H, *J*_3′,4′_ = 3.8, H3′-G); 4.99 (ddd, 1H, *J*_3′,2′_ = 4.2, *J*_3′,4′_ = 9.4, *J*_3′,P_ = 7.2, H3′-A); 5.28 (um, 1H, H2′-G); 6.22 (s, 1H, H1′-A); 6.34 (d, 1H, *J*_1′,2′_ = 8.4, H1′-G); 6.98 (m, 3H, *m*- and *p*-Ph-G); 7.11 (m, 2H, *o*-Ph-A); 7.26 (m, 3H, *m*- and *p*-Ph-A); 7.31 (m, 2H, *o*-Ph-G); 7.37 (s, 1H, H6-G); 7.54 (s, 1H, H6-A); 7.98 (s, 1H, H-2-A). ^13^C NMR (150.9 MHz, D_2_O): 66.04 (C5′-A); 69.04 (C5′-G); 73.11 (C3′-A); 74.29 (C3′-G); 77.29 (C2′-A); 81.50 (C2′-G); 82.55 (C4′-A); 85.90 (C1′-G); 86.40 (C4′-G); 92.42 (C1′-A); 104.12 (C5-A); 117.34 (C6-G); 119.53 (C4a-A); 122.67 (C6-A); 123.20 (*p*-Ph-G); 124.93 (C4a-G); 129.29 (*o*-Ph-G); 129.85 (*o*-Ph-A); 130.10 (*m*-Ph-G); 131.27 (*m*-Ph and *p*-Ph-A); 151.02 (C7a-A); 153.39 (C2-A); 155.51 (C7a-G); 160.00 (C4-A); 163.10 (C4-G). ^31^P NMR (^1^H-dec, 202.4 MHz, D_2_O): 0.28 and −1.33. ESI MS *m*/*z* (rel. %): 411 (100) [M–2H]^2–^, 422 (13) [M–3H + Na]^2–^, 823 (11) [M–H]^−^, 845 (19) [M–2H + Na]^−^. HR MS (ESI): for C_34_H_33_N_8_O_13_P_2_ [M–H]^−^, calcd 823.16478; found, 823.16454.

##### Cyclo-4-amino-5-iodo-7-β-d-ribofuranosyl-7*H*-pyrrolo[2,3-*d*]pyrimidine 5′-*O*-phosphate (3′ → 5′) Guanosine 5′-*O*-phosphate (3′ → 5′) Sodium Salt (**7a**)

NTP **3a** (13 μmol) and GTP (13 μmol) were enzymatically cyclized using DncV. The final product was purified by HPLC (5–30% MeCN in 0.1 M TEAB). The conversion to a sodium salt form on a Dowex 50WX8 (in a Na^+^ cycle) provided CDN **7a** (5.3 mg, 48%) as a white lyophilizate (water). ^1^H NMR (600 MHz, D_2_O): 4.11 (m, H5′b-A); 4.12 (m, 1H, H5′b-G); 4.36 (m, 1H, H5′a-A); 4.39 (m, 1H, H4′-A); 4.42 (m, 2H, H4′-G + H5′a-G); 4.55 (dd, 1H, *J*_2′,1′_ = 1.0, *J*_2′,3′_ = 4.6, H2′-A); 4.73 (overlap water, H3′-A); 4.79 (overlap water, H2′-G); 4.85 (m, 1H, H3′-G); 6.01 (d, 1H, *J*_1′,2′_ = 1.0, H1′-G); 6.24 (d, 1H, *J*_1′,2′_ = 1.0, H1′-A); 7.75 (s, 1H, H6-A); 8.05 (s, 1H, H8-G); 8.10 (s, 1H, H2-A). ^13^C NMR (150.9 MHz, D_2_O): 53.44 (C5-A); 64.77 (d, *J*_C,P_ = 5.0, C5′-A); 64.92 (d, *J*_C,P_ = 4.9, C5′-G); 73.32 (d, *J*_C,P_ = 4.8, C3′-A); 73.34 (d, *J*_C,P_ = 4.8, C3′-G); 76.57 (C2′-A); 76.74 (C2′-G); 81.85 (t, *J*_C,P1_ = *J*_C,P2_ = 10.4, C4′-A); 82.43 (t, *J*_C,P1_ = *J*_C,P2_ = 10.8, C4′-G); 91.90 (C1′-A); 92.86 (d, *J*_C,P_ = 12.0, C1′-G); 106.75 (C4a-A); 119.66 (C5-G); 129.22 (C6-A); 139.65 (C8-G); 151.10 (C7a-A); 153.43 (C4-G); 154.33 (C2-A); 158.62 (C2-G); 159.91 (C4-A); 161.62 (C6-G). ^31^P NMR (^1^H-dec, 202.4 MHz, D_2_O): −0.56 and −0.89. ESI MS *m*/*z* (rel. %): 398 (100) [M–2H]^2–^, 798 (45) [M–H]^−^, 820 (27) [M–2H + Na]^−^. HR MS (ESI): for C_21_H_22_O_13_N_9_IP_2_ [M–2H]^2–^, calcd 398.49340; found, 398.49324.

##### Cyclo-4-amino-5-phenyl-7-β-d-ribofuranosyl-7*H*-pyrrolo[2,3-*d*]pyrimidine 5′-*O*-phosphate (3′ → 5′) Guanosine 5′-*O*-phosphate (3′ → 5′) Sodium Salt (**7f**)

CDN **7f** was prepared as described for **5g** from iodinated CDN **7a** (13 mg, 15.4 μmol) and phenylboronic acid (9.4 mg, 77.1 μmol). The final product was purified using HPLC (0–15% MeCN in 0.1 M TEAB). The conversion to a sodium salt form on a Dowex 50WX8 (in a Na^+^ cycle) provided CDN **7f** (8.1 mg, 66%) as a white lyophilizate (water). ^1^H NMR (600 MHz, D_2_O): 4.10 (ddd, 1H, *J*_5′b,4′_ = 0.9, *J*_5′b,5′a_ = 12.0, *J*_5′b,P_ = 4.4, H5′b-A); 4.13 (dd, 1H, *J*_5′b,5′a_ = 11.0, *J*_5′b,P_ = 4.3, H5′b-G); 4.37 (bdd, 1H, *J*_5′a,4′_ = 2.5, *J*_5′a,5′b_ = 12.0, *J*_5′a,P_ = <1, H5′a-A); 4.41 (m, 1H, H4′-A); 4.43 (m, 1H, H4′-G); 4.44 (dd, 1H, *J*_5′a,5′b_ = 11.0, *J*_5′a,P_ = 2.8, H5′a-G); 4.52 (d, 1H, *J*_2′,3′_ = 4.4, H2′-G); 4.61 (d, 1H, *J*_2′,3′_ = 4.4, H2′-A); 4.78 (overlap, H3′-A); 4.80 (overlap, 1H, H3′-G); 5.98 (s, 1H, H1′-G); 6.42 (s, 1H, H1′-A); 7.18 (m, 3H, 2× *m*-Ph + *p*-Ph); 7.28 (m, 2H, 2× *o*-Ph); 7.47 (s, 1H, H6-A); 8.03 (s, 1H, H8-G); 8.18 (s, 1H, H2-A). ^13^C NMR (150.9 MHz, D_2_O): 64.73 (d, *J*_C,P_ = 5.9, C5′-A); 64.77 (d, *J*_C,P_ = 4.7, C5′-G); 72.94 (d, *J*_C,P_ = 4.8, C3′-A); 73.55 (d, *J*_C,P_ = 5.0, C3′-G); 76.74 (C2′-A); 76.86 (C2′-G); 81.62 (t, *J*_C,P1_ = *J*_C,P2_ = 10.8, C4′-A); 82.11 (t, *J*_C,P_ = 11.5, C4′-G); 91.51 (C1′-A); 92.46 (C1′-G); 103.77 (C4a-A); 119.48 (C5-G); 120.80 (C5-A); 122.27 (C6-A); 129.37 (*p*-Ph); 130.68 (2× *o*-Ph); 131.16 (2× *m*-Ph); 135.81 (*i*-Ph); 138.74 (C8-G); 152.17 (C7a-A); 152.78 (C4-G); 154.17 (C2-A); 156.67 (C2-G); 160.35 (C4-A); 161.40 (C6-G). ^31^P NMR (^1^H-dec, 202.4 MHz, D_2_O): −0.36 and −0.75. ESI MS *m*/*z* (rel. %): 373 (100) [M–2H]^2–^, 748 (6) [M–H]^−^, 770 (5) [M–2H + Na]^−^. HR MS (ESI): for C_27_H_28_O_13_N_9_P_2_ [M–H]^−^, calcd 748.12873; found, 748.12726.

##### Cyclic-di-4-amino-5-iodo-7-β-d-ribofuranosyl-7*H*-pyrrolo[2,3-*d*]pyrimidine 5′-*O*-phosphate (3′ → 5′) Sodium Salt (**8a**)

NTP **3a** (26 μmol) was enzymatically cyclized using DisA. The final product was purified by HPLC (5–30% MeCN in 0.1 M TEAB). The conversion to a sodium salt form on a Dowex 50WX8 (in a Na^+^ cycle) provided CDN **8a** (7.0 mg, 57%) as a white lyophilizate (water). ^1^H NMR (600 MHz, D_2_O): 4.15 (dd, 1H, *J*_5′b,5′a_ = 11.7, *J*_5′b,P_ = 5.1, H5′b-A); 4.42 (dt, 1H, *J*_4′,3′_ = 9.8, *J*_4′,5′a_ = 2.7, *J*_3′,P_ = 2.8, H4′-A); 4.47 (bdd, 1H, *J*_5′a,4′_ = 2.7, *J*_5′a,5′b_ = 11.7, *J*_5′a,P_ < 1, H5′a-A); 4.60 (ddd, 1H, *J*_3′,2′_ = 4.0, *J*_3′,4′_ = 9.8, *J*_3′,P_ = 6.2, H3′-A); 4.69 (d, 1H, *J*_2′,3′_ = 4.0, H2′-A); 6.28 (s, 1H, H1′-A); 7.91 (s, 1H, H6-A); 8.12 (s, 1H, H2-A). ^13^C NMR (150.9 MHz, D_2_O): 52.38 (C5-A); 64.69 (d, *J*_C,P_ = 5.1, C5′-A); 73.45 (d, *J*_C,P_ = 5.3, C3′-A); 77.15 (C2′-A); 81.80 (t, *J*_C,P1_ = *J*_C,P2_ = 10.9, C4′-A); 92.67 (C1′-A); 107.78 (C4a-A); 128.45 (C6-A); 150.57 (C7a-A); 154.11 (C2-A); 160.03 (C4-A). ^31^P NMR (^1^H-dec, 202.4 MHz, D_2_O): −2.00. ESI MS *m*/*z* (rel. %): 452 (100) [M–2H]^2–^, 906 (28) [M–H]^−^, 928 (35) [M–2H + Na]^−^. HR MS (ESI): for C_22_H_23_O_12_N_8_I_2_P_2_ [M–H]^−^, calcd 906.90055; found, 906.90021.

##### Cyclic-di-4-amino-5-phenyl-7-β-d-ribofuranosyl-7*H*-pyrrolo[2,3-*d*]pyrimidine 5′-*O*-phosphate (3′ → 5′) Sodium Salt (**8f**)

CDN **8a** (15 mg, 15.8 μmol), phenylboronic acid (19.2 mg, 157.5 μmol), and cesium carbonate (30.8 mg, 94.5 μmol) were mixed with MeCN–H_2_O (1:2 v/v, 1.065 mL) in an argon-purged vial. In a separate vial, Pd(OAc)_2_ (1.0 mg, 4.45 μmol) and TPPTS (12.6 mg, 22.2 μmol) were dissolved in MeCN–H_2_O (1:2 v/v, 1.0 mL), and the solution was sonicated under argon atmosphere for 30 s. Then, 353 μmol of this solution was transferred into the mixture containing CDN **8a**, and the reaction was stirred at 100 °C for 30 min. Then, the reaction mixture was cooled to rt, diluted with water to approx. 3 mL, and filtered through a 5 μm nylon syringe filter. The filtrate was directly applied on HPLC for purification (0–17.5% MeCN in 0.1 M TEAB). The conversion to a sodium salt form on a Dowex 50WX8 (in a Na^+^ cycle) provided CDN **8f** (10.5 mg, 78%) as a white lyophilizate (water). ^1^H NMR (600 MHz, D_2_O): 4.18 (ddd, 1H, *J*_5′b,5′a_ = 11.3, *J*_5′b,4′_ = 1.5, *J*_5′b,P_ = 4.8, H5′b-A); 4.28 (d, 1H, *J*_2′,3′_ = 3.9, H2′-A); 4.45 (m, 2H, H4′-A + H5′a-A); 4.79 (overlap, H3′-A); 6.37 (s, 1H, H1′-A); 6.90 (m, 4H, *o*-Ph + *m*-Ph); 7.04 (m, 1H, *p*-Ph); 7.61 (s, 1H, H6-A); 8.16 (s, 1H, H2-A). ^13^C NMR (150.9 MHz, D_2_O): 64.78 (d, *J*_C,P_ = 5.0, C5′-A); 73.41 (d, *J*_C,P_ = 5.1, C3′-A); 77.32 (C2′-A); 81.62 (t, *J*_C,P_ = 10.9, C4′-A); 91.84 (C1′-A); 103.96 (C4a-A); 120.61 (C5-A); 121.74 (C6-A); 129.62 (*p*-Ph); 130.32 (*o*-Ph); 130.85 (*m*-Ph); 135.28 (*i*-Ph); 151.20 (C7a-A); 153.87 (C2-A); 159.85 (C4-A). ^31^P NMR (^1^H-dec, 202.4 MHz, D_2_O): −0.84. ESI MS *m*/*z* (rel. %): 403 (100) [M–2H]^2–^, 807 (3) [M–H]^−^, 829 (4) [M–2H + Na]^−^. HR MS (ESI): for C_34_H_33_O_12_N_8_P_2_ [M–H]^−^, calcd 807.16986; found, 807.16888.

##### 4-Benzamido-5-iodo-7-[5-*O*-(4,4′-dimethoxytrityl)-β-d-ribofuranosyl]-7*H*-pyrrolo[2,3-*d*]pyrimidine (**10**)

7-Iodo-7-deazaadenosine (**9**, ref (55), 500 mg, 1.28 mmol) was co-evaporated with anhydrous pyridine (2 × 3 mL) and suspended in anhydrous pyridine (5 mL). The suspension was cooled to 0 °C, and trimethylsilyl chloride (728 μL, 5.74 mmol) was added dropwise over a period of 30 min. Then, benzoyl chloride (222 μL, 1.91 mmol) was added, and the mixture was stirred at rt overnight. The reaction mixture was then cooled to 0 °C, and water (1.25 mL) was added. After stirring for 15 min, aqueous ammonia (25%, 2.5 mL) was added, and the stirring was continued for another 30 min. Solvents were then evaporated, and the residue was co-evaporated with anhydrous pyridine (2 × 3 mL) and dissolved in anhydrous pyridine (5 mL). DMTrCl (432 mg, 1.25 mmol) was added in two portions over 1 h, and the reaction mixture was stirred overnight. Then, DMTrCl (432 mg, 1.25 mmol) was added again in two portions over 1 h, and the reaction mixture was stirred for another 1.5 h. The solvent was removed in vacuo, the residue was suspended in DCM (35 mL), and the solid phase was filtered off through a pad of Celite. The filtrate was washed with saturated aqueous NaHCO_3_, dried over magnesium sulfate, and evaporated. Flash chromatography on silica (0–10% MeOH in DCM + 1% Et_3_N) provided nucleoside **10** (689 mg, 68%) as a white amorphous solid. ^1^H NMR (500.0 MHz, CD_3_OD): 3.39 (dd, 1H, *J*_gem_ = 10.7, *J*_5′b,4′_ = 2.9, H-5′b); 3.42 (dd, 1H, *J*_gem_ = 10.7, *J*_5′a,4′_ = 3.7, H-5′a); 3.757, 3.760 (2 × s, 2 × 3H, CH_3_O-DMTr); 4.19 (ddd, 1H, *J*_4′,3′_ = 4.1, *J*_4′,5a′_ = 3.7, *J*_4′,5b′_ = 2.9, H-4′); 4.46 (dd, 1H, *J*_3′,2′_ = 5.0, *J*_3′,4′_ = 4.1, H-3′); 4.72 (dd, 1H, *J*_2′,1′_ = 5.4, *J*_2′,3′_ = 5.0, H-2′); 6.37 (d, 1H, *J*_1′,2′_ = 5.4, H-1′); 6.81–6.88 (m, 4H, H-*m*-C_6_H_4_-DMTr); 7.21 (m, 1H, H-*p*-C_6_H_5_-DMTr); 7.25–7.32 (m, 2H, H-*m*-C_6_H_5_-DMTr); 7.30–7.37 (m, 8H, H-*o*-C_6_H_4_-DMTr); 7.42–7.48 (m, 2H, H-*o*-C_6_H_5_-DMTr); 7.54–7.59 (m, 2H, H-*m*-Bz); 7.64 (m, 1H, H-*p*-Bz); 7.86 (s, 1H, H-6); 8.08–8.12 (m, 2H, H-*o*-Bz); 8.65 (s, 1H, H-2). ^13^C NMR (125.7 MHz, CD_3_OD): 52.84 (C-5); 55.75, 55.76 (CH_3_O-DMTr); 64.74 (CH_2_-5′); 72.42 (CH-3′); 76.35 (CH-2′); 85.43 (CH-4′); 88.01 (C-DMTr); 89.41 (CH-1′); 114.23 (CH-*m*-C_6_H_4_-DMTr); 114.68 (C-4a); 127.99 (CH-*p*-C_6_H_5_-DMTr); 128.96 (CH-*m*-C_6_H_5_-DMTr); 129.23 (CH-*o*-Bz); 129.41 (CH-*o*-C_6_H_5_-DMTr); 129.90 (CH-*m*-Bz); 131.29, 131.40 (CH-*o*-C_6_H_4_-DMTr); 133.30 (CH-6); 133.78 (CH-*p*-Bz); 134.94 (C-*i*-Bz); 136.87, 137.15 (C-*i*-C_6_H_4_-DMTr); 146.04 (C-*i*-C_6_H_5_-DMTr); 152.24 (CH-2); 152.41 (C-4); 153.87 (C-7a); 160.17, 160.19 (C-*p*-C_6_H_4_-DMTr); 169.21 (C-*i*-Bz). ESI MS *m/z* (rel. %): 799 (23) [M + H]^+^, 821 (100) [M + Na]^+^, 837 (9) [M + K]^+^. HR MS (ESI): for C_39_H_36_O_7_N_4_I [M + H]^+^, calcd 799.16232; found, 799.16154.

##### 4-Benzamido-5-iodo-7-[2-*O*-*tert*-butyldimethylsilyl-5-*O*-(4,4′-dimethoxytrityl)-β-d-ribofuranosyl]-7*H*-pyrrolo[2,3-*d*]pyrimidine (**11**)

Nucleoside **10** (555 mg, 0.70 mmol) was co-evaporated with anhydrous pyridine (2 × 5 mL) and dissolved in anhydrous THF (10 mL). Anhydrous pyridine (485 μL) and silver nitrate (195 mg, 1.15 mmol) were added. The reaction flask was covered with aluminum foil, and the mixture was stirred at rt for 15 min. Then, TBDMSCl (183 mg, 1.21 mmol) was added, and the mixture was stirred overnight in the dark. The reaction mixture was then filtered through a pad of Celite. The filtration pad was washed with DCM, and the filtrate was evaporated. Flash chromatography on silica (5–50% EtOAc + 1% Et_3_N in cyclohexane) provided nucleoside **11** (251 mg, 40%) as a white foam. ^1^H NMR (500.0 MHz, CD_3_OD): −0.13, 0.01 (2 × s, 2 × 3H, CH_3_Si); 0.81 (s, 9H, (CH_3_)_3_C); 3.43 (dd, 1H, *J*_gem_ = 10.7, *J*_5′b,4′_ = 3.0, H-5′b); 3.46 (dd, 1H, *J*_gem_ = 10.7, *J*_5′a,4′_ = 2.7, H-5′a); 3.779, 3.782 (2 × s, 2 × 3H, CH_3_O-DMTr); 4.29 (ddd, 1H, *J*_4′,3′_ = 3.1, *J*_4′,5′a_ = 2.7, *J*_4′,5′b_ = 3.0, H-4′); 4.38 (dd, 1H, *J*_3′,2′_ = 5.0, *J*_3′,4′_ = 3.1, H-3′); 4.80 (dd, 1H, *J*_2′,1′_ = 5.9, *J*_2′,3′_ = 5.0, H-2′); 6.39 (d, 1H, *J*_1′,2′_ = 5.9, H-1′); 6.85–6.90 (m, 4H, H-*m*-C_6_H_4_-DMTr); 7.24 (m, 1H, H-*p*-C_6_H_5_-DMTr); 7.28–7.33 (m, 2H, H-*m*-C_6_H_5_-DMTr); 7.34–7.39 (m, 8H, H-*o*-C_6_H_4_-DMTr); 7.45–7.49 (m, 2H, H-*o*-C_6_H_5_-DMTr); 7.55–7.60 (m, 2H, H-*m*-Bz); 7.66 (m, 1H, H-*p*-Bz); 7.91 (s, 1H, H-6); 8.09–8.13 (m, 2H, H-*o*-Bz); 8.65 (s, 1H, H-2). ^13^C NMR (125.7 MHz, CD_3_OD): −4.94, −4.68 (CH_3_Si); 18.99 ((CH_3_)_3_**C**); 26.15 ((**C**H_3_)_3_C); 53.05 (C-5); 55.80 (CH_3_O-DMTr); 64.80 (CH_2_-5′); 72.98 (CH-3′); 78.48 (CH-2′); 86.02 (CH-4′); 88.27 (C-DMTr); 89.24 (CH-1′); 114.32 (CH-*m*-C_6_H_4_-DMTr); 114.57 (C-4a); 128.11 (CH-*p*-C_6_H_5_-DMTr); 129.05 (CH-*m*-C_6_H_5_-DMTr); 129.23 (CH-*o*-Bz); 129.35 (CH-*o*-C_6_H_5_-DMTr); 129.93 (CH-*m*-Bz); 131.33, 131.43 (CH-*o*-C_6_H_4_-DMTr); 133.16 (CH-6); 133.82 (CH-*p*-Bz); 134.94 (C-*i*-Bz); 136.74, 136.99 (C-*i*-C_6_H_4_-DMTr); 145.99 (C-*i*-C_6_H_5_-DMTr); 152.29 (CH-2); 152.55 (C-4); 153.92 (C-7a); 160.25, 160.26 (C-*p*-C_6_H_4_-DMTr); 169.22 (C-*i*-Bz). ESI MS *m/z* (rel. %): 913 (100) [M + H]^+^. HR MS (ESI): for C_45_H_50_O_7_N_4_ISi [M + H]^+^, calcd 913.24880; found, 913.24853.

##### 4-Benzamido-5-iodo-7-[2-*O*-*tert*-butyldimethylsilyl-β-d-ribofuranosyl]-7*H*-pyrrolo[2,3-*d*]pyrimidine 3′-*O*-Phosphonate Triethylammonium Salt (12)

Nucleoside **11** (525 mg, 0.58 mmol) was co-evaporated with anhydrous pyridine (2 × 5 mL) and dissolved in anhydrous pyridine (5 mL), and diphenyl phosphite (216 μL, 1.44 mmol) was added. The reaction mixture was stirred for 1 h, and then, water (8 mL) was added. The reaction mixture was stirred for 5 min, and it was then diluted with EtOAc and washed with brine. Aqueous phase was extracted twice with EtOAc. Combined organic phases were dried over MgSO_4_ and evaporated. The residue was dissolved in DCM (7 mL), and then, water (104 μL) and a solution of dichloroacetic acid in DCM (6%, 6.58 mL, 5.0 mmol) were added. The solution was stirred at rt for 15 min, and then, triethylsilane (919 μL, 5.75 mmol) was added. The reaction mixture was stirred for another 30 min. The reaction was quenched with pyridine (823 μL), and solvents were removed in vacuo. Flash chromatography on C18 column (gradient 5–100% MeCN in 0.1 M TEAB) provided phosphonate **12** (354 mg, 77%) as a white foam. ^1^H NMR (500.0 MHz, CD_3_OD): −0.24, 0.01 (2 × s, 2 × 3H, CH_3_Si); 0.77 (s, 9H, (CH_3_)_3_C); 1.31 (t, 9H, *J*_vic_ = 7.3, C**H**_**3**_CH_2_N); 3.20 (q, 6H, *J*_vic_ = 7.3, CH_3_C**H**_**2**_N); 3.87 (d, 2H, *J*_5′,4′_ = 2.6, H-5′); 4.33 (q, 1H, *J*_4′,3′_ = *J*_4′,5′_ = 2.6, H-4′); 4.73–4.79 (m, 2H, H-2′,3′); 6.33 (d, 1H, *J*_1′,2′_ = 6.1, H-1′); 6.96 (d, 1H, *J*_H,P_ = 630.1, HP); 7.54–7.59 (m, 2H, H-*m*-Bz); 7.65 (m, 1H, H-*p*-Bz); 8.07 (s, 1H, H-6); 8.10–8.15 (m, 2H, H-*o*-Bz); 8.67 (s, 1H, H-2). ^13^C NMR (125.7 MHz, CD_3_OD): −5.22, −4.60 (CH_3_Si); 9.22 (**C**H_3_CH_2_N); 18.84 ((CH_3_)_3_**C**); 26.13 ((**C**H_3_)_3_C); 47.86 (CH_3_**C**H_2_N); 52.61 (C-5); 62.66 (CH_2_-5′); 75.43 (d, *J*_C,P_ = 5.1, CH-3′); 77.09 (d, *J*_C,P_ = 3.0, CH-2′); 87.07 (d, *J*_C,P_ = 3.4, CH-4′); 89.77 (CH-1′); 114.84 (C-4a); 129.24 (CH-*o*-Bz); 129.89 (CH-*m*-Bz); 133.74 (CH-*p*-Bz); 133.93 (CH-6); 135.03 (C-*i*-Bz); 152.05 (CH-2); 152.68 (C-4); 153.65 (C-7a); 169.10 (C-*i*-Bz). ^31^P{^1^H} NMR (202.4 MHz, CD_3_OD): 5.24. ESI MS *m/z* (rel. %): 673 (100) [M–H]^−^. HR MS (ESI): for C_24_H_31_O_7_N_4_IPSi [M–H]^−^, calcd 673.07498; found, 673.07515.

### Differential Scanning Fluorimetry

Measurements were performed with wt hSTING as described earlier.^47^ Briefly, thermal denaturation was performed in a 20 μL mixture consisting of 0.1 mg/mL wt hSTING, 150 μM CDN, 100 mM Tris–HCl [pH 7.4], 150 mM NaCl, and 1:500 (v/v) SYPRO Orange (Thermo Fisher Scientific, USA). Measurements were taken in a 96-well format on a LightCycler480 Instrument II (Roche, Switzerland). Thermal shift (Δ*T*_m_) for each CDN was determined by subtracting the melting temperature (*T*_m_) of the reference sample (ligand-free STING) from the melting temperature of the STING–CDN complex.

### Standard 293T Cell-Based Reporter Assay

The generation of 293T reporter cells expressing different full-length STING protein allelic variants and the assay procedure itself was described previously.^20^ Briefly, the day before testing, the cells were seeded into 96-well transparent plates in a DMEM High glucose medium containing 10% (v/v) heat inactivated fetal bovine albumin (FBS). The medium was removed the next day, and the cells were incubated for 7 h (37 °C, 5% CO_2_) with serially diluted compounds. After incubation, the collected cell culture medium was mixed with Bright-Glo Luciferase Assay System reagent (Promega, USA) in white 96-well plates (Corning, USA). Luminescence readout was performed on a SPARK instrument (Tecan, Austria), and the values were used to calculate EC_50_ values using nonlinear regression and software Prism (GraphPad, USA).

### Digitonin 293T Cell-Based Reporter Assay

Cells were plated as described above for a standard 293T cell-based reporter assay. The following day, the medium was removed, and serially diluted compounds were incubated with cells for 30 min at 37 °C, 5% CO_2_ in buffer containing 50 mM HEPES [pH 7.0], 100 mM KCl, 3 mM MgCl_2_, 0.1 mM DTT, 85 mM sucrose, 0.2% (w/w) BSA, 1 mM ATP, 0.1 mM GTP, and 10 μg/mL digitonin A. Next, the buffer was removed, and the cells were washed with a cell culture medium twice and then incubated for 5 h (37 °C, 5% CO_2_) with fresh medium. Finally, the levels of Lucia Luciferase in medium were determined using Bright Glo (Promega, USA), and EC_50_s were calculated as described above.

### PBMC Assay

Buffy coats from healthy individuals were obtained from the Institute of Hematology and Blood Transfusion (IHBT, Prague, Czech Republic). An informed written consent was obtained from each individual enrolled. The study was approved by the Institutional Review Board of IHBT (ev. nb. 13/06/2012). The assay was performed as previously described.^54^ Briefly, the isolation of PBMCs was performed using Ficoll density gradient centrifugation (Ficoll Paque Plus, GE Healthcare) in SepMate tubes (SepMate PBMC Isolation Tubes, Stemcell Technologies). Next, isolated PBMCs were seeded into a 96-well U-shape plate in RPMI 1640 media containing 10% (v/v) heat inactivated fetal bovine serum (Capricorn Scientific, Germany). Serially diluted CDNs were added, and the plates were incubated for a predetermined 16 h (37 °C, 5% CO_2_). Finally, secreted IFNα, IFNγ, and TNFα were determined in the collected medium using ProcartaPlex Assays using a MAGPIX System (Merck, Germany) according to manufacturer’s instruction. The cytotoxic effect of CDNs on PBMCs was determined using CellTiter-Glo Luminescent Cell Viability Assay (Promega Corporation, USA) according to manufacturer’s instruction.

### Protein Expression and Purification

The expression and purification of mouse cGAS were performed as previously described^20^ with minor changes. The recombinant plasmid was transformed in *Escherichia coli* BL21 (DE3) cells (Thermo Fisher, USA). The bacteria were grown at 37 °C until OD_600nm_ of 0.6–0.8 units and cooled down to 20 °C, and then, cGAS expression was induced with 0.4 mM IPTG for 16 h in an orbital shaker. After cell collection, the pellet was resuspended in ice-cold lysis/wash buffer (50 mM Tris–HCl [pH 8], 300 mM NaCl, 20 mM imidazole, 10% (w/v) glycerol, 3 mM β-mercaptoethanol). The cells were lysed by sonication, and cell debris was removed by centrifugation. The supernatant was incubated with equilibrated Ni-NTA beads (Macherey-Nagel, Germany). The beads were loaded into a gravity flow column and washed with lysis/wash buffer. Next, the resin was washed with lysis/wash buffer supplemented with NaCl to 1 M concentration, then washed with lysis/wash buffer. The mcGAS was eluted with lysis/wash buffer supplemented with imidazole to a 300 mM concentration. The collected protein was further purified on a size-exclusion chromatography column HiLoad 26/60 Superdex 200 pg in buffer containing 50 mM Tris–HCl [pH 7.4] and 150 mM NaCl. The eluate was concentrated to about 5 mg/mL and stored in 20% glycerol at −80 °C.

Expression and purification of DncV and DisA proteins were performed as described previously.^33^ Briefly, proteins were overexpressed in *E. coli* BL21 (DE3) cells (ThermoFisher, USA). Purification steps consisted of cell disintegration, Ni-NTA chromatography, and SEC chromatography using HiLoad 26/60 Superdex 200 pg. The DncV and DisA proteins were concentrated using an Amicon Ultra-15 10K device (Merck Millipore Ltd), and enzymes were flash-frozen in liquid nitrogen.

The expression and purification of wt hSTING were performed as described previously.^47^ Briefly, the expression, lysis, and purification using Ni-NTA resin were done following mcGAS protocol. Eluate from the Ni-NTA resin was supplied with Ulp1 protease and incubated for 2 h at 4 °C. Digested wt hSTING protein was further purified by size exclusion with HiLoad 26/60 Superdex 200 pg (GE Healthcare, USA) equilibrated in buffer containing 50 mM Tris–HCl [pH 7.4] and 150 mM NaCl. Finally, the purified protein was concentrated to about 5 mg/mL and stored in 20% glycerol at −80 °C, for usage in DSF. For the crystallography experiments, wt hSTING was further purified on HiTrap Capto S (GE Healthcare, USA), equilibrated in 50 mM Tris–HCl [pH 8] buffer, and eluted with a gradient of 50–1000 mM NaCl in the same buffer. Fractions corresponding to wt hSTING were concentrated to about 20 mg/mL and stored at −80 °C without glycerol supplement.

### Crystallization and Structure Determination

Crystallizations were done using our previously optimized protocol.^52^ wt hSTING-**5f** was co-crystalized at 18 °C using sitting drop vapor diffusion in a condition composed of 200 mM ammonium nitrate, 20% (w/v) PEG 3350, with additive 10 mM EDTA. Crystals were cryoprotected in mother liquor supplemented with 20% (v/v) glycerol. A complete diffraction data set was collected for a single crystal to 2.69 Å. wt hSTING-**5k** was co-crystalized at 18 °C using sitting drop vapor diffusion in a condition composed of 100 mM tri-sodium citrate [pH 5.0] and 20% (w/v) PEG 6000, with additives of 10 mM EDTA and 100 mM tri-sodium citrate. Crystals were cryoprotected in mother liquor supplemented with 20% (v/v) ethylene glycol. A complete diffraction data set was collected for a single crystal to 2.32 Å. wt hSTING-**5l** was co-crystalized at 18 °C using sitting drop vapor diffusion in a condition composed of 100 mM citric acid, 20% (w/v) PEG 6000, with additive 10 mM EDTA. Crystals were cryoprotected in mother liquor supplemented with 20% (v/v) ethylene glycol. A complete diffraction data set was collected for a single crystal to 2.16 Å. wt hSTING-**5m** was co-crystalized at 18 °C using sitting drop vapor diffusion in a condition composed of 100 mM PIPES [pH 7.0], 100 mM magnesium formate dihydrate, 100 mM rubidium chloride, 25% (w/v) PEG smear high, with additives 10 mM EDTA and 100 mM tri-sodium citrate. Crystals were cryoprotected in mother liquor supplemented with 20% (v/v) ethylene glycol. A complete diffraction data set was collected for a single crystal to 1.89 Å. Measurements for hSTING-**5f** and hSTING-**5k** were carried out at an in-house diffractometer (Rigaku, Japan) at 100 K. Measurements for hSTING-**5l** and hSTING-**5m** were carried out at MX14.1 operated by the Helmholtz-Zentrum Berlin (HZB) at the BESSY II electron storage ring (Berlin-Adlershof, Germany) at 100 K.^56^ Data were reduced and processed by *XDS*.^57^ The structures were solved by the molecular replacement method with *MOLREP*(58) using the structure of human STING (PDB entry 4KSY) as the search model. Model refinement was done using *REFMAC* from *CCP*4 *suite*,^59^*PHENIX,*^60^ and *Coot*.^61^ Parameters for structures and their geometry are summarized in Table S1.

## Supporting Information Available

Synthesis of compound **3e**, crystal data and diffraction data collection and refinement statistics, UPLC purity of CDNs including UPLC traces, top signals and CC_50_s for compounds tested on PBMCs, 2*Fo*–*Fc* map of loops over ligand-binding sites, and interaction scheme of 2′3′-*c*GAMP in STING ligand-binding sites (PDF)
Molecular formula strings (CSV)

## Accession Codes

PDB codes for human STING with bound **5f** (8A2H), **5k** (8A2J), **5l** (8A2I), and **5m** (8A2K). Authors will release the atomic coordinates upon article publication.

## Abbreviations

CDN: cyclic dinucleotide
cGAS: cyclic GMP–AMP synthase
DAMP: damage-associated molecular pattern
DisA: diadenylate cyclase from *Bacillus thuringiensis*
DMOCP: 2-chloro-5,5-dimethyl-1,3,2-dioxaphosphorinane 2-oxide
DMTr: 4,4′-dimethoxytrityl
DncV: dinucleotide cyclase from *Vibrio cholerae*
DSF: differential scanning fluorimetry
ETT: ethylthio-1*H*-tetrazole
FBS: fetal bovine albumin
IRF: interferon regulatory factor
*J*: coupling constant in Hz
mcGAS: mouse variant of cyclic GMP–AMP synthase
NF-κB: nuclear factor κ-light-chain enhancer of activated B-cells
NMR: nuclear magnetic resonance
NTP: nucleoside triphosphate
PAMP: pathogen-associated molecular pattern
rt: room temperature
SAR: structure–activity relationship
STING: stimulator of interferon genes
TBAF: tetrabutylammonium fluoride
TFA: trifluoroacetic acid
TLC: thin layer chromatography
TPPTS: triphenylphosphan-3,3′,3″-trisulfonate
UV: ultraviolet
wt hSTING: wild type variant of human stimulator of interferon genes

## References

[ref1] Ishikawa H.; Barber G. N. STING is an endoplasmic reticulum adaptor that facilitates innate immune signalling. Nature 2008, 455, 674–678. 10.1038/nature07317. 18724357 PMC2804933

[ref2] Zhong B.; Yang Y.; Li S.; Wang Y.-Y.; Li Y.; Diao F.; Lei C.; He X.; Zhang L.; Tien P.; Shu H.-B. The adaptor protein MITA links virus-sensing receptors to IRF3 transcription factor activation. Immunity 2008, 29, 538–550. 10.1016/j.immuni.2008.09.003. 18818105

[ref3] Sun L.; Wu J.; Du F.; Chen X.; Chen Z. J. Cyclic GMP-AMP synthase is a cytosolic DNA sensor that activates the type I interferon pathway. Science 2013, 339, 786–791. 10.1126/science.1232458. 23258413 PMC3863629

[ref4] Wu J.; Sun L.; Chen X.; Du F.; Shi H.; Chen C.; Chen Z. J. Cyclic GMP-AMP is an endogenous second messenger in innate immune signaling by cytosolic DNA. Science 2013, 339, 826–830. 10.1126/science.1229963. 23258412 PMC3855410

[ref5] Zhang X.; Shi H.; Wu J.; Zhang X.; Sun L.; Chen C.; Chen Z. J. Cyclic GMP-AMP containing mixed phosphodiester linkages is an endogenous high-affinity ligand for STING. Mol. Cell 2013, 51, 226–235. 10.1016/j.molcel.2013.05.022. 23747010 PMC3808999

[ref6] Ablasser A.; Goldeck M.; Cavlar T.; Deimling T.; Witte G.; Röhl I.; Hopfner K.-P.; Ludwig J.; Hornung V. cGAS produces a 2′-5′-linked cyclic dinucleotide second messenger that activates STING. Nature 2013, 498, 380–384. 10.1038/nature12306. 23722158 PMC4143541

[ref7] Ouyang S.; Song X.; Wang Y.; Ru H.; Shaw N.; Jiang Y.; Niu F.; Zhu Y.; Qiu W.; Parvatiyar K.; Li Y.; Zhang R.; Cheng G.; Liu Z.-J. Structural analysis of the STING adaptor protein reveals a hydrophobic dimer interface and mode of cyclic di-GMP binding. Immunity 2012, 36, 1073–1086. 10.1016/j.immuni.2012.03.019. 22579474 PMC3654694

[ref8] Krasteva P. V.; Sondermann H. Versatile modes of cellular regulation via cyclic dinucleotides. Nat. Chem. Biol. 2017, 13, 350–359. 10.1038/nchembio.2337. 28328921 PMC5773344

[ref9] Burdette D. L.; Monroe K. M.; Sotelo-Troha K.; Iwig J. S.; Eckert B.; Hyodo M.; Hayakawa Y.; Vance R. E. STING is a direct innate immune sensor of cyclic di-GMP. Nature 2011, 478, 515–518. 10.1038/nature10429. 21947006 PMC3203314

[ref10] Barker J. R.; Koestler B. J.; Carpenter V. K.; Burdette D. L.; Waters C. M.; Vance R. E.; Valdivia R. H. STING-dependent recognition of cyclic di-AMP mediates type I interferon responses during Chlamydia trachomatis infection. mBio 2013, 4, e00018 10.1128/mBio.00018-13. 23631912 PMC3663186

[ref11] Keating S. E.; Baran M.; Bowie A. G. Cytosolic DNA sensors regulating type I interferon induction. Trends Immunol. 2011, 32, 574–581. 10.1016/j.it.2011.08.004. 21940216

[ref12] Liu S.; Cai X.; Wu J.; Cong Q.; Chen X.; Li T.; Du F.; Ren J.; Wu Y.-T.; Grishin N. V.; Chen Z. J. Phosphorylation of innate immune adaptor proteins MAVS, STING, and TRIF induces IRF3 activation. Science 2015, 347, aaa2630 10.1126/science.aaa2630. 25636800

[ref13] Shang G.; Zhang C.; Chen Z. J.; Bai X.-c.; Zhang X. Cryo-EM structures of STING reveal its mechanism of activation by cyclic GMP-AMP. Nature 2019, 567, 389–393. 10.1038/s41586-019-0998-5. 30842659 PMC6859894

[ref14] Zhang C.; Shang G.; Gui X.; Zhang X.; Bai X.-c.; Chen Z. J. Structural basis of STING binding with and phosphorylation by TBK1. Nature 2019, 567, 394–398. 10.1038/s41586-019-1000-2. 30842653 PMC6862768

[ref15] Zhao B.; Du F.; Xu P.; Shu C.; Sankaran B.; Bell S. L.; Liu M.; Lei Y.; Gao X.; Fu X.; Zhu F.; Liu Y.; Laganowsky A.; Zheng X.; Ji J.-Y.; West A. P.; Watson R. O.; Li P. A conserved PLPLRT/SD motif of STING mediates the recruitment and activation of TBK1. Nature 2019, 569, 718–722. 10.1038/s41586-019-1228-x. 31118511 PMC6596994

[ref16] Musella M.; Manic G.; De Maria R.; Vitale I.; Sistigu A. Type-I-interferons in infection and cancer: Unanticipated dynamics with therapeutic implications. OncoImmunology 2017, 6, e1314424 10.1080/2162402x.2017.1314424. 28638743 PMC5467995

[ref17] Barber G. N. STING: infection, inflammation and cancer. Nat. Rev. Immunol. 2015, 15, 760–770. 10.1038/nri3921. 26603901 PMC5004891

[ref18] Kumar V. A. A STING to inflammation and autoimmunity. J. Leukocyte Biol. 2019, 106, 171–185. 10.1002/jlb.4mir1018-397rr. 30990921

[ref19] Gao D.; Li T.; Li X.-D.; Chen X.; Li Q.-Z.; Wight-Carter M.; Chen Z. J. Activation of cyclic GMP-AMP synthase by self-DNA causes autoimmune diseases. Proc. Natl. Acad. Sci. U.S.A. 2015, 112, E5699 10.1073/pnas.1516465112. 26371324 PMC4620884

[ref20] Novotná B.; Vaneková L.; Zavřel M.; Buděšínský M.; Dejmek M.; Smola M.; Gutten O.; Tehrani Z. A.; Pimková Polidarová M.; Brázdová A.; Liboska R.; Štěpánek I.; Vavřina Z.; Jandušík T.; Nencka R.; Rulíšek L.; Bouřa E.; Brynda J.; Páv O.; Birkuš G. Enzymatic preparation of 2′–5′,3′–5′-cyclic dinucleotides, their binding properties to stimulator of interferon genes adaptor protein, and structure/activity correlations. J. Med. Chem. 2019, 62, 10676–10690. 10.1021/acs.jmedchem.9b01062. 31715099

[ref21] Zhang H.; You Q.-D.; Xu X.-L. Targeting stimulator of interferon genes (STING): A medicinal chemistry perspective. J. Med. Chem. 2020, 63, 3785–3816. 10.1021/acs.jmedchem.9b01039. 31820978

[ref22] Le Naour J.; Zitvogel L.; Galluzzi L.; Vacchelli E.; Kroemer G. Trial watch: STING agonists in cancer therapy. OncoImmunology 2020, 9, 1777624 10.1080/2162402x.2020.1777624. 32934881 PMC7466854

[ref23] Fu J.; Kanne D. B.; Leong M.; Glickman L. H.; McWhirter S. M.; Lemmens E.; Mechette K.; Leong J. J.; Lauer P.; Liu W.; Sivick K. E.; Zeng Q.; Soares K. C.; Zheng L.; Portnoy D. A.; Woodward J. J.; Pardoll D. M.; Dubensky T. W.; Kim Y. STING agonist formulated cancer vaccines can cure established tumors resistant to PD-1 blockade. Sci. Transl. Med. 2015, 7, 283ra52 10.1126/scitranslmed.aaa4306. PMC4504692 25877890

[ref24] Chang W.; Altman M. D.; Lesburg C. A.; Perera S. A.; Piesvaux J. A.; Schroeder G. K.; Wyss D. F.; Cemerski S.; Chen Y.; DiNunzio E.; Haidle A. M.; Ho T.; Kariv I.; Knemeyer I.; Kopinja J. E.; Lacey B. M.; Laskey J.; Lim J.; Long B. J.; Ma Y.; Maddess M. L.; Pan B.-S.; Presland J. P.; Spooner E.; Steinhuebel D.; Truong Q.; Zhang Z.; Fu J.; Addona G. H.; Northrup A. B.; Parmee E.; Tata J. R.; Bennett D. J.; Cumming J. N.; Siu T.; Trotter B. W. Discovery of MK-1454: A potent cyclic dinucleotide stimulator of interferon genes agonist for the treatment of cancer. J. Med. Chem. 2022, 65, 5675–5689. 10.1021/acs.jmedchem.1c02197. 35332774

[ref25] Carideo Cunniff E.; Sato Y.; Mai D.; Appleman V. A.; Iwasaki S.; Kolev V.; Matsuda A.; Shi J.; Mochizuki M.; Yoshikawa M.; Huang J.; Shen L.; Haridas S.; Shinde V.; Gemski C.; Roberts E. R.; Ghasemi O.; Bazzazi H.; Menon S.; Traore T.; Shi P.; Thelen T. D.; Conlon J.; Abu-Yousif A. O.; Arendt C.; Shaw M. H.; Okaniwa M. TAK-676: A novel stimulator of interferon genes (STING) agonist promoting durable IFN-dependent antitumor immunity in preclinical studies. Cancer Res. Commun. 2022, 2, 489–502. 10.1158/2767-9764.crc-21-0161. 36923556 PMC10010323

[ref26] Vyskocil S.; Cardin D.; Ciavarri J.; Conlon J.; Cullis C.; England D.; Gershman R.; Gigstad K.; Gipson K.; Gould A.; Greenspan P.; Griffin R.; Gulavita N.; Harrison S.; Hu Z.; Hu Y.; Hata A.; Huang J.; Huang S.-C.; Janowick D.; Jones M.; Kolev V.; Langston S. P.; Lee H. M.; Li G.; Lok D.; Ma L.; Mai D.; Malley J.; Matsuda A.; Mizutani H.; Mizutani M.; Molchanova N.; Nunes E.; Pusalkar S.; Renou C.; Rowland S.; Sato Y.; Shaw M.; Shen L.; Shi Z.; Skene R.; Soucy F.; Stroud S.; Xu H.; Xu T.; Abu-Yousif A. O.; Zhang J. Identification of novel carbocyclic pyrimidine cyclic dinucleotide STING agonists for antitumor immunotherapy using systemic intravenous route. J. Med. Chem. 2021, 64, 6902–6923. 10.1021/acs.jmedchem.1c00374. 34000802

[ref27] Ramanjulu J. M.; Pesiridis G. S.; Yang J.; Concha N.; Singhaus R.; Zhang S.-Y.; Tran J.-L.; Moore P.; Lehmann S.; Eberl H. C.; Muelbaier M.; Schneck J. L.; Clemens J.; Adam M.; Mehlmann J.; Romano J.; Morales A.; Kang J.; Leister L.; Graybill T. L.; Charnley A. K.; Ye G.; Nevins N.; Behnia K.; Wolf A. I.; Kasparcova V.; Nurse K.; Wang L.; Puhl Y.; Li M.; Klein C. B.; Hopson J.; Guss M.; Bantscheff G.; Bergamini M. A.; Reilly Y.; Lian K. J.; Duffy J.; Adams K. P.; Foley P. J.; Gough R. W.; Marquis J.; Smothers A.; Hoos J.; Bertin J. Design of amidobenzimidazole STING receptor agonists with systemic activity. Nature 2018, 564, 439–443. 10.1038/s41586-018-0705-y. 30405246

[ref28] Banerjee M.; Middya S.; Shrivastava R.; Basu S.; Ghosh R.; Pryde D. C.; Yadav D. B.; Surya A. G10 is a direct activator of human STING. PLoS One 2020, 15, e0237743 10.1371/journal.pone.0237743. 32911484 PMC7482845

[ref29] Sali T. M.; Pryke K. M.; Abraham J.; Liu A.; Archer I.; Broeckel R.; Staverosky J. A.; Smith J. L.; Al-Shammari A.; Amsler L.; Sheridan K.; Nilsen A.; Streblow D. N.; DeFilippis V. R. Characterization of a novel human-specific STING agonist that elicits antiviral activity against emerging alphaviruses. PLoS Pathog. 2015, 11, e1005324 10.1371/journal.ppat.1005324. 26646986 PMC4672893

[ref30] Pan B.-S.; Perera S. A.; Piesvaux J. A.; Presland J. P.; Schroeder G. K.; Cumming J. N.; Trotter B. W.; Altman M. D.; Buevich A. V.; Cash B.; Cemerski S.; Chang W.; Chen Y.; Dandliker P. J.; Feng G.; Haidle A.; Henderson T.; Jewell J.; Kariv I.; Knemeyer I.; Kopinja J.; Lacey B. M.; Laskey J.; Lesburg C. A.; Liang R.; Long B. J.; Lu M.; Ma Y.; Minnihan E. C.; O’Donnell G.; Otte R.; Price L.; Rakhilina L.; Sauvagnat B.; Sharma S.; Tyagarajan S.; Woo H.; Wyss D. F.; Xu S.; Bennett D. J.; Addona G. H. An orally available non-nucleotide STING agonist with antitumor activity. Science 2020, 369, eaba6098 10.1126/science.aba6098. 32820094

[ref31] Cherney E. C.; Zhang L.; Lo J.; Huynh T.; Wei D.; Ahuja V.; Quesnelle C.; Schieven G. L.; Futran A.; Locke G. A.; Lin Z.; Monereau L.; Chaudhry C.; Blum J.; Li S.; Fereshteh M.; Li-Wang B.; Gangwar S.; Pan C.; Chong C.; Zhu X.; Posy S. L.; Sack J. S.; Zhang P.; Ruzanov M.; Harner M.; Akhtar F.; Schroeder G. M.; Vite G.; Fink B. Discovery of non-nucleotide small-molecule STING agonists via chemotype hybridization. J. Med. Chem. 2022, 65, 3518–3538. 10.1021/acs.jmedchem.1c01986. 35108011

[ref32] Jeon M. J.; Lee H.; Lee J.; Baek S. Y.; Lee D.; Jo S.; Lee J.-Y.; Kang M.; Jung H. R.; Han S. B.; Kim N.-J.; Lee S.; Kim H. Development of potent immune modulators targeting stimulator of interferon genes receptor. J. Med. Chem. 2022, 65, 5407–5432. 10.1021/acs.jmedchem.1c01795. 35315650

[ref33] Novotná B.; Holá L.; Staś M.; Gutten O.; Smola M.; Zavřel M.; Vavřina Z.; Buděšínský M.; Liboska R.; Chevrier F.; Dobiaš J.; Boura E.; Rulíšek L.; Birkuš G. Enzymatic synthesis of 3′–5′, 3′–5′ cyclic dinucleotides, their binding properties to the stimulator of interferon genes adaptor protein, and structure/activity correlations. Biochemistry 2021, 60, 3714–3727. 10.1021/acs.biochem.1c00692. 34788017

[ref34] Rosenthal K.; Becker M.; Rolf J.; Siedentop R.; Hillen M.; Nett M.; Lütz S. Catalytic Promiscuity of cGAS: A Facile Enzymatic Synthesis of 2′-3′-Linked Cyclic Dinucleotides. ChemBioChem 2020, 21, 3225–3228. 10.1002/cbic.202000433. 32633874 PMC7754487

[ref35] Bartsch T.; Becker M.; Rolf J.; Rosenthal K.; Lütz S. Biotechnological production of cyclic dinucleotides-Challenges and opportunities. Biotechnol. Bioeng. 2022, 119, 677–684. 10.1002/bit.28027. 34953086

[ref36] Schwede F.; Genieser H.-G.; Rentsch A. The chemistry of the noncanonical cyclic dinucleotide 2′3′-cGAMP and its analogs. In Non-canonical Cyclic Nucleotides; Seifert R., Ed.; Springer International Publishing: Cham, 2017; pp 359–384. 10.1007/164_2015_43 27392950

[ref37] Milisavljevic N.; Konkolová E.; Kozák J.; Hodek J.; Veselovská L.; Sýkorová V.; Čížek K.; Pohl R.; Eyer L.; Svoboda P.; Růžek D.; Weber J.; Nencka R.; Bouřa E.; Hocek M. Antiviral activity of 7-substituted 7-deazapurine ribonucleosides, monophosphate prodrugs, and triphoshates against emerging RNA viruses. ACS Infect. Dis. 2021, 7, 471–478. 10.1021/acsinfecdis.0c00829. 33395259

[ref38] Zhu X.-F.; Williams H. J.; Ian Scott A. An Improved Transient Method for the Synthesis ofN-Benzoylated Nucleosides. Synth. Commun. 2003, 33, 1233–1243. 10.1081/scc-120017200.

[ref39] Hakimelahi G. H.; Proba Z. A.; Ogilvie K. K. New catalysts and procedures for the dimethoxytritylation and selective silylation of ribonucleosides. Can. J. Chem. 1982, 60, 1106–1113. 10.1139/v82-165.

[ref40] Wei X. Coupling activators for the oligonucleotide synthesis via phosphoramidite approach. Tetrahedron 2013, 69, 3615–3637. 10.1016/j.tet.2013.03.001.

[ref41] Gaffney B. L.; Veliath E.; Zhao J.; Jones R. A. One-flask syntheses of c-di-GMP and the [Rp,Rp] and [Rp,Sp] thiophosphate analogues. Org. Lett. 2010, 12, 3269–3271. 10.1021/ol101236b. 20572672 PMC2905038

[ref42] Hocek M. Synthesis of base-modified 2′-deoxyribonucleoside triphosphates and their use in enzymatic synthesis of modified DNA for applications in bioanalysis and chemical biology. J. Org. Chem. 2014, 79, 9914–9921. 10.1021/jo5020799. 25321948

[ref43] Veliath E.; Kim S.; Gaffney B. L.; Jones R. A. Synthesis and characterization of C8 analogs of c-di-GMP. Nucleosides, Nucleotides Nucleic Acids 2011, 30, 961–978. 10.1080/15257770.2011.624567. 22060558

[ref44] Bourderioux A.; Nauš P.; Perlíková P.; Pohl R.; Pichová I.; Votruba I.; Džubák P.; Konečný P.; Hajdúch M.; Stray K. M.; Wang T.; Ray A. S.; Feng J. Y.; Birkus G.; Cihlar T.; Hocek M. Synthesis and significant cytostatic activity of 7-hetaryl-7-deazaadenosines. J. Med. Chem. 2011, 54, 5498–5507. 10.1021/jm2005173. 21711054

[ref45] Milisavljevič N.; Perlíková P.; Pohl R.; Hocek M. Enzymatic synthesis of base-modified RNA by T7 RNA polymerase. A systematic study and comparison of 5-substituted pyrimidine and 7-substituted 7-deazapurine nucleoside triphosphates as substrates. Org. Biomol. Chem. 2018, 16, 5800–5807. 10.1039/c8ob01498a. 30063056

[ref46] Snášel J.; Nauš P.; Dostál J.; Hnízda A.; Fanfrlík J.; Brynda J.; Bourderioux A.; Dušek M.; Dvořáková H.; Stolaříková J.; Zábranská H.; Pohl R.; Konečný P.; Džubák P.; Votruba I.; Hajdúch M.; Řezáčová P.; Veverka V.; Hocek M.; Pichová I. Structural basis for inhibition of mycobacterial and human adenosine kinase by 7-substituted 7-(het)aryl-7-deazaadenine ribonucleosides. J. Med. Chem. 2014, 57, 8268–8279. 10.1021/jm500497v. 25259627

[ref47] Vavřina Z.; Gutten O.; Smola M.; Zavřel M.; Aliakbar Tehrani Z.; Charvát V.; Kožíšek M.; Boura E.; Birkuš G.; Rulíšek L. Protein-Ligand Interactions in the STING Binding Site Probed by Rationally Designed Single-Point Mutations: Experiment and Theory. Biochemistry 2021, 60, 607–620. 10.1021/acs.biochem.0c00949. 33586948

[ref48] Smola M.; Gutten O.; Dejmek M.; Kožíšek M.; Evangelidis T.; Tehrani Z. A.; Novotná B.; Nencka R.; Birkuš G.; Rulíšek L.; Boura E. Ligand strain and its conformational complexity is a major factor in the binding of cyclic dinucleotides to STING protein. Angew. Chem., Int. Ed. Engl. 2021, 60, 10172–10178. 10.1002/anie.202016805. 33616279 PMC8251555

[ref49] Yi G.; Brendel V. P.; Shu C.; Li P.; Palanathan S.; Cheng Kao C. Single nucleotide polymorphisms of human STING can affect innate immune response to cyclic dinucleotides. PLoS One 2013, 8, e77846 10.1371/journal.pone.0077846. 24204993 PMC3804601

[ref50] Dejmek M.; Šála M.; Brazdova A.; Vanekova L.; Smola M.; Klíma M.; Břehová P.; Buděšínský M.; Dračínský M.; Procházková E.; Zavřel M.; Šimák O.; Páv O.; Boura E.; Birkuš G.; Nencka R. Discovery of isonucleotidic CDNs as potent STING agonists with immunomodulatory potential. Structure 2022, 30, 1146–1156. 10.1016/j.str.2022.05.012. 35690061

[ref51] Shih A. Y.; Damm-Ganamet K. L.; Mirzadegan T. Dynamic Structural Differences between Human and Mouse STING Lead to Differing Sensitivity to DMXAA. Biophys. J. 2018, 114, 32–39. 10.1016/j.bpj.2017.10.027. 29320694 PMC5773749

[ref52] Smola M.; Birkus G.; Boura E. No magnesium is needed for binding of the stimulator of interferon genes to cyclic dinucleotides. Acta Crystallogr., Sect. F: Struct. Biol. Commun. 2019, 75, 593–598. 10.1107/s2053230x19010999. 31475926 PMC6718146

[ref53] Lioux T.; Mauny M.-A.; Lamoureux A.; Bascoul N.; Hays M.; Vernejoul F.; Baudru A.-S.; Boularan C.; Lopes-Vicente J.; Qushair G.; Tiraby G. Design, Synthesis, and Biological Evaluation of Novel Cyclic Adenosine-Inosine Monophosphate (cAIMP) Analogs That Activate Stimulator of Interferon Genes (STING). J. Med. Chem. 2016, 59, 10253–10267. 10.1021/acs.jmedchem.6b01300. 27783523

[ref54] Pimková Polidarová M.; Břehová P.; Kaiser M. M.; Smola M.; Dračínský M.; Smith J.; Marek A.; Dejmek M.; Šála M.; Gutten O.; Rulíšek L.; Novotná B.; Brázdová A.; Janeba Z.; Nencka R.; Boura E.; Páv O.; Birkuš G. Synthesis and biological evaluation of phosphoester and phosphorothioate prodrugs of STING agonist 3′,3′-c-Di(2′F,2′dAMP). J. Med. Chem. 2021, 64, 7596–7616. 10.1021/acs.jmedchem.1c00301. 34019405

[ref55] Seela F.; Ming X. 7-Functionalized 7-deazapurine β-d and β-l-ribonucleosides related to tubercidin and 7-deazainosine: glycosylation of pyrrolo[2,3-d]pyrimidines with 1-O-acetyl-2,3,5-tri-O-benzoyl-β-d or β-l-ribofuranose. Tetrahedron 2007, 63, 9850–9861. 10.1016/j.tet.2007.06.107.

[ref56] Mueller U.; Förster R.; Hellmig M.; Huschmann F. U.; Kastner A.; Malecki P.; Pühringer S.; Röwer M.; Sparta K.; Steffien M.; Ühlein M.; Wilk P.; Weiss M. S. The macromolecular crystallography beamlines at BESSY II of the Helmholtz-Zentrum Berlin: Current status and perspectives. Eur. Phys. J. Plus 2015, 130, 141 10.1140/epjp/i2015-15141-2.

[ref57] Kabsch W. XDS. Acta Crystallogr., Sect. D: Biol. Crystallogr. 2010, 66, 125–132. 10.1107/s0907444909047337. 20124692 PMC2815665

[ref58] Vagin A.; Teplyakov A. Molecular replacement withMOLREP. Acta Crystallogr., Sect. D: Biol. Crystallogr. 2010, 66, 22–25. 10.1107/s0907444909042589. 20057045

[ref59] Winn M. D.; Ballard C. C.; Cowtan K. D.; Dodson E. J.; Emsley P.; Evans P. R.; Keegan R. M.; Krissinel E. B.; Leslie A. G. W.; McCoy A.; McNicholas S. J.; Murshudov G. N.; Pannu N. S.; Potterton E. A.; Powell H. R.; Read R. J.; Vagin A.; Wilson K. S. Overview of theCCP4 suite and current developments. Acta Crystallogr., Sect. D: Biol. Crystallogr. 2011, 67, 235–242. 10.1107/s0907444910045749. 21460441 PMC3069738

[ref60] Adams P. D.; Afonine P. V.; Bunkóczi G.; Chen V. B.; Davis I. W.; Echols N.; Headd J. J.; Hung L.-W.; Kapral G. J.; Grosse-Kunstleve R. W.; McCoy A. J.; Moriarty N. W.; Oeffner R.; Read R. J.; Richardson D. C.; Richardson J. S.; Terwilliger T. C.; Zwart P. H. PHENIX: a comprehensive Python-based system for macromolecular structure solution. Acta Crystallogr., Sect. D: Biol. Crystallogr. 2010, 66, 213–221. 10.1107/s0907444909052925. 20124702 PMC2815670

[ref61] Debreczeni J. E.; Emsley P. Handling ligands with Coot. Acta Crystallogr., Sect. D: Biol. Crystallogr. 2012, 68, 425–430. 10.1107/s0907444912000200. 22505262 PMC3322601

