# Supplementary material for: Design, Synthesis, and Biochemical and Biological Evaluation of Novel 7-Deazapurine Cyclic Dinucleotide Analogues as STING Receptor Agonists
Source: J Med Chem. 2022 Oct 6;65(20):14082–103. doi: 10.1021/acs.jmedchem.2c01305 (PMC9620234; doi:10.1021/acs.jmedchem.2c01305)
Supplement: Supplementary file 1 — jm2c01305_si_001.pdf [file jm2c01305_si_001.pdf]

Supporting Information

Design, Synthesis, and Biochemical and Biological Evaluation of Novel 7-Deazapurine Cyclic Dinucleotide Analogues as STING Receptor Agonists

Zdeněk Vavřina[a,b], Pavla Perlíková[a,c,], Nemanja Milisavljević[a,d], Florian Chevrier[a], Miroslav Smola[a], Joshua Smith[a,e], Milan Dejmek[a], Vojtěch Havlíček[a,d], Miloš Buděšínský[a], Radek Liboska[a], Lenka Vaneková[a,f], Jiří Brynda[a], Evzen Boura[a], Pavlína Řezáčová[a], Michal Hocek[a] and Gabriel Birkuš[a,]**

[a] Institute of Organic Chemistry and Biochemistry, Czech Academy of Sciences, Flemingovo namesti 542, Prague 166 10, Czech Republic; [b] Department of Biochemistry, Faculty of Science, Charles University, Hlavova 2030/8, Prague 128 00, Czech Republic; [c] Department of Organic Chemistry, Faculty of Chemical Technology, University of Chemistry and Technology, Technicka 5, Prague 166 28, Czech Republic; [d] Department of Organic Chemistry, Faculty of Science, Charles University, Hlavova 2030/8, Prague 128 00, Czech Republic; [e] First Faculty of Medicine, Charles University, Katerinska 1660, Prague 121 08, Czech Republic; [f] Department of Cell Biology, Faculty of Science, Charles University, Vinicna 1594/7, Prague 128 43, Czech Republic.

Corresponding author's email:

P.P.: Pavla.Perlikova@vscht.cz

G.B.: Gabriel.Birkus@uochb.cas.cz

Table of Contents:

Synthesis of **3e** S3

Table S1: Crystal data and diffraction data collection and refinement statistics S5

Table S2: Residues missing in the crystallographic models due to disordered maps. S7

Table S3: UPLC purity of CDNs. S7

UPLC traces of CDNs S8

Table S4: Top signals and CC_{50}s for compounds tested on PBMCs S21

Figure S1: Interaction scheme of 2'3'-cGAMP in STING ligand-binding site. S22

Figure S2: 2Fo-Fc map of loops over ligand-binding site depicted at 1σ. S22

Synthesis of **3e**

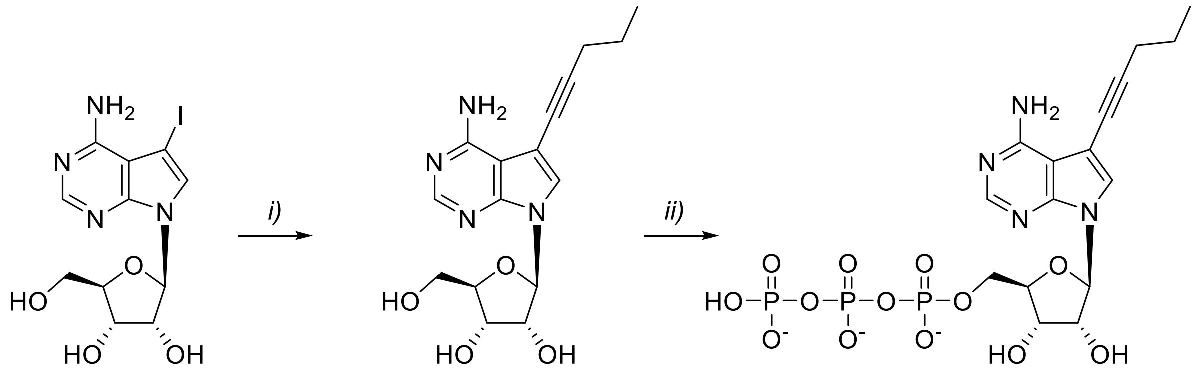

9 **17**, 57% **3e**, 41%

Scheme S1: Reagents and conditions: *i)* pentyne, CuI, Et_3N, $Pd(PPh_3)_4$, DMF *ii)* 1. $POCl_3$, $PO(OMe)_3$, 0 °C, 1.5 h; 2. $(NHBu_3)_2H_2P_2O_7$, Bu_3N, DMF, 0 °C, 2 h; 3. TEAB

7-(Pent-1-yn-1-yl)-7-deazaadenosine (17)

A suspension of 7-iodo-7-deazaadenosine (**9**, 200 mg, 0.51 mmol) and CuI (6 mg, 0.03 mmol) in anhydrous DMF (4 mL) was treated with pentyne (869 mg, 12.75 mmol), anhydrous Et_3N (77 mg, 0.77 mmol), and $Pd(PPh_3)_4$ (30 mg, 0.03 mmol). This mixture was stirred under argon atmosphere at 70 °C for 48 h to form a clear solution. The resulting suspension was evaporated in vacuo, resuspended in methanol, coevaporated with silica gel, and purified by phase flash chromatography (C18 column, 0 to 100% MeOH in water). Product **17** (97 mg, 57%) was isolated as a white solid. R_f = 0.6 (SiO_2; $CHCl_3$/MeOH 5:1). ^{1}H NMR (500.0 MHz, DMSO-d_6): 1.00 (t, 3H, J_{vic} = 7.4, C**H₃**CH₂CH₂); 1.57 (qt, 2H, J_{vic} = 7.4, 7.0, $CH_3C\mathbf{H_2}CH_2$); 2.45 (t, 2H, J_{vic} = 7.0, $CH_3CH_2C\mathbf{H_2}$); 3.53 (ddd, 1H, J_{gem} = 11.9, $J_{5'b,OH}$ = 6.2, $J_{5'b,4'}$ = 3.8, H-5′b); 3.62 (ddd, 1H, J_{gem} = 11.9, $J_{5'a,OH}$ = 5.0, $J_{5'a,4'}$ = 3.8, H-5′a); 3.88 (td, 1H, $J_{4',5a'}$ = $J_{4',5b'}$ = 3.8, $J_{4',3'}$ = 3.2, H-4′); 4.07 (ddd, 1H, $J_{3',2'}$ = 5.0, $J_{3',OH}$ = 4.7, $J_{3',4'}$ = 3.2, H-3′); 4.36 (td, 1H, $J_{2',1'}$ = $J_{2',OH}$ = 6.2, $J_{2',3'}$ = 5.0, H-2′); 5.12 (d, 1H, $J_{OH,3'}$ = 4.7, OH-3′); 5.20 (dd, 1H, $J_{OH,5a'}$ = 5.0, $J_{OH,5b'}$ = 6.2, OH-5′); 5.32 (d, 1H, $J_{OH,2'}$ = 6.2, OH-2′); 6.00 (d, 1H, $J_{1',2'}$ = 6.2, H-1′); 7.66 (s, 1H, H-6); 8.10 (s, 1H, H-2). ^{13}C NMR (125.7 MHz, DMSO-d_6): 15.63 ($\mathbf{C}H_3CH_2CH_2$); 21.05 ($CH_3CH_2\mathbf{C}H_2$); 21.90 ($CH_3\mathbf{C}H_2CH_2$); 61.74 (CH_2-5′); 70.73 (CH-3′); 73.94 (deazaA-**C**≡C-*n*Pr); 74.12 (CH-2′); 85.39 (CH-4′); 87.26 (CH-1′); 92.62 (deazaA-C≡**C**-*n*Pr); 95.65 (C-5); 102.60 (C-4a); 126.06 (CH-6); 149.60 (C-7a); 152.75 (CH-2); 157.79 (C-4). ESI MS m/z (rel. %): 333 (100) $[M+H]^+$, 355 (18) $[M-H+Na]^+$. HR MS (ESI) for $C_{16}H_{21}O_4N_4$ $[M + H]^+$: calcd 333.15573; found 333.15569.

7-(Pent-1-yn-1-yl)-7-deazaadenosine 5'-O-triphosphate sodium salt (3e)

Nucleoside **17** (30 mg, 0.11 mmol) was dissolved in $PO(OMe)_3$ (300 µl). $POCl_3$ (14 µL, 0.14 mmol) was added, and the reaction mixture was stirred for 1.5h. Then, a solution of $(NHBu_3)_2H_2P_2O_7$ (293 mg, 0.54 mmol) and tributyl amine (110 µL, 0.48 mmol) in anhydrous MeCN (1 mL) was added, and stirring continued for another 2 hours. Then, aqueous solution of TEAB (2M, 2 mL, 4 mmol) was added, and the mixture was evaporated under a reduced pressure. The residue was coevaporated with water several times. The product was purified by chromatography on HPLC (C18 column, 0.1M TEAB in water to 0.1M TEAB in 50% aq. MeOH), was coevaporated several times with water, and converted to a sodium salt form (Dowex 50WX8 in a Na^+ cycle). Triphosphate **3e** (21 mg, 41 %) was obtained as a white lyophilizate (water). ^{1}H NMR (500.0 MHz, D_2O, ref(*t*BuOH) = 1.24 ppm): 1.03 (t, 3H, J_{vic} = 7.4, C**H$_3$**CH_2CH_2); 1.64 (qt, 2H, J_{vic} = 7.4, 7.1, CH_3C**H$_2$**CH_2); 2.46 (t, 2H, J_{vic} = 7.1, CH_3CH_2C**H$_2$**); 4.12 (ddd, 1H, J_{gem} = 11.7, $J_{H,P}$ = 4.7, $J_{5'b,4'}$ = 3.2, H-5′b); 4.27 (ddd, 1H, J_{gem} = 11.7, $J_{H,P}$ = 6.8, $J_{5'a,4'}$ = 3.0, H-5′a); 4.34 (dddd, 1H, $J_{4',5'}$ = 3.2, 3.0, $J_{4',3'}$ = 2.7, $J_{H,P}$ = 1.7, H-4′); 4.58 (dd, 1H, $J_{3',2'}$ = 5.4, $J_{3',4'}$ = 2.7, H-3′); 4.68 (dd, 1H, $J_{2',1'}$ = 7.0, $J_{2',3'}$ = 5.4, H-2′); 6.22 (d, 1H, $J_{1',2'}$ = 7.0, H-1′); 7.68 (s, 1H, H-6); 8.16 (s, 1H, H-2). ^{13}C NMR (125.7 MHz, D_2O, ref(*t*BuOH) = 32.43 ppm): 15.78 (**C**$H_3CH_2CH_2$); 23.67 (CH_3CH_2**C**H_2); 24.38 (CH_3**C**H_2CH_2); 68.21 (d, $J_{C,P}$ = 5.6, CH_2-5′); 73.29 (CH-3′); 75.48 (deazaA-**C**≡C-*n*Pr); 76.43 (CH-2′); 86.70 (d, $J_{C,P}$ = 8.8, CH-4′); 88.36 (CH-1′); 97.65 (deazaA-C≡**C**-*n*Pr); 100.57 (C-5); 106.08 (C-4a); 128.00 (CH-6); 152.21 (C-7a); 155.35 (CH-2); 160.65 (C-4). ^{31}P{^{1}H} NMR (202.3 MHz, D_2O): -20.45 (dd, *J* = 19.9, 19.4, P□); -9.68 (d, *J* = 19.4, P□); -4.56 (d, *J* = 19.9, P□). ESI MS m/z (rel. %): 491 (100) $[M-H_2PO_3-H]^-$, 513 (50) $[M-H_2PO_3-2H+Na]^-$, 571 (18) $[M-H]^-$, 593 (29) $[M-2H+Na]^-$, 615 (34) $[M-3H+2Na]^-$. HR MS (ESI) for $C_{16}H_{22}O_{13}N_4P_3$ $[M-H]^-$ calcd 571.04017; found 571.03961.

Table S1: Crystal data and diffraction data collection and refinement statistics

	**STING-5f**	**STING-5k**	**STING-5l**	**STING-5m**
**PDB code**	8A2H	8A2J	8A2I	8A2K
**Data collection statistics**				
Space group	$P\ 2_1\ 2_1\ 2$	$P\ 2_1\ 2_1\ 2$	$P\ 2_1\ 2_1\ 2$	$P\ 2_1\ 2_1\ 2$
Cell parameters (Å; ˚)	95.187 117.98 36.48<br>90.00 90.00 90.00	94.29 116.91 35.98<br>90.00 90.00 90.00	94.12 117.28 35.93<br>90.00 90.00 90.00	93.90 117.26 35.88<br>90.00 90.00 90.00
Wavelength (Å)	1.5418	1.5418	0.9184	0.9184
Resolution (Å)	36.35 - 2.7<br>(2.8 - 2.7)	36.0 - 2.3<br>(2.4 - 2.3)	47.06 - 2.16<br>(2.26-2.16)	49.7-1.9<br>(2.0 – 1.9)
Number of unique reflections	21745 (3435)	32835 (3194)	21423 (2860)	32622 (3180)
Multiplicity	3.3 (3.4)	4.6 (2.6)	6.5 (4.0)	7.9 (7.7)
Completeness (%)	99.2 (95.5)	98.6 (97.1)	96.4 (81.1)	99.9 (99.8)
R_{merge}[a]	7.0 (40.9)	14.8 (38.0)	16.1 (133.1)	14.5 (183.6)
Average I/σ(I)	9.0 (1.6)	7.6 (2.4)	9.1 (1.1)	11.0 (1.0)
CC1/2	99.1 (70.9)	98 (72.9)	99.7 (51.0)	99.9 (42.1)
Wilson B (Å^2)	47.160	39.391	42.118	36.485
**Refinement statistics**				
Resolution range (Å)	36.35 - 2.7<br>(2.8 - 2.7)	36.0 - 2.3<br>(2.4 - 2.3)	47.06 - 2.16<br>(2.26 - 2.16)	49.7 - 1.9<br>(1.957 - 1.890)
No. of reflections in working set	11928 (1122)	17820 (1745)	20347 (1616)	32622 (3021)
No. of reflections in test set	598 (57)	888 (88)	1071 (85)	1632 (159)
R value (%)[b]	0.21 (0.32)	0.22 (0.23)	0.21 (0.32)	0.22 (0.34)

R_{free} value (%)[c]	0.28 (0.37)	0.26 (0.29)	0.25 (0.43)	0.26 (0.37)
RMSD bond length (Å)	0.008	0.014	0.013	0.005
RMSD angle (°)	1.5	1.7	1.6	0.9
Number of atoms in AU (protein/ligand/water molecules)	Total 2953<br>Protein 2800<br>Ligand 51<br>Water 102	Total 2933<br>Protein 784<br>Ligand 57<br>Water 92	Total 3047<br>Protein 2871<br>Ligand 61<br>Water 115	Total 2969<br>Protein 2740<br>Ligand 63<br>Water 149
Mean B value ($Å^2$)	31.17	54.45	41.89	33.62
Ramachandran plot statistics[d]				
Residues in favored regions (%)	96.00	97.46	96.11	97.42
Residues in allowed regions (%)	4.00	2.54	3.89	2.58

The data in parentheses refer to the highest-resolution shell.

[a] Rmerge = $\Sigma_{hkl}\Sigma_i I_i(hkl)$ - $\langle I(hkl)\rangle|/\Sigma_{hkl}\Sigma_i I_i(hkl)$, where the $I_i(hkl)$ is an individual intensity of the ith observation of reflection hkl and $\langle I(hkl)\rangle$ is the average intensity of reflection hkl with summation over all data.

[b] R-value = $||F_o| - |F_c||/|F_o|$, where F_o and F_c are the observed and calculated structure factors, respectively.

[c] R_{free} is equivalent to R value but is calculated for 5 % of the reflections chosen at random and omitted from the refinement process (Brunger, 1992).

[d] as determined by Molprobity

Table S2: Residues missing in the crystallographic models due to disordered maps.

Complex	Chain	N-term	α1-β1 loop	α4-β7 loop	β7-α5 loop	C-term
**5f**	A	140-152			317-318	336-379
	B	140-154	186-192		318	340-379
**5k**	A	140-150			317-318	337-379
	B	140-153	188-192		317	336-379
**5l**	A	140-147			317-320	338-379
	B	140-153	188-192		318	336-379
**5m**	A	140-147			318-319	338-379
	B	140-153	184-195	306	317-318	336-379

Table S3: UPLC purity of CDNs.

Compound	Retention time [min]	Purity [%]	Compound	Retention time [min]	Purity [%]
**4b**	0.77	93.9	**5l**	3.12	100
**5a**	0.89	98.67	**5m**	3.34	100
**5b**	1.93	92.24	**5n**	2.22	100
**5c**	1.58	97.18	**5o**	1.98	99.98
**5d**	1.58	97.2	**5p**	1.76	100
**5e**	0.87	87.79	**5q**	1.78	100
**5f**	1.36	98.67	**5r**	2.32	100
**5g**	1.87	100	**6f**	1.31	97
**5h**	1.91	99.53	**7a**	1.35	98.57
**5i**	2.23	100	**7f**	1.53	99.79
**5j**	2.51	99.47	**8a**	1.43	100
**5k**	2.38	99.71	**8f**	1.89	100

UPLC traces of CDNs.

Compound **4b**:

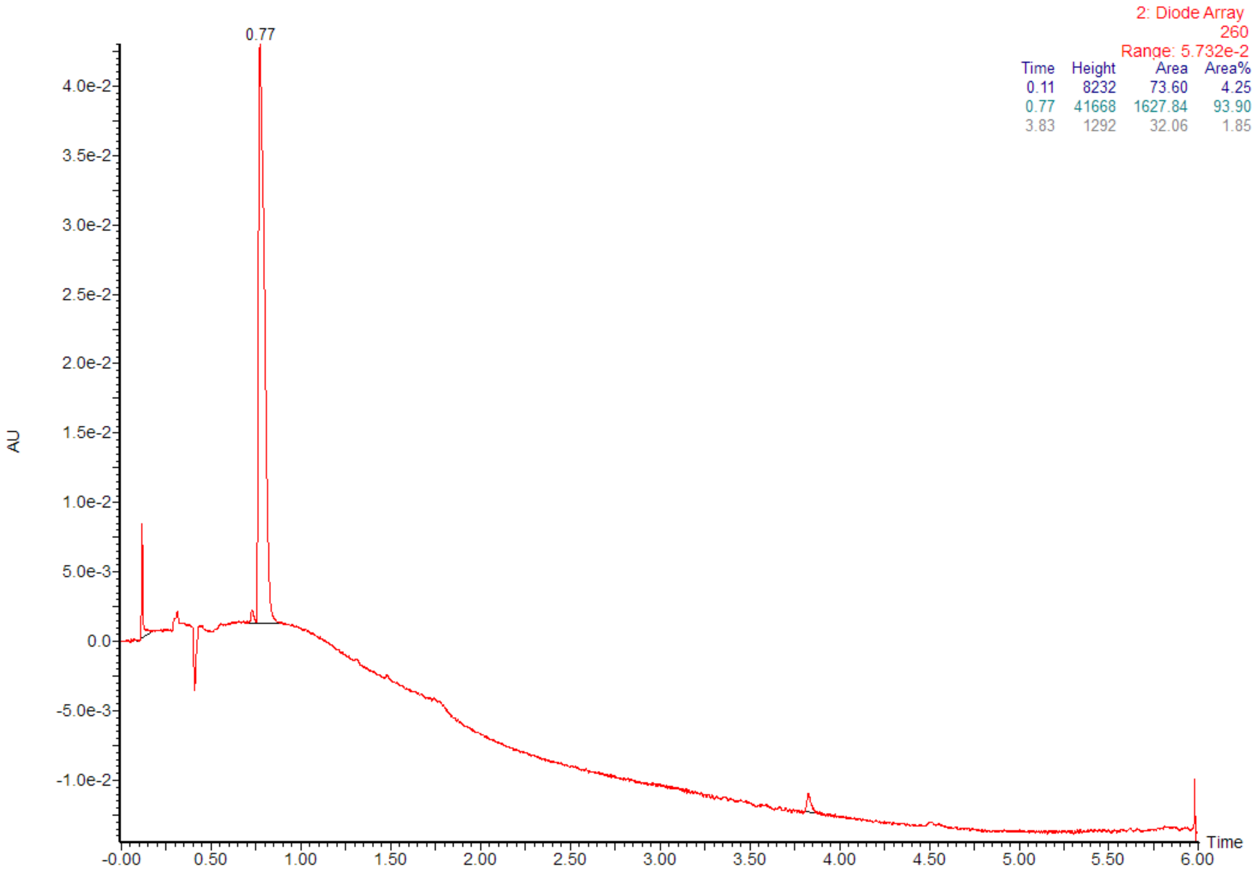

Compound **5a**:

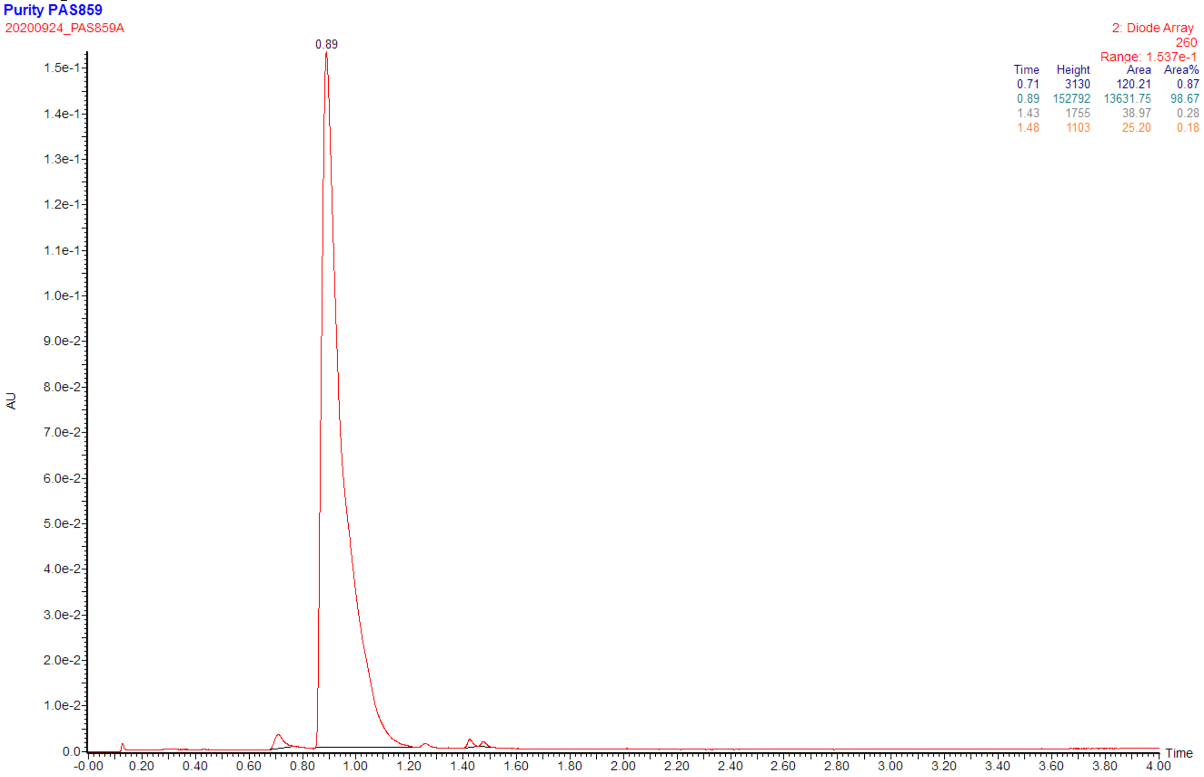

Compound **5b**:

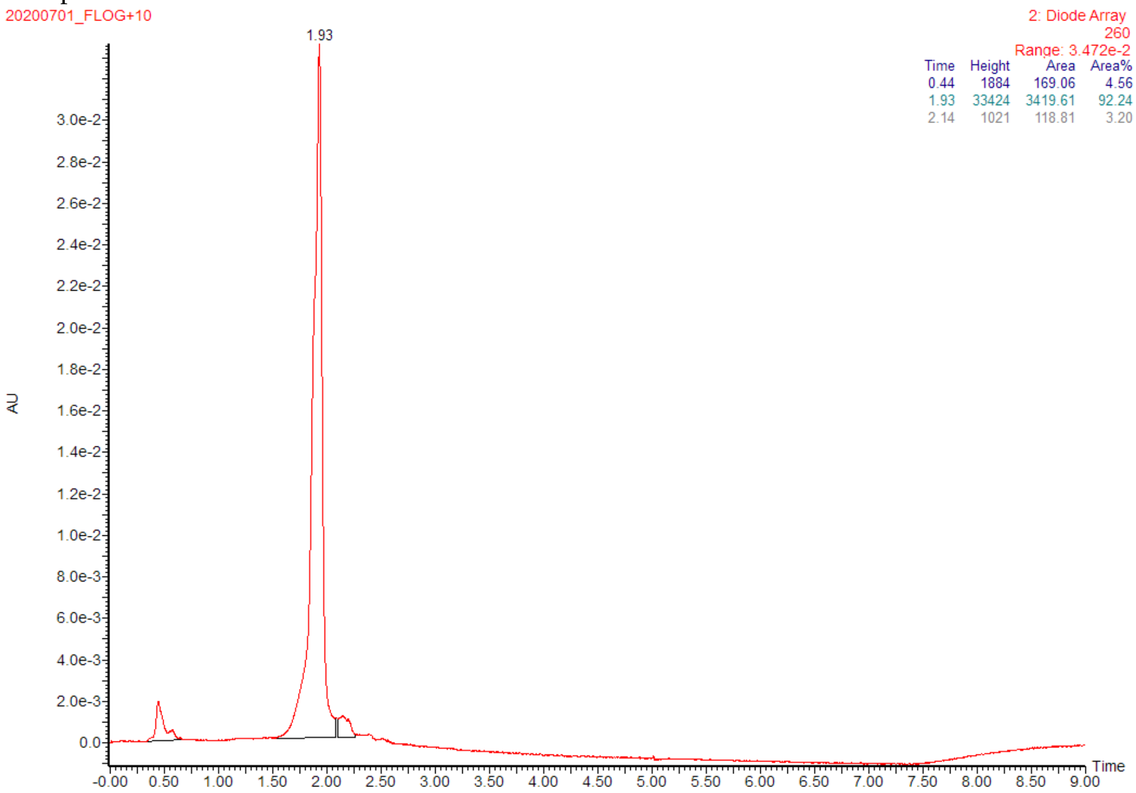

Compound **5c**:

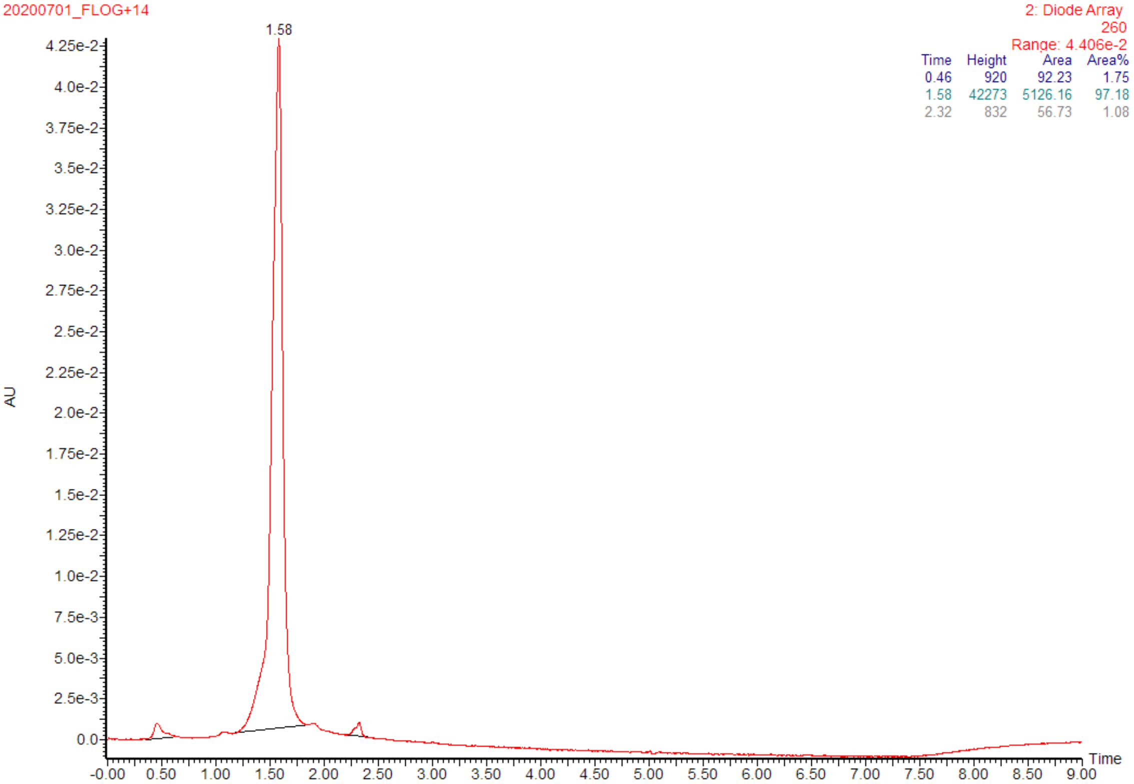

Compound **5d**:

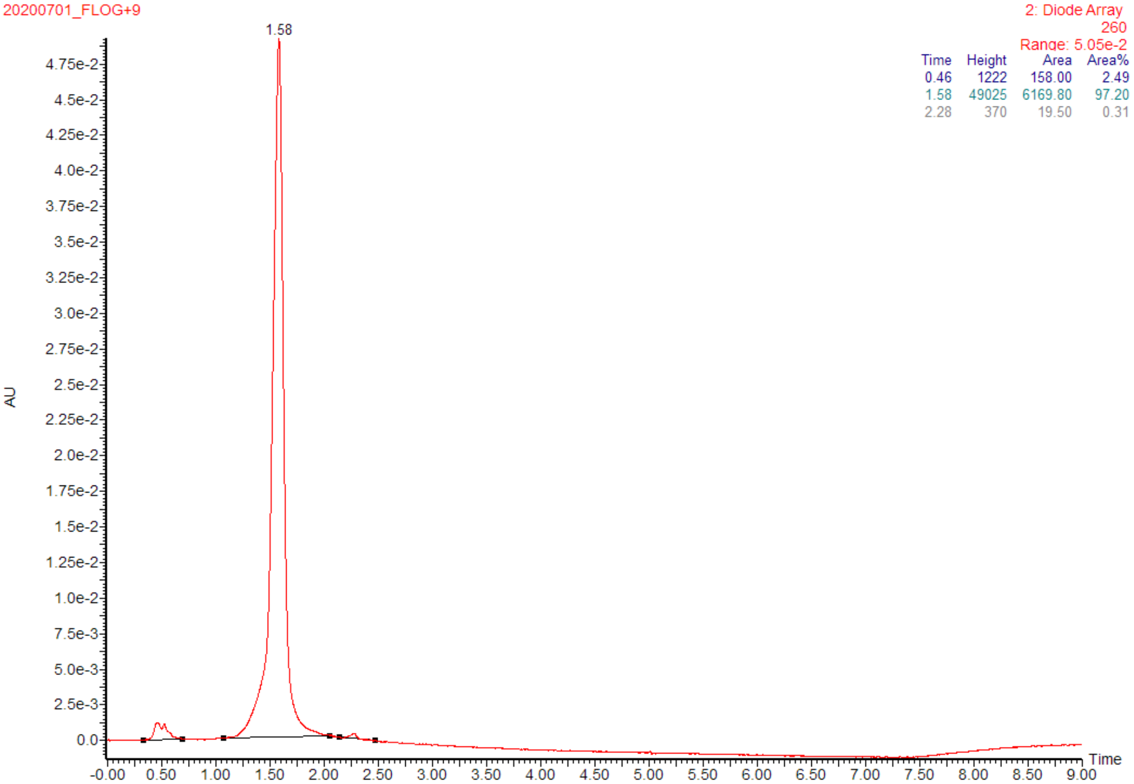

Compound **5e**:

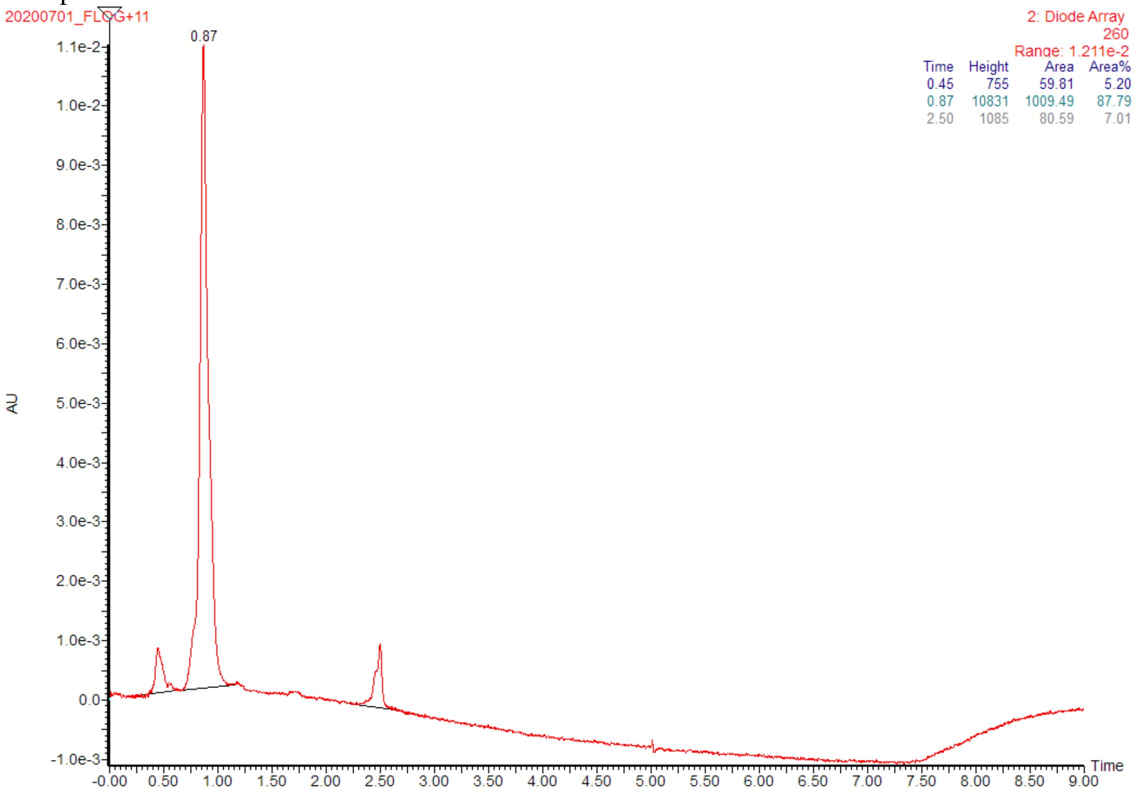

Compound **5f**:

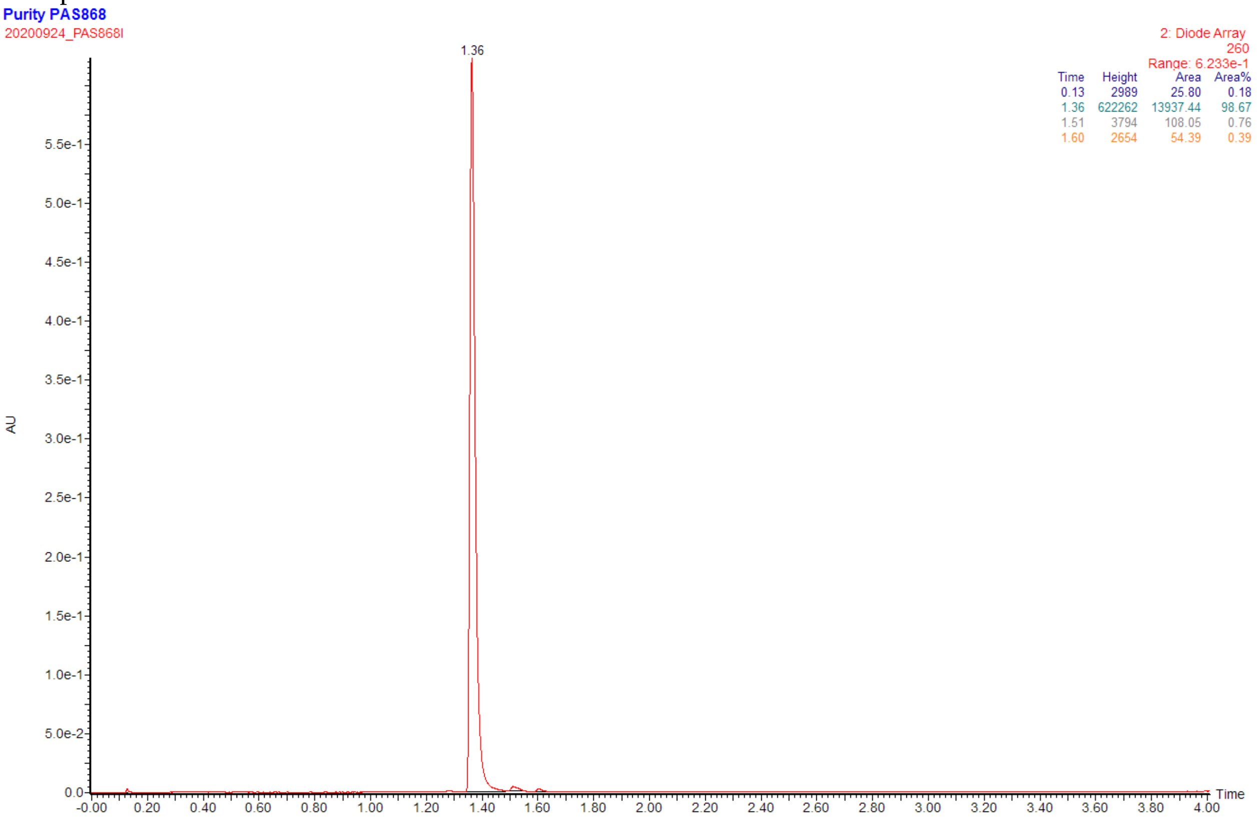

Compound **5g**:

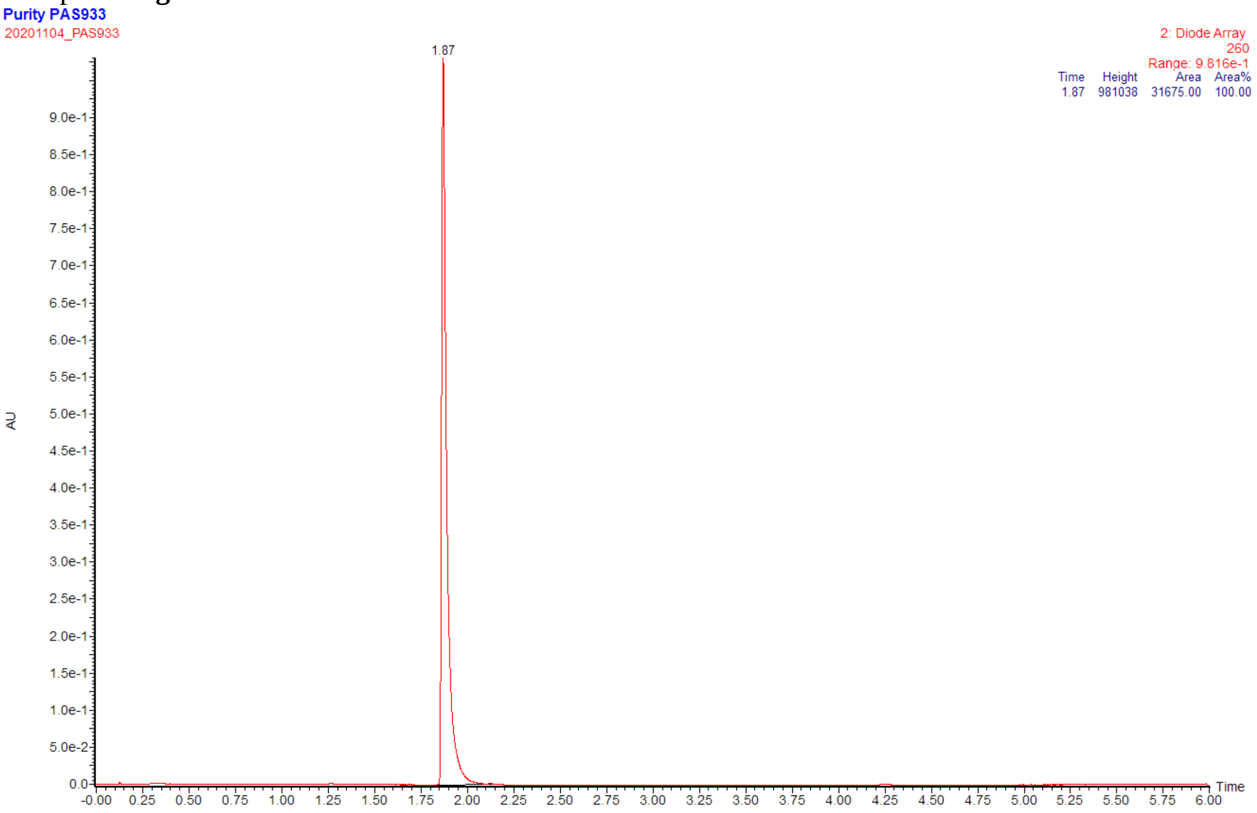

Compound **5h**:

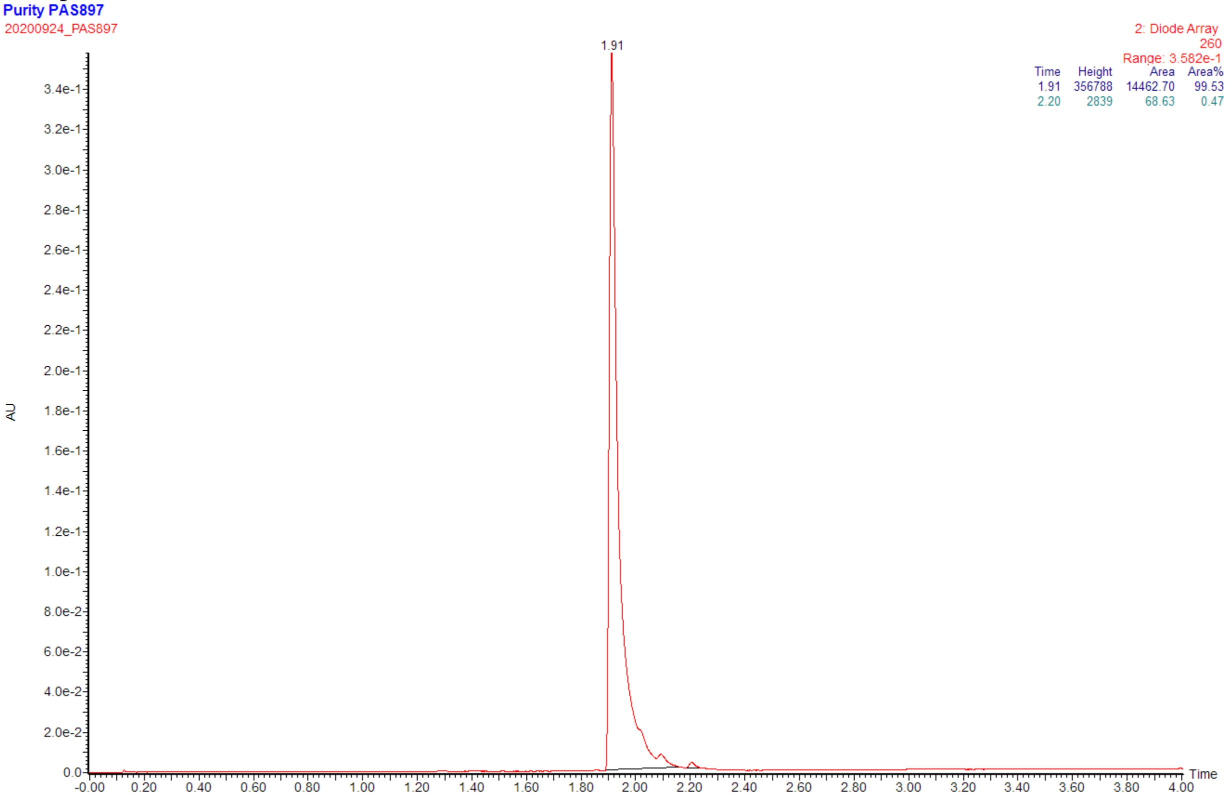

Compound **5i**:

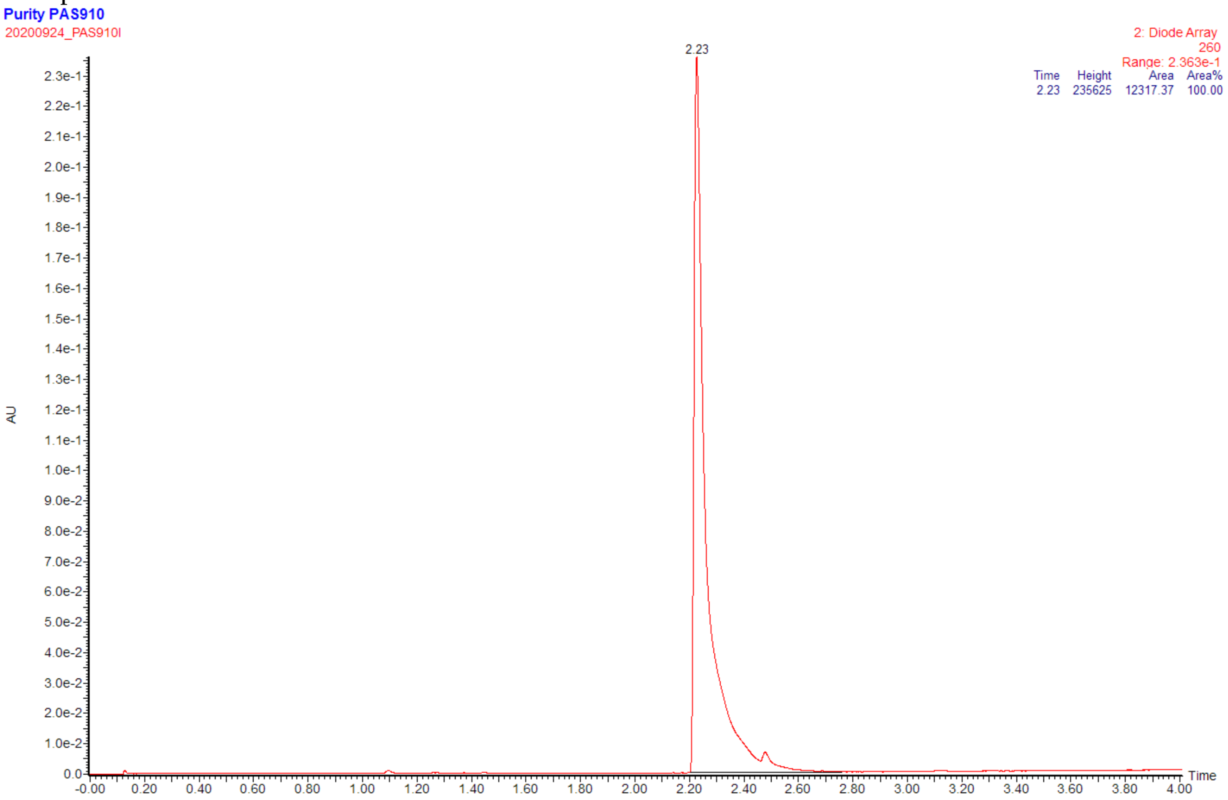

Compound **5j**:

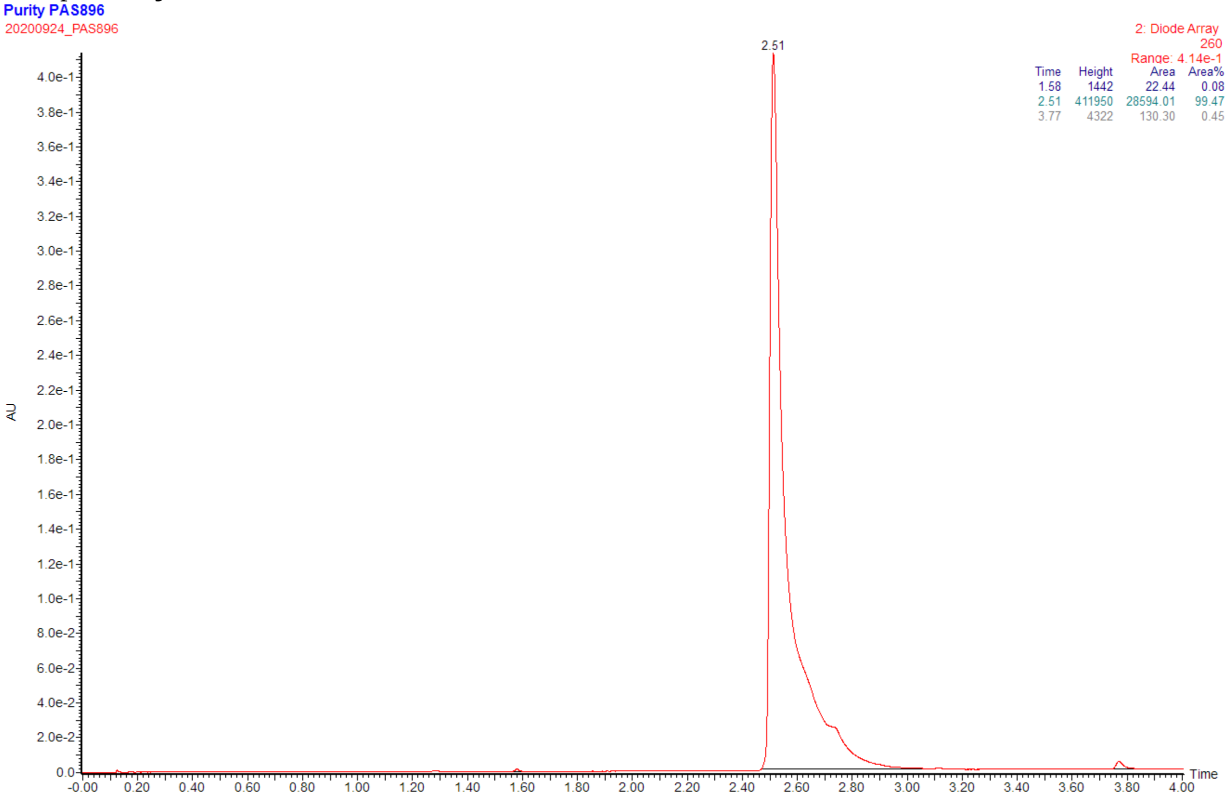

Compound **5k**:

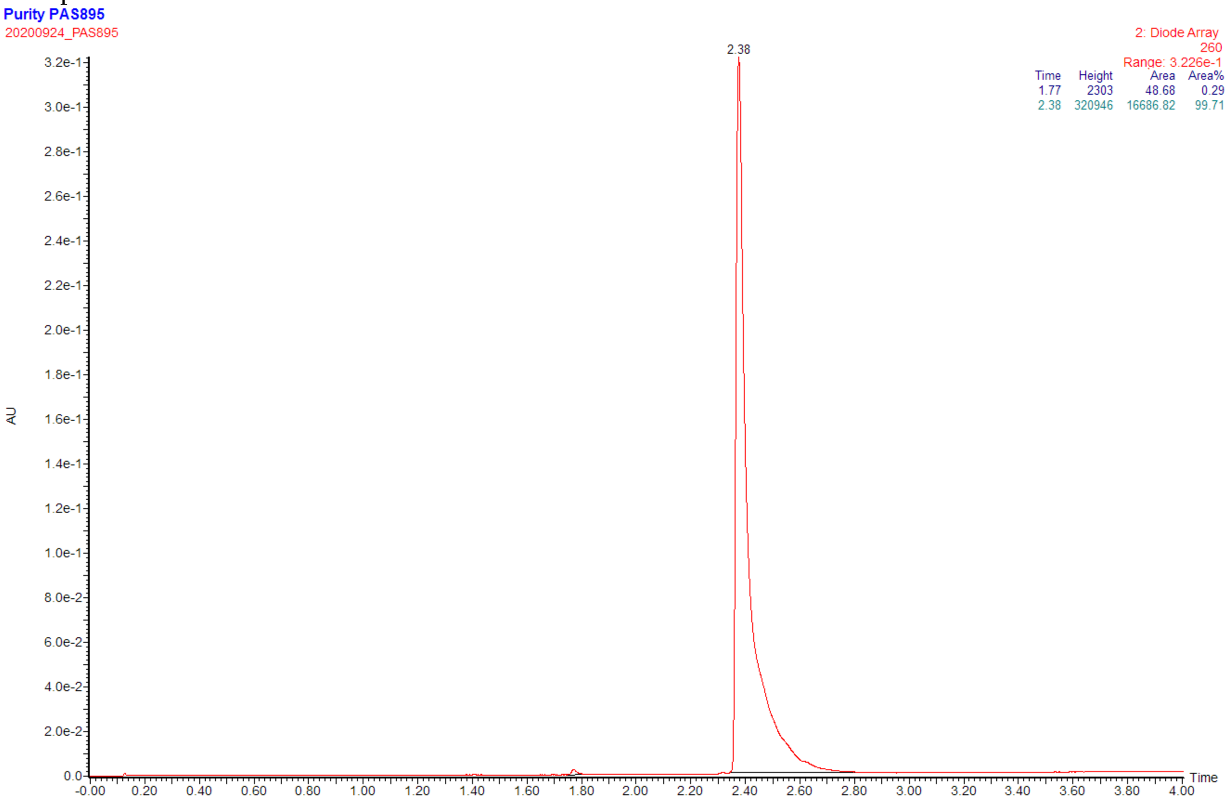

Compound **5l**:

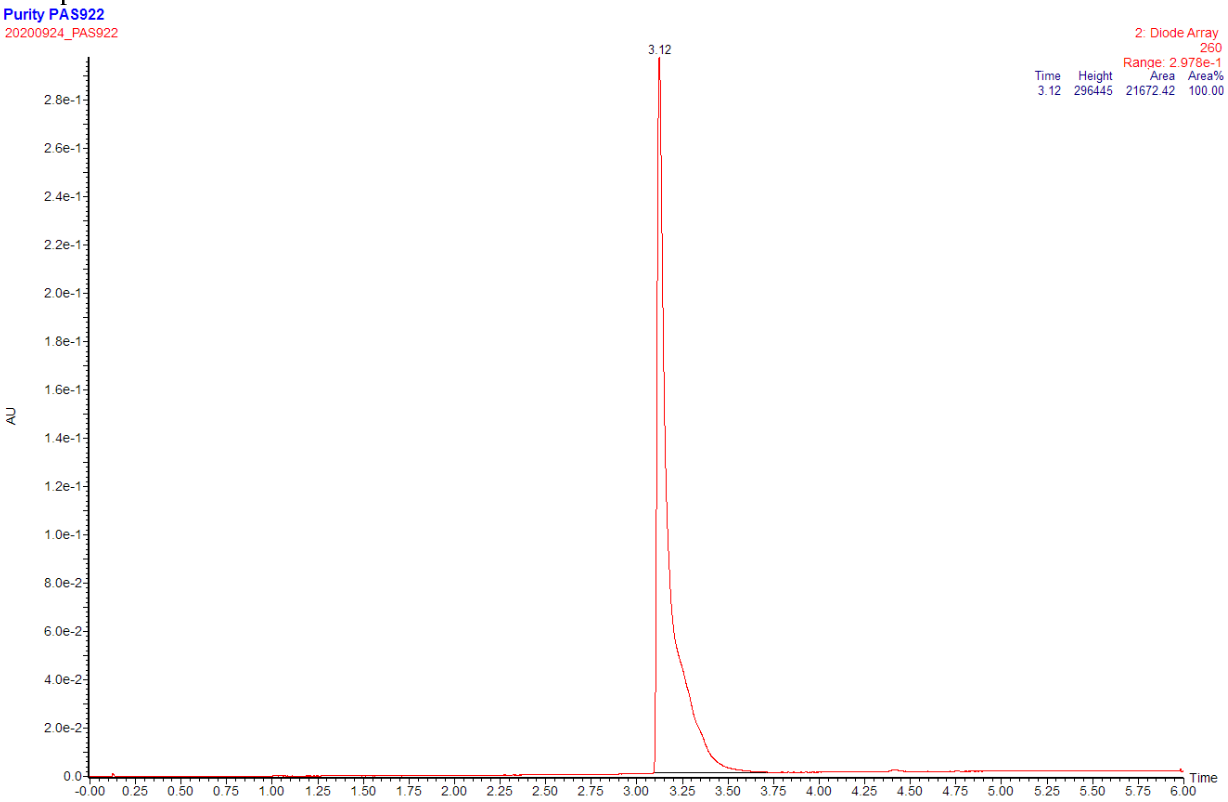

Compound **5m**:

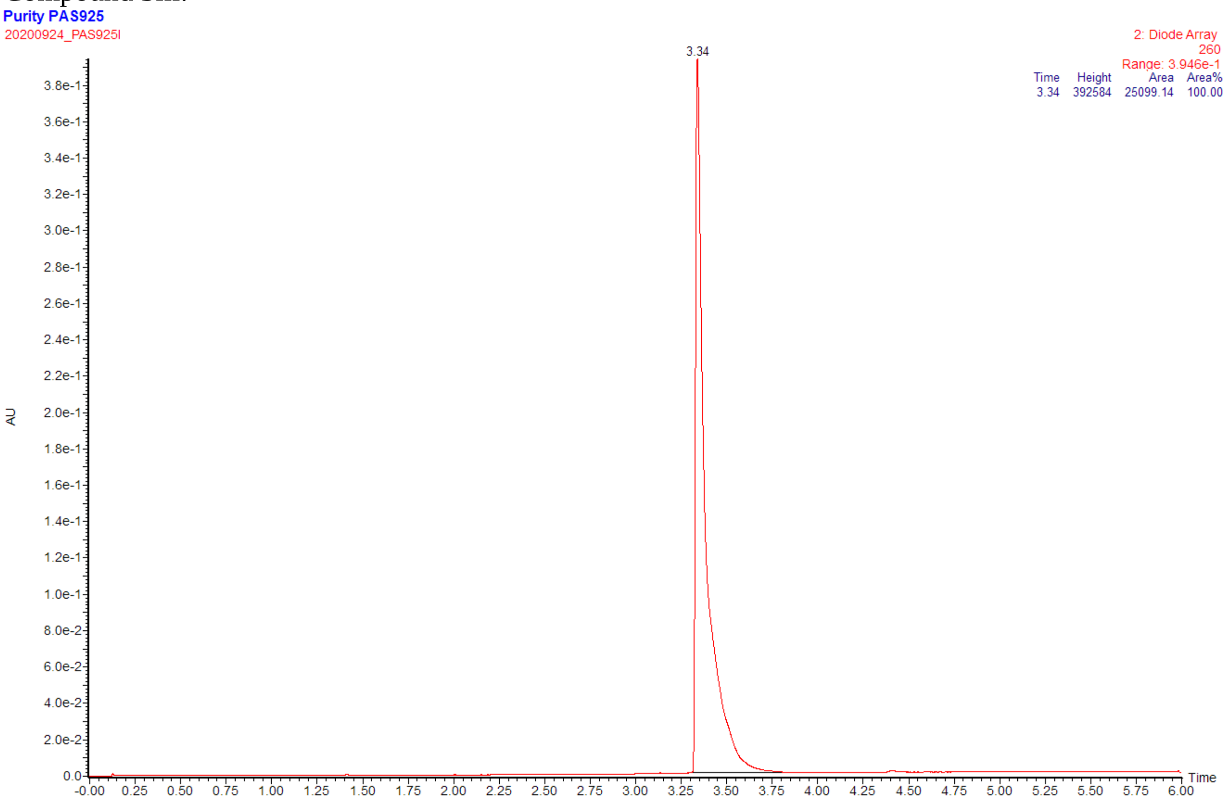

Compound **5n**:

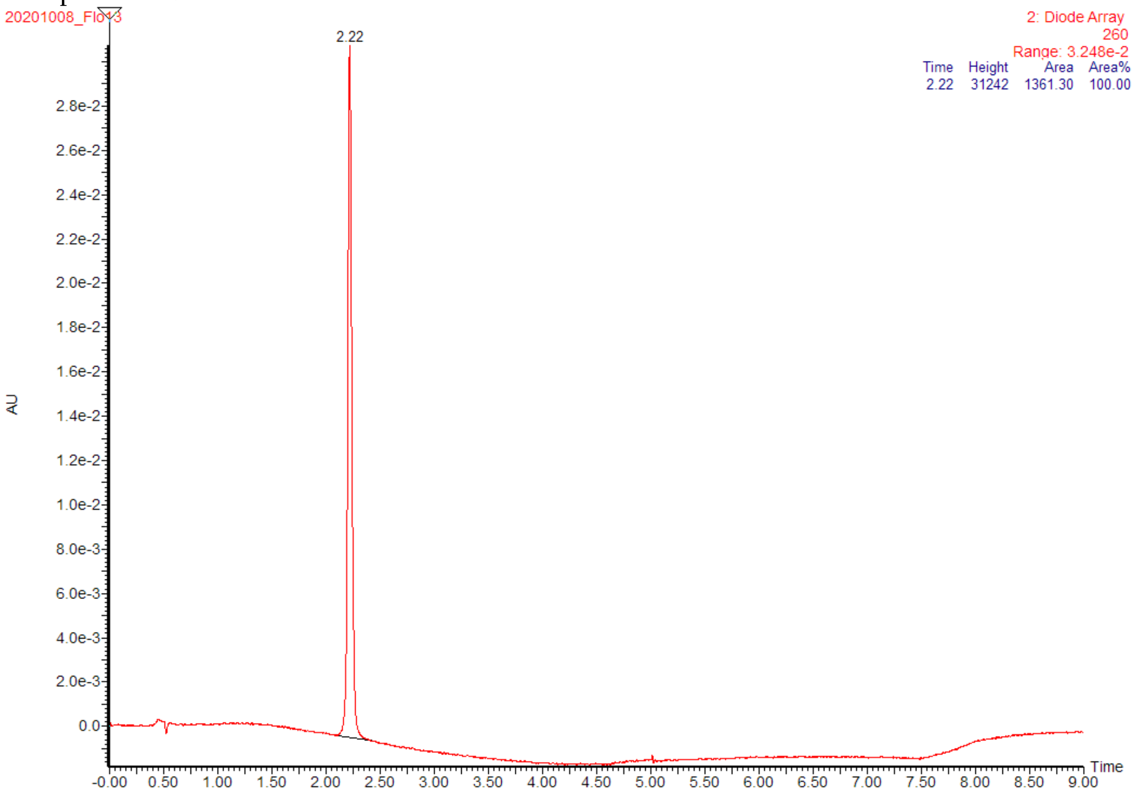

Compound **5o**:

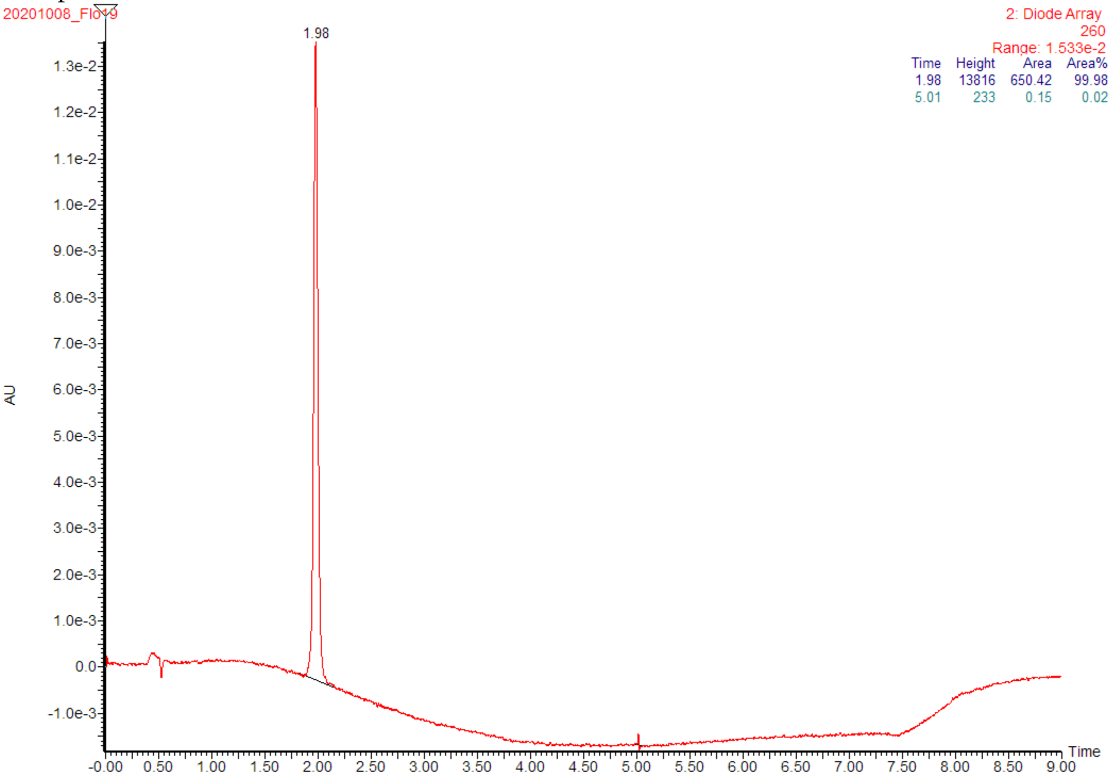

Compound **5p**:

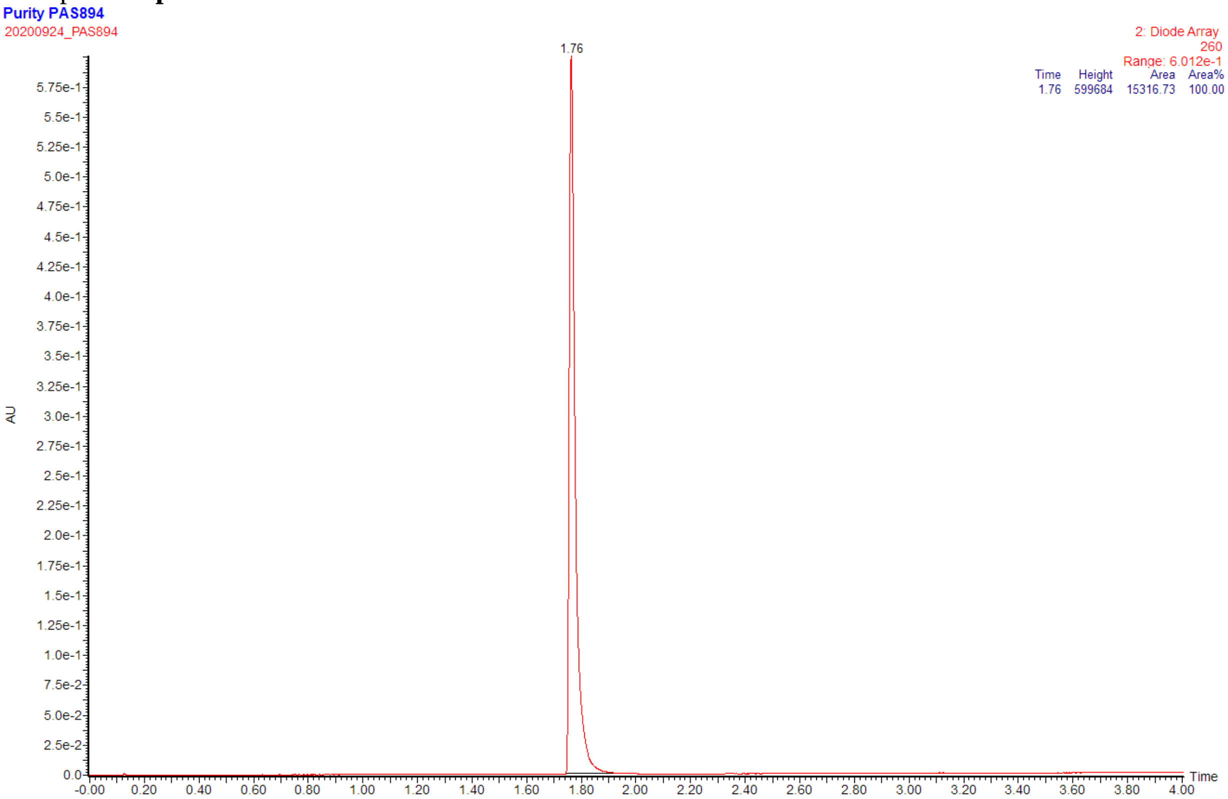

Compound **5q**:

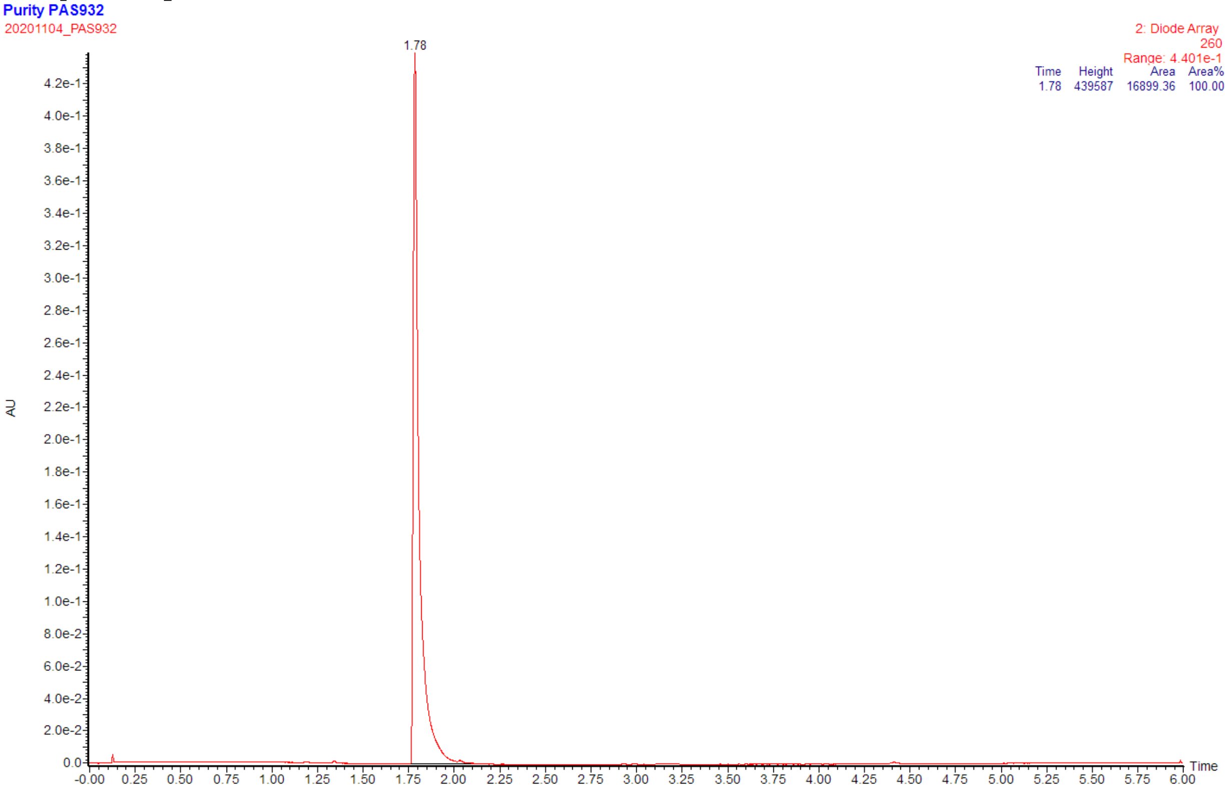

Compound **5r**:

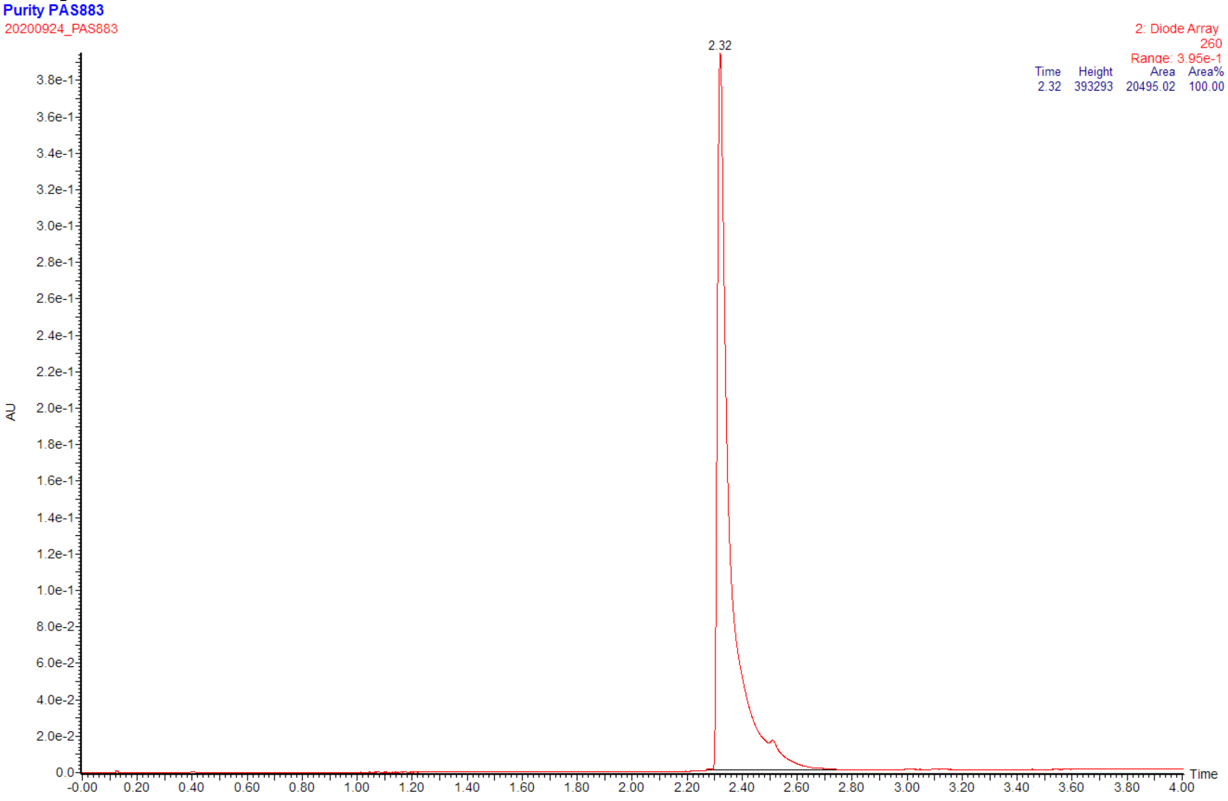

Compound **6f**:

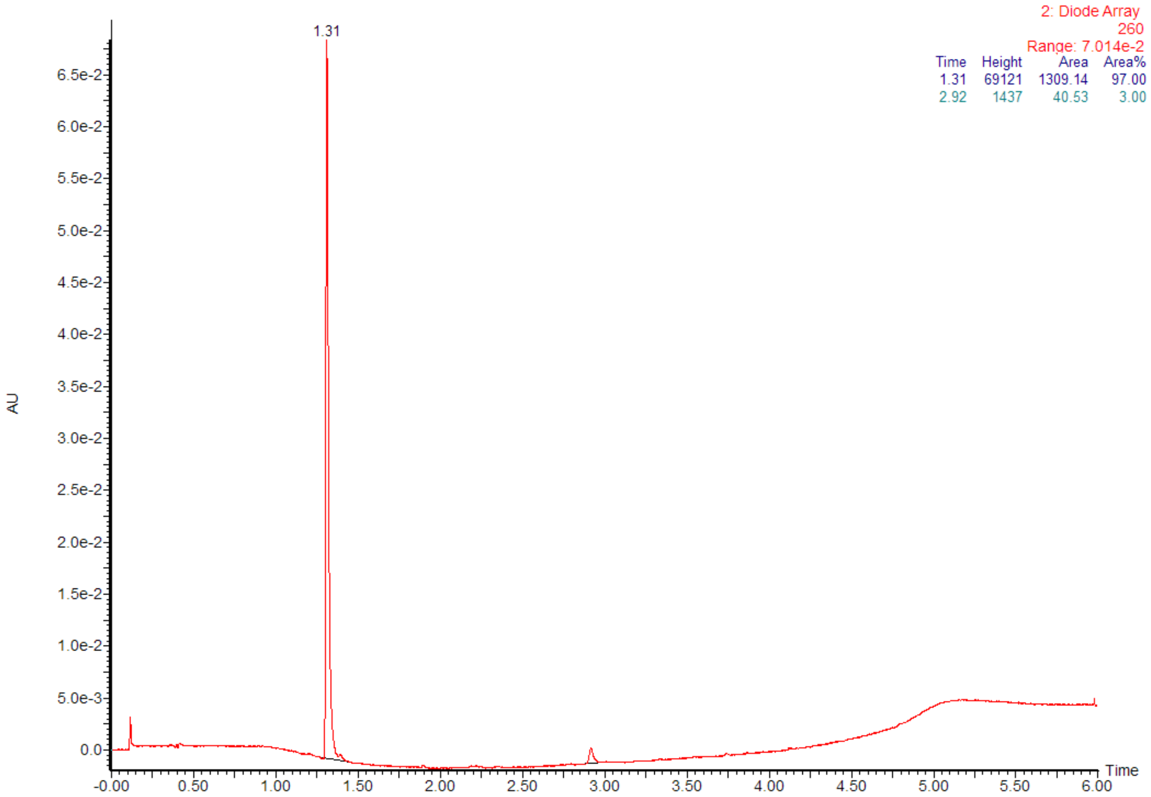

Compound **7a**:

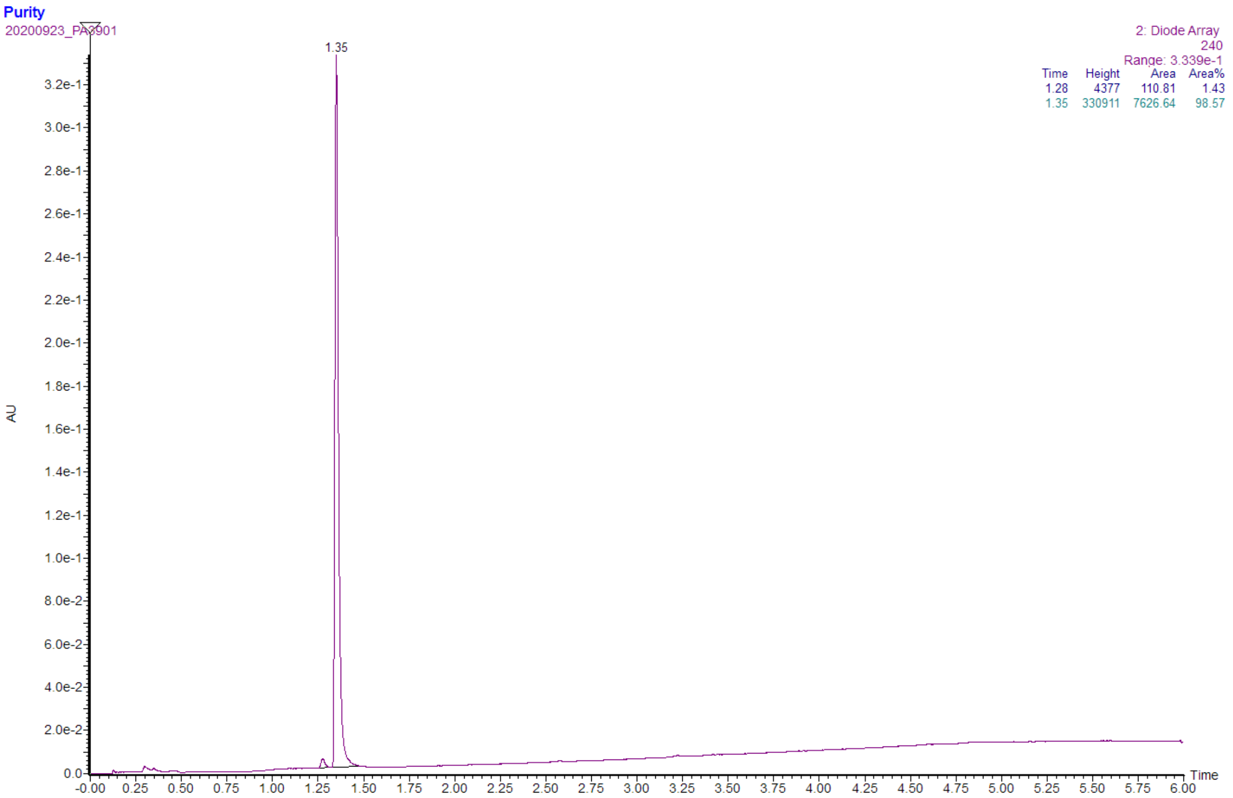

Compound **7f**:

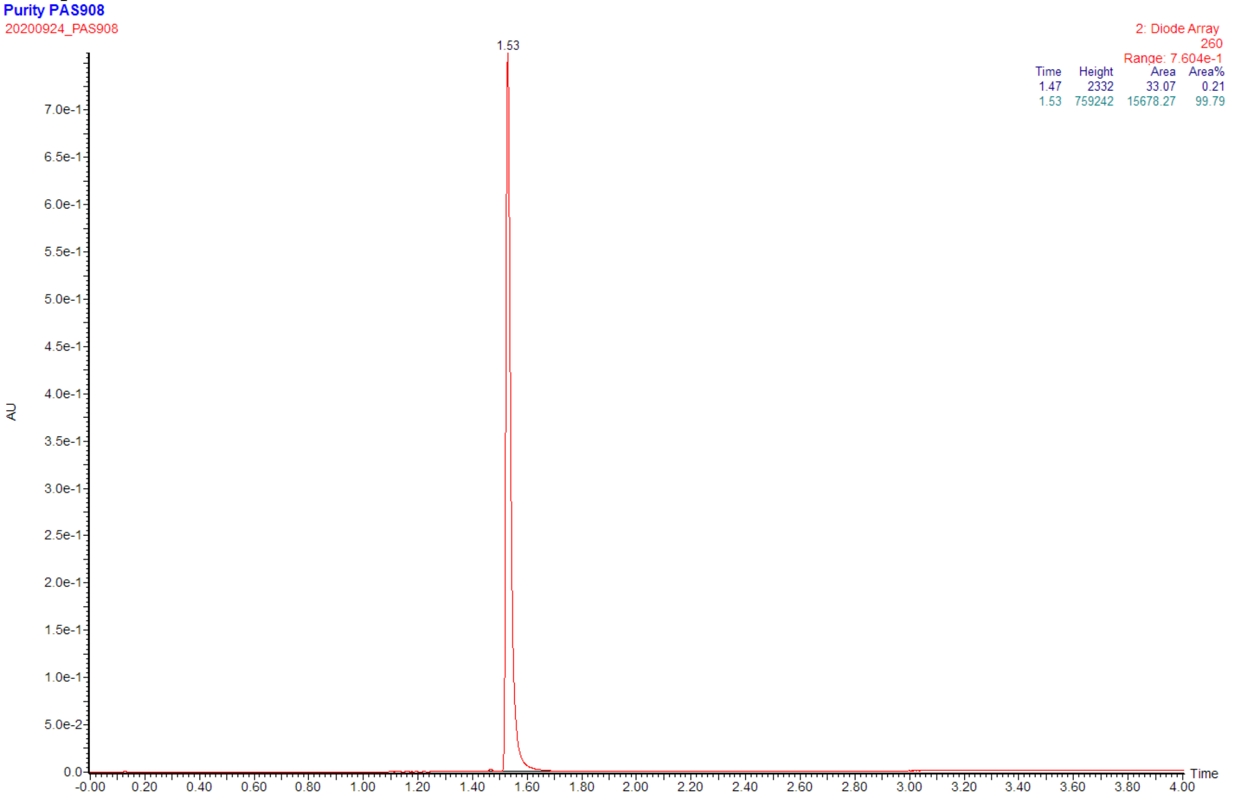

Compound **8a**:

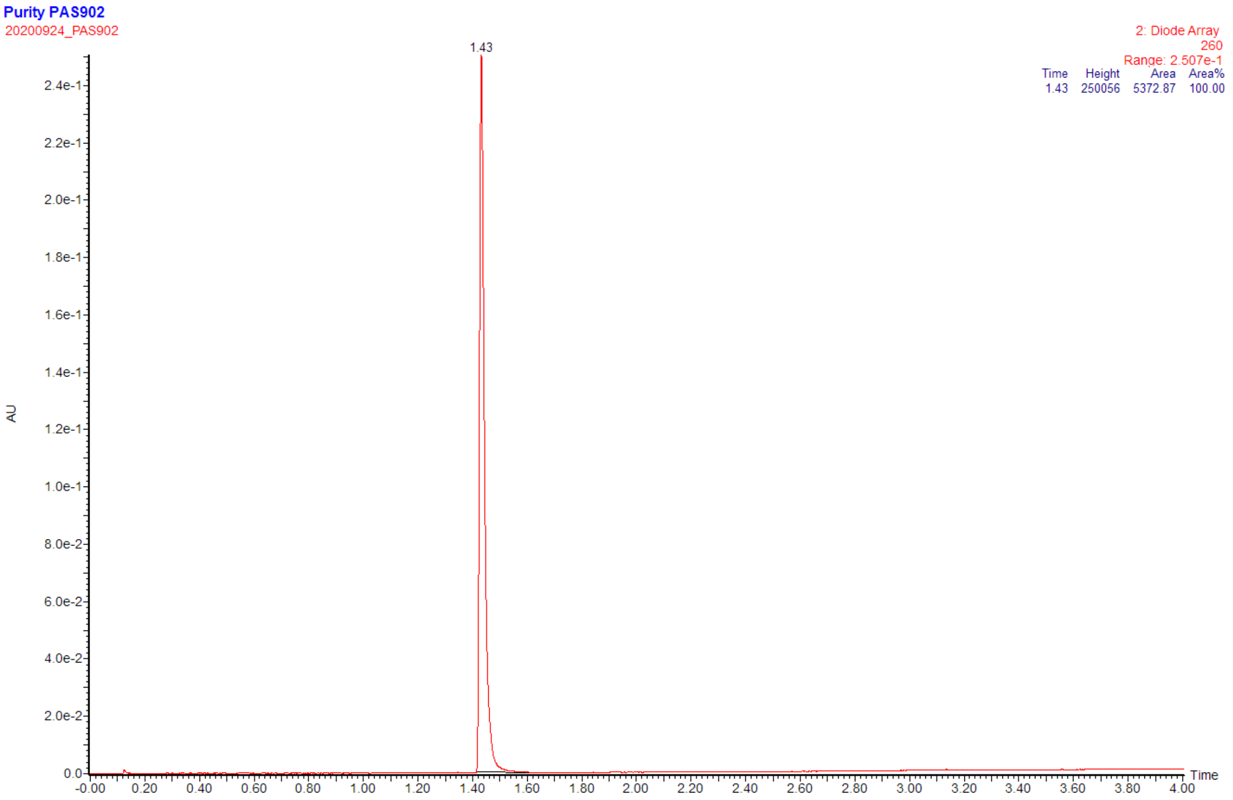

Purity PAS902
20200924_PAS902
2: Diode Array
260
Range: 2.507e-1
Time Height Area Area%
1.43 250056 5372.87 100.00
1.43
AU
Time

Compound **8f**:

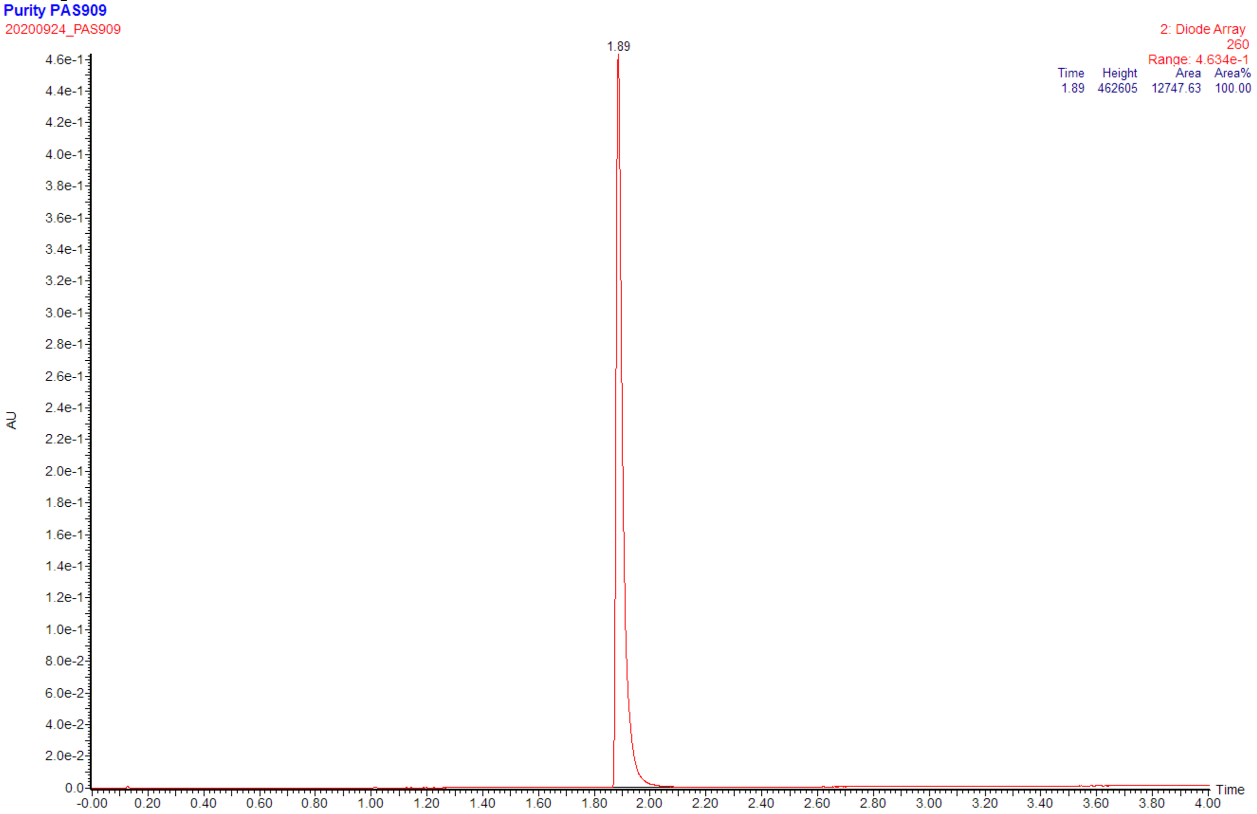

Purity PAS909
20200924_PAS909
2: Diode Array
260
Range: 4.634e-1
Time Height Area Area%
1.89 462605 12747.63 100.00
1.89
AU
Time

Table S4: Top signals and CC_{50}s for compounds tested on PBMCs.

Compound	Top[a]			CC_{50}
	IFNγ	TNFα	IFNα	(μM)
**5b**	2.6	3.9	1.0	>200
**5c**	0.6	1.5	0.2	>200
**5f**	1.7	4.0	3.1	>200
**5g**	1.3	2.9	2.8	>200
**5h**	1.9	4.2	5.6	>200
**5i**	1.8	4.2	2.9	>200
**5j**	2.9	6.5	3.2	>200
**5k**	0.8	1.6	1.0	>200
**5l**	0.8	2.1	1.7	>200
**5m**	1.2	3.5	1.8	>200
**5n**	1.8	2.2	0.6	>200
**5o**	2.1	0.9	0.3	>200
**5p**	1.2	2.6	2.2	>200
**5q**	0.3	1.0	0.6	>200
**5r**	1.6	4.8	5.0	>200
**7f**	0.9	2.1	2.3	>200
*2'3'-c*GAMP	1.0	1.0	1.0	>200
*3'3'-c*GAMP	1.8	1.6	5.4	>200
*2'2'-c*GAMP	1.3	1.8	1.9	>200

[a]Maximal measured concentration of IFNα, TNFα and IFNγ, relative to folds of concentration measured for *2'3'-c*GAMP. The amount of cytokines induced by *2'3'-c*GAMP treatment: INF γ, 10482 pg/mL; TNFα, 1878 pg/mL; INF α, 710 pg/mL. Values are the mean of three independent experiment (n=3), each of them performed on PBMCs from different donor, measured in triplicates

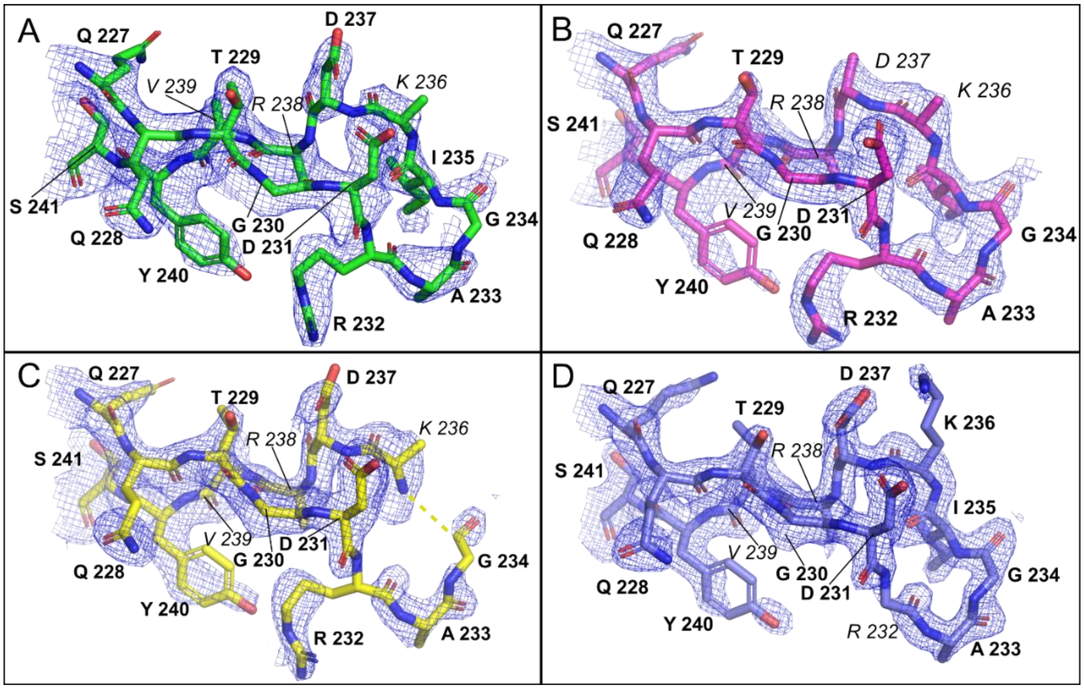

Figure S1: 2*Fo-Fc* map of loops over ligand-binding site depicted at 1σ. (A) **5f**, (B) **5k**, (C) **5l** and (D) **5m.**

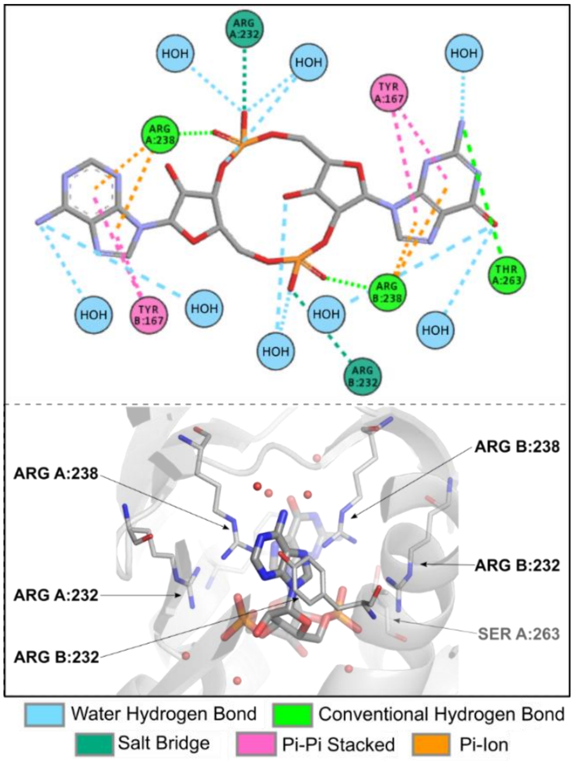

Figure S2: Interaction scheme of *2′3′-c*GAMP in STING ligand-binding site.
